# Mitochondrial Protection and Neural Repair via ROS‐Responsive Biomimetic Nanoparticles in Intracerebral Hemorrhage

**DOI:** 10.1002/advs.78075

**Published:** 2026-10-11

**Authors:** Ling Gao, Li Peng, Xiaobin Zheng, Cong Tan, Chuang Wang, Qijiang Xiong, Weiming Chen, Zhao Yang

**Affiliations:** ^1^ Haikou Research Institute of Xiangya School of Medicine Central South University Haikou China; ^2^ Department of Neurosurgery The First Affiliated Hospital of Hainan University Haikou China; ^3^ Department of Ophthalmology The First Affiliated Hospital of Hainan University Haikou China; ^4^ Department of Neurology The Affiliated Yongchuan Hospital of Chongqing Medical University Chongqing China

**Keywords:** biomimetic membrane, intracerebral hemorrhage, mitochondrial function, neurorepair, reactive oxygen species‐responsive nanoplatform, voltage‐dependent anion channel 1

## Abstract

Intracerebral hemorrhage (ICH)‐induced secondary brain injury is driven in part by oxidative stress, blood‐brain barrier disruption, mitochondrial dysfunction, and neuronal loss. Here, we developed a brain endothelial cell membrane‐coated ROS‐responsive biomimetic nanoplatform (NP@D+M) for co‐delivery of the VDAC1 inhibitor DIDS and the mitochondrial fission inhibitor Mdivi‐1. NP@D+M showed uniform size, preserved endothelial membrane proteins, and accelerated drug release under elevated H2O2. In ICH‐like neuronal injury models, NP@D+M enhanced uptake, protected neurites, reduced LDH release and apoptosis, restored mitochondrial membrane potential and ATP production, and suppressed mitochondrial ROS. Mechanistically, NP@D+M disrupted aberrant LPAR1‐VDAC1 interaction, inhibited mPTP opening, and restored mitochondrial homeostasis; LPAR1 gain‐ and loss‐of‐function experiments confirmed its role in the mitochondrial protective effect. Targeted metabolomics and single‐cell transcriptomics further supported improved energy metabolism and attenuated injury‐associated cellular responses. In vivo, NP@D+M crossed the BBB, accumulated in brain parenchyma, protected perihematomal neurons, improved mitochondrial function, and alleviated neurological and cognitive deficits without obvious systemic toxicity. These findings identify a LPAR1‐VDAC1‐mPTP‐targeted nanotherapeutic strategy for mitochondrial protection and neural repair after ICH.

## Graphical abstract

A ROS‐responsive biomimetic nanoparticle platform co‐delivers DIDS and Mdivi‐1 to protect mitochondria and promote neural repair after intracerebral hemorrhage. By targeting LPAR1/VDAC1‐related mitochondrial dysfunction, the nanotherapy reduces oxidative injury, neuronal apoptosis, and blood‐brain barrier disruption, improving neurological recovery and cognitive performance in experimental models.

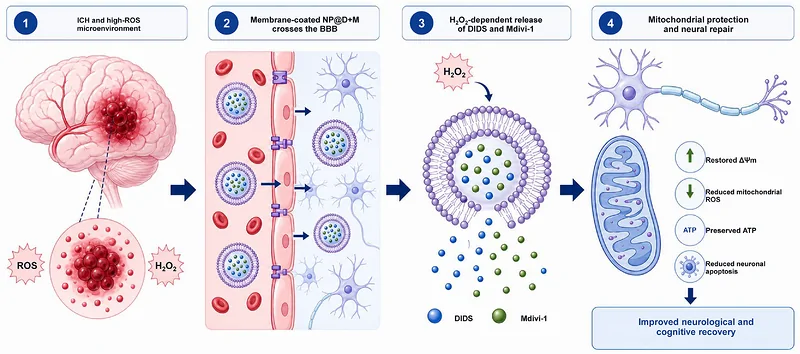

**Abbreviations**
ALT: alanine aminotransferase
AST: aspartate aminotransferase
ATP: adenosine triphosphate
BBB: blood–brain barrier
BCA: bicinchoninic acid
BSA: bovine serum albumin
BUN: blood urea nitrogen
CCK‐8: cell counting kit‐8
Co‐IP: co‐immunoprecipitation
Cr: creatinine
DAB: diaminobenzidine
DAPI: 4',6‐Diamidino‐2‐Phenylindole
DCFH‐DA: 2',7'‐Dichlorodihydrofluorescein Diacetate
DCM: dichloromethane
DEGs: differentially expressed genes
DIDS: 4,4'‐Diisothiocyanatostilbene‐2,2'‐Disulfonic Acid
DLS: dynamic light scattering
DMEM: Dulbecco's modified eagle medium
DMSO: Dimethyl Sulfoxide
EE: encapsulation efficiency
ECL: enhanced chemiluminescence
FBS: fetal bovine serum
FC: fold change
FDR: false discovery rate
Hb: hemoglobin
H&E: hematoxylin and eosin
HRP: horseradish peroxidase
ICH: intracerebral hemorrhage
IOD: integrated optical density
IVIS: in vivo imaging system
JC‐1: 5,5′,6,6′‐Tetrachloro‐1,1′,3,3′‐Tetraethylbenzimidazolylcarbocyanine Iodide
LC‐MS/MS: liquid chromatography‐tandem mass spectrometry
LDH: lactate dehydrogenase
LPS: lipopolysaccharide
LPAR1: lysophosphatidic acid receptor 1
Mdivi‐1: mitochondrial division inhibitor 1
mPTP: mitochondrial permeability transition pore
MRM: multiple reaction monitoring
ΔΨm: mitochondrial membrane potential
PBS: phosphate‐buffered saline
PCA: principal component analysis
PDI: polydispersity index
PLGA‐PEG: poly(lactic‐co‐glycolic acid)‐polyethylene glycol
PVDF: polyvinylidene fluoride
PVA: polyvinyl alcohol
RIPA: radio‐immunoprecipitation assay
ROI: region of interest
ROS: reactive oxygen species
SBI: secondary brain injury
scRNA‐seq: single‐cell RNA sequencing
SDS‐PAGE: sodium dodecyl sulfate‐polyacrylamide gel electrophoresis
TCA: tricarboxylic acid
TEM: Transmission electron microscopy
t‐SNE: t‐distributed stochastic neighbor embedding
UMAP: uniform manifold approximation and projection
VDAC1: voltage‐dependent anion channel 1
WB: Western blot

## 1 Introduction

Intracerebral hemorrhage (ICH) is an acute and critical neurological condition associated with extremely high rates of disability and mortality [1, 2]. Survivors often experience profound neurological deficits and cognitive impairments, imposing significant burdens on both families and healthcare systems [3, 4]. Mounting evidence has identified secondary brain injury (SBI) as a major contributor to ongoing neurological deterioration following ICH. However, effective therapeutic options targeting SBI remain largely unavailable in clinical settings [5, 6]. SBI is driven by a complex interplay of pathological processes, including inflammation, oxidative stress, apoptosis, necrosis, mitochondrial dysfunction, and blood–brain barrier (BBB) disruption. Among these, mitochondrial dysfunction is increasingly recognized as a pivotal factor in neuronal damage and functional decline post‐ICH [7, 8]. Although the central role of mitochondria in ICH pathogenesis is becoming clearer, targeted strategies to restore mitochondrial function and mitigate secondary neuronal injury are still lacking [9, 10, 11, 12]. Consequently, the development of novel, mitochondria‐targeted therapeutic strategies capable of operating efficiently within the pathological milieu of ICH has emerged as a critical research priority in the field [6].

Recent studies have deepened our understanding of mitochondrial involvement in secondary injury following ICH [9]. As central regulators of cellular energy metabolism and apoptosis, dysfunctional mitochondria manifest through loss of membrane potential, impaired ATP production, excessive reactive oxygen species (ROS) generation, and the opening of the mitochondrial permeability transition pore (mPTP), ultimately culminating in neuronal damage or death [13, 14]. Excessive ROS not only triggers oxidative stress and inflammatory cascades but also exacerbates mitochondrial damage, creating a self‐perpetuating pathological cycle [8]. Additionally, disturbances in mitochondrial dynamics—particularly Drp1‐mediated hyperfission—have been implicated in ICH‐induced neuronal injury [15]. Inhibiting mitochondrial fission or reducing ROS production has shown therapeutic promise in mitigating ICH‐related neurotoxicity, suggesting that mitochondrial dynamics and redox pathways represent viable intervention targets [8, 16]. Nonetheless, monotherapies targeting single mechanisms often yield limited efficacy, and comprehensive strategies addressing multiple facets of mitochondrial dysfunction remain underexplored [8, 13, 17].

To achieve synergistic multi‐target intervention, it is essential to identify key molecular hubs that connect oxidative stress with mitochondrial destabilization, which remains a critical knowledge gap in current research. Recently, a series of studies have focused on the functional role of voltage‐dependent anion channel 1 (VDAC1), a key transmembrane protein located in the outer mitochondrial membrane. These studies have proposed that excessive activation of VDAC1 not only affects the permeability of cellular metabolites and ions but also induces mitochondrial permeability transition pore (mPTP) opening, leading to mitochondrial membrane potential collapse and intracellular release of pro‐apoptotic factors, thereby exacerbating neuronal injury [17, 18, 19, 20]. In parallel, Lysophosphatidic Acid Receptor 1 (LPAR1) has been identified as a central modulator of post‐ICH inflammatory responses and mitochondrial signaling. LPAR1 influences VDAC1 expression and conformational dynamics [21, 22]. These findings suggest that the LPAR1/VDAC1 signaling axis may represent a fundamental molecular pathway driving mitochondrial instability and neuronal damage in ICH [23, 24]. Although several pharmacological studies have shown that blocking VDAC1 or LPAR1 alone can ameliorate neurological injury, the underlying mechanisms have not been fully elucidated [18, 25]. Based on this evidence, we hypothesize that the LPAR1‐VDAC1 axis serves as a central molecular hub mediating ROS accumulation‐induced mitochondrial collapse.

Among various mitochondrial‐protective compounds, the VDAC1 inhibitor 4,4'‐Diisothiocyanatostilbene‐2,2'‐disulfonic Acid (DIDS) and the Mitochondrial Division Inhibitor 1 (Mdivi‐1) have garnered significant attention [26, 27]. DIDS suppresses VDAC1 activation. Mdivi‐1 inhibits the activity of dynamin‐related protein 1 (Drp1), attenuating excessive mitochondrial fission and restoring mitochondrial function and metabolic balance [26, 28]. Although both agents show promising efficacy [8, 29], conventional co‐administration strategies face significant translational hurdles, including poor targeting, limited BBB permeability, and systemic toxicity [30, 31]. Therefore, developing a delivery system that enables precise, efficient, and safe co‐delivery of these drugs to lesion sites is a critical unmet need. Recently, nanoparticle platforms have emerged as a promising strategy to overcome these limitations, offering enhanced targeting capabilities and controlled drug release profiles under pathological conditions [32].

Biomimetic membrane‐coated stimulus‐responsive intelligent nanodelivery systems have been shown to significantly enhance drug penetration across the BBB and enable precise drug release within lesion areas [33, 34]. In this study, we developed a novel nanoplatform by integrating mouse brain microvascular endothelial cell membranes with a nanoplatform exhibiting H_2_O_2_‐dependent drug‐release behavior. This system effectively co‐encapsulates the VDAC1 inhibitor DIDS and Mdivi‐1, enabling environment‐specific drug release within the lesion microenvironment of ICH.

Using in vitro neuronal models, scRNA‐seq, targeted metabolomic analysis, and in vivo imaging in animal models, we systematically investigated the molecular mechanisms by which this nanoplatform ameliorates mitochondrial dysfunction, attenuates neuronal apoptosis, and mitigates cognitive impairment following ICH, primarily through modulation of the LPAR1/VDAC1 axis. Furthermore, gene silencing strategies targeting LPAR1 were employed to validate the target specificity and mechanistic precision of the intervention. We anticipate that this study will not only fill the current knowledge gap regarding the mechanistic role of the LPAR1‐VDAC1 pathway in secondary injury after ICH, but also provide a novel multi‐target nanotherapeutic strategy with substantial potential for clinical translation in ICH treatment.

## 2 Materials and Methods

### 2.1 Ethical Approval and Experimental Design

Male C57BL/6J mice (22–25 g), obtained from Beijing Vital River Laboratory Animal Technology Co., Ltd. (Beijing, China), were assigned to two experimental cohorts. The standard treatment cohort comprised six groups (n = 15 per group): Sham, ICH+NS, ICH+DIDS, ICH+Mdivi‐1, ICH+Free DIDS+Mdivi‐1, and ICH+NP@D+M. A separate LPAR1 knockdown validation cohort comprised two groups: ICH+NP@D+M+siRNA NC and ICH+NP@D+M+siLPAR1. For the endpoint analyses, six mice per group were analyzed. Group allocation was performed using a randomized number table, ensuring comparability in age and body weight across groups. All animal procedures were conducted in strict accordance with the protocol approved by the Affiliated Haikou Hospital, Xiangya School of Medicine, Central South University Institutional Animal Ethics Committee (Approval No. 2024067) and adhered to the internationally recognized 3Rs principles (Replacement, Reduction, and Refinement), with efforts made to minimize both the number of animals used and their suffering. Primary cortical neurons were isolated from neonatal C57BL/6J mice obtained from the same facility. All in vitro experiments were performed in at least three independent biological replicates to ensure the robustness and reproducibility of the results.

### 2.2 Preparation of DIDS/Mdivi‐1‐Loaded Polymeric Nanoparticle Cores

Drug‐loaded polymeric nanoparticle cores were prepared using PLGA‐PEG‐PLGA copolymers as the primary carrier via a classical solvent evaporation method. Briefly, PLGA‐PEG‐PLGA (CAS: 162222‐68‐6, Cat. No.: 764833, Sigma‐Aldrich, St. Louis, MO, USA) was dissolved in dichloromethane (DCM) to yield a 10 mg/mL organic solution. Separately, the therapeutic agents DIDS (CAS: 67483‐13‐0, Cat. No.: D3514, Sigma‐Aldrich, St. Louis, MO, USA) and Mdivi‐1 (CAS: 338967‐87‐6, Cat. No.: HY‐15886, MedChemExpress, Monmouth Junction, NJ, USA) were dissolved in Dimethyl Sulfoxide (DMSO) at a concentration of 5 mg/mL. These drug solutions were mixed with the polymer solution at a drug‐to‐polymer weight ratio of 1:10, followed by thorough homogenization. The resulting mixture was added dropwise (0.2 mL/min) into an aqueous phase containing 1% polyvinyl alcohol (PVA) under ice‐bath conditions and subjected to ultrasonication (100 W, 30% amplitude, 10 min) to form uniform nanoparticles. The organic solvent was removed by magnetic stirring at 500 rpm for 3–4 h at room temperature in a fume hood. Nanoparticles were collected by centrifugation (12 000 rpm, 20 min, 4°C), washed repeatedly with ultrapure water to remove residual solvent and unencapsulated drug, and lyophilized after final purification. Their H_2_O_2_‐dependent drug‐release behavior was subsequently evaluated in vitro at different H_2_O_2_ concentrations. The amounts of unencapsulated or lost DIDS and Mdivi‐1 in the supernatant/wash fractions after nanoparticle‐core purification and membrane coating were quantified by LC‐MS/MS, and the drug mass retained in the final purified NP@D+M formulation was calculated for each batch by mass balance. EE was calculated as the retained mass of each drug in the final purified formulation divided by its corresponding initial input mass, and DL as the retained drug mass divided by the total dry mass of the final purified NP@D+M formulation. Six independently prepared formulation batches were analyzed. In addition, drug release profiles under varying H_2_O_2_ concentrations were assessed to evaluate the H_2_O_2_‐dependent release behavior of the system. Finally, the morphology, size distribution, and dispersion of the nanoparticles were characterized by transmission electron microscopy (TEM; JEOL JEM‐2100). For TEM analysis, samples were prepared by placing a droplet of nanoparticle suspension onto a carbon‐coated copper grid, followed by drying at room temperature (Figure S1).

### 2.3 LC‐MS/MS Metabolomic Analysis

LC‐MS/MS analysis was performed on an AB Sciex QTRAP 5500 mass spectrometer equipped with an electrospray ionization source (ESI). Multiple reaction monitoring (MRM) was conducted in rapid positive/negative ion‐switching mode. To ensure stable ionization of key metabolites involved in glycolysis and the TCA cycle, the main ion source parameters were set as follows: ion spray voltage (IS), 5500 V in positive ion mode and −4500 V in negative ion mode; source temperature (TEM), 500°C; curtain gas (CUR), 35 psi; ion source gas 1 (GS1) and ion source gas 2 (GS2), both 50 psi; and collision gas (CAD), medium.

To ensure quantitative accuracy and data reproducibility, external calibration combined with internal standard normalization was used for quantitative analysis. A mixed standard stock solution containing all target metabolite standards was serially diluted with 50% aqueous methanol to generate calibration curves ranging from 1 to 5000 ng/mL. Linear regression analysis showed that the correlation coefficients (R^2^) of all targeted metabolites were greater than 0.99. The limits of detection (LOD) and limits of quantification (LOQ) were determined based on signal‐to‐noise ratios (S/N) of at least 3:1 and 10:1, respectively.

During sample preparation, isotope‐labeled compounds were added to the extraction system as internal standards to monitor matrix effects and extraction efficiency. The extraction recoveries were maintained within the range of 85%–115%. To monitor the operational stability of the LC‐MS/MS system, mixed standard quality control (QC) samples at the same concentration were randomly inserted into each analytical batch, with one QC sample included after every 10 test samples. The QC results showed that the intra‐batch and inter‐batch relative standard deviations (RSDs) of all target metabolites were less than 10%, indicating high precision and reliability of the LC‐MS/MS platform. Raw mass spectrometry data were processed using MultiQuant software (AB Sciex) for peak area integration and concentration calculation [35].

### 2.4 Construction of Biomimetic Membrane‐Coated Nanoparticles

Mouse brain microvascular endothelial cells (bEnd.3 cell line, obtained from ATCC) were thawed and seeded onto culture dishes pre‐coated with 0.1% gelatin. The cells were cultured in Dulbecco's Modified Eagle Medium (DMEM) (Gibco) supplemented with 10% fetal bovine serum (FBS) (Gibco), 100 U/mL penicillin, and 100 µg/mL streptomycin at 37°C in a humidified atmosphere containing 5% CO_2_. Once cell confluence exceeded 90%, they were harvested. After triple washing with PBS, cells were scraped on ice and centrifuged at 1500 rpm for 10 min at 4°C to remove residual medium and debris. The resulting cell pellet was resuspended in a hypotonic lysis buffer (20 mM Tris‐HCl, 10 mM KCl, 2 mM MgCl_2_, pH 7.4) supplemented with protease inhibitors, and incubated on ice for 30 min. Cell membranes were disrupted by 20 gentle strokes using a Dounce homogenizer on ice. The homogenate was subjected to differential centrifugation: first at 3000 rpm for 10 min to remove nuclei and unbroken cells, then at 12 000 rpm for 30 min to isolate the membrane fraction. The membrane pellet was washed twice in PBS and resuspended. Protein concentration was quantified using a Bicinchoninic Acid (BCA) assay (Thermo Fisher), adjusted to 1 mg/mL, and stored at –80°C for future use.

The membrane coating of nanoparticles was achieved using a sonication‐assisted extrusion method. Equal masses of the extracted cell membrane suspension and ROS‐responsive PLGA‐PEG@D/M nanoparticles were mixed at a 1:1 mass ratio (membrane protein weight to nanoparticle weight, w/w). The mixture was then subjected to sonication (Scientz‐IID, 100 W, 20% amplitude, 2 min total with 3‐s pulses and 2‐s intervals) on ice to facilitate membrane fusion. The resulting suspension was passed sequentially through 400, 200, and 100 nm polycarbonate membranes using a handheld extruder (Avanti Polar Lipids) for at least 10 cycles to ensure uniform membrane coating. The final biomimetic membrane‐coated nanoparticles (NP@D+M) were purified by ultracentrifugation (15 000 rpm, 30 min, 4 °C) to remove excess unbound membrane fragments, resuspended in PBS, and stored at 4°C until use (Figure S2). To further verify the membrane‐coating efficiency and thoroughly separate any residual unbound bare nanoparticles, discontinuous sucrose density gradient centrifugation was performed during the preparation process, which had been omitted from the previous manuscript description. Briefly, sucrose solutions with mass fractions of 20%, 30%, 40%, 50%, and 60% (w/v) were prepared using sucrose (Cat. No.: S0389, Sigma‐Aldrich, St. Louis, MO, USA) and sequentially layered into ultracentrifuge tubes from low to high concentration to form a discontinuous gradient. The preliminarily purified nanoparticle suspension was carefully loaded onto the top of the gradient and centrifuged at 100 000 × g for 2 h at 4°C using an ultracentrifuge (Optima L‐100 XP, Beckman Coulter, Brea, CA, USA). After centrifugation, bands at specific density gradient interfaces were separately collected, diluted with PBS, and washed before subsequent physicochemical characterization and functional experiments [36].

To evaluate the integrity and morphology of the membrane coating, TEM (JEM‐2100, JEOL) was employed. Western Blot (WB) was performed to verify the presence of specific endothelial membrane proteins—such as CD31 (ab182981, 1:1000, Abcam), ZO‐1 (ab221547, 1:1000, Abcam), and VE‐cadherin (ab33168, 1:1000, Abcam)—on the nanoparticle surface, confirming successful membrane transfer. Additionally, sodium dodecyl sulfate‐polyacrylamide gel electrophoresis (SDS‐PAGE) followed by Coomassie brilliant blue staining was used to characterize the composition and retention of membrane proteins on the coated nanoparticles.

### 2.5 Characterization of the Physicochemical Properties of the Nanoplatform

The physicochemical characteristics of the fabricated nanoplatform were first assessed using dynamic light scattering (DLS; Malvern Zetasizer Nano ZS) to determine particle size and zeta potential. Nanoparticle suspensions were diluted to approximately 0.2 mg/mL and measured at 25°C, with each sample analyzed in triplicate. The average hydrodynamic diameter, polydispersity index (PDI), and zeta potential were recorded to evaluate size uniformity and surface charge distribution. TEM (JEM‐2100, JEOL, Japan) was subsequently employed to examine particle morphology. Nanoparticle suspensions were deposited onto carbon‐coated copper grids, air‐dried, and imaged at an accelerating voltage of 80 kV to visualize surface structure and membrane encapsulation.

To confirm successful DL, UV–vis spectrophotometry (Thermo Fisher Evolution 220) was used to identify the characteristic absorption peaks of DIDS and Mdivi‐1, noting their maximal wavelengths and peak areas. The specificity of membrane coating was further validated using Raman spectroscopy (Thermo Fisher DXRxi). Dried samples were placed on glass slides, and spectra were collected across the 400–4000 cm^−^^1^ range. Raman profiles of membrane‐coated nanoparticles were compared with those of bare nanoparticles and pure cell membranes to verify the retention of characteristic peaks corresponding to membrane components such as proteins and lipids.

Drug EE and loading capacity (DL%) were quantified independently for each final purified formulation by LC‐MS/MS (AB Sciex QTRAP 5500). Freeze‐dried nanoparticles from each preparation were dissolved in methanol, sonicated for 30 min, and centrifuged at 15 000 rpm for 10 min. The supernatants were analyzed to determine total drug content. EE% was calculated as the percentage of the initially fed drug recovered in the final nanoparticle formulation, and DL% was calculated as the drug mass relative to the total mass of the corresponding purified formulation, based on standard curves. To assess the platform's release behavior under oxidative conditions, drug‐loaded nanoparticles were incubated in PBS (pH 7.4) containing various concentrations of H_2_O_2_ (0, 100, and 500 µM) at 37°C under gentle agitation. At predefined time points (0, 2, 4, 8, 12, 24, and 48 h), aliquots were collected. Nanoparticles were first pelleted by centrifugation at 12 000 rpm for 20 min, and the resulting supernatant was further filtered through a 0.22 µm membrane to remove any residual large particles or aggregates. A second filtration step using a smaller‐pore membrane (0.1 µm) was then performed for validation to ensure that nanoparticles did not pass through the filter and interfere with the measurement. The released drug content was subsequently quantified by LC‐MS/MS, and cumulative release curves were plotted to evaluate the H_2_O_2_‐dependent release behavior.

To evaluate the stability of the nanoparticles in complex physiological environments, the prepared nanoplatforms were incubated in PBS containing 10% FBS or artificial cerebrospinal fluid (aCSF, Cat. No. 5726, Tocris Bioscience, Bristol, UK) at 37°C. DLS was used to monitor the hydrodynamic diameter and PDI of the nanoparticles at predefined time points (0, 12, 24, 48, and 72 h), thereby assessing their colloidal stability. In addition, to visually characterize their oxidative stress‐responsive properties, nanoparticles were exposed to a solution containing 500 µM H_2_O_2_ for 4 h. The ultrastructural changes were then observed by transmission electron microscopy (TEM) and compared with those in the untreated group (0 µM H_2_O_2_) for morphological analysis [37].

### 2.6 Primary Cortical Neuron Culture and In Vitro ICH Model Establishment

Neonatal C57BL/6J mice (postnatal day 0–1) were euthanized via decapitation under sterile conditions. Bilateral cerebral cortices were rapidly dissected and transferred into ice‐cold Hank's Balanced Salt Solution (HBSS; Gibco), followed by careful removal of the meninges and blood vessels. The cortical tissue was then minced into ∼1 mm^3^ fragments and incubated in 0.25% trypsin (Gibco) at 37°C for 15 min, with gentle agitation at intervals to facilitate enzymatic dissociation. The digestion was promptly quenched by adding DMEM containing 10% FBS, and the cell suspension was passed through a 200 µm filter to remove debris. Following centrifugation at 800 rpm for 5 min, the cell pellet was resuspended in Neurobasal medium supplemented with B27 and 2 mM GlutaMAX (Gibco), and plated onto 6‐well plates or glass coverslips pre‐coated with 10 µg/mL poly‐D‐lysine (Sigma–Aldrich). Cultures were maintained at 37°C in a humidified atmosphere with 5% CO_2_, with half‐medium changes every 3 days. Neurons were allowed to differentiate for 7 days before subsequent experiments.

To establish an in vitro model simulating secondary neurotoxicity following ICH, mature primary neurons were preconditioned with serum‐free medium for 2 h, followed by exposure to 100 ng/mL lipopolysaccharide (LPS; CAS: 297‐473‐0, Cat. No.: L2630, Sigma–Aldrich, St. Louis, MO, USA) and 10 µM hemoglobin (Hb; CAS: 9008‐02‐0, Cat. No.: H7379, Sigma–Aldrich, St. Louis, MO, USA) for 24 h to induce neuroinflammatory and oxidative stress responses. After stimulation, the treatment solution was carefully aspirated, and cells were gently washed twice with PBS. Fresh culture medium containing different experimental interventions (e.g., blank control, nanoparticle formulation, or free drugs) was then added for downstream assays. Successful model establishment was confirmed by morphological changes (e.g., dendritic retraction and somatic shrinkage), reduced cell viability (∼30% decrease, assessed via Cell Counting Kit‐8 [CCK‐8] assay), and markedly increased ROS levels as indicated by 2',7'‐Dichlorodihydrofluorescein Diacetate (DCFH‐DA) fluorescence staining—collectively indicating reliable oxidative and cytotoxic stress consistent with ICH‐related injury.

Standardized Quantification of Live/Dead Staining: To ensure the accuracy of the experimental results, primary neurons were seeded at an initial density of 1 × 10^5^ cells/cm^2^ to maintain consistent starting conditions across all groups. During image acquisition, five independent fields of view were randomly selected from each sample using a confocal microscope. To correct for the influence of cell detachment induced by LPS/Hb on the total cell number, the live cell ratio was calculated using the following formula: Live cell ratio (%) = (number of Calcein‐AM‐positive cells / total number of DAPI‐positive cells) × 100%. All cell counting procedures were performed using ImageJ software (v1.53, NIH) by investigators blinded to the group allocation.

### 2.7 SDS‐PAGE and Western Blot Characterization

To evaluate the retention of functional membrane proteins, three types of samples were prepared: source endothelial cell lysates (Cell), purified blank cell membranes (Membrane), and biomimetic nanoparticles (NP@D+M). For the source cell group, logarithmically growing cells were collected and lysed on ice for 30 min using RIPA strong lysis buffer (Cat# P0013B, Beyotime, China) containing 1% protease inhibitor PMSF (Cat# ST506, Beyotime, China), followed by centrifugation to collect the supernatant. For the blank cell membrane group, purified cell membrane fragments were extracted using a membrane protein extraction kit (Cat# 89842, Thermo Fisher Scientific, USA) according to the manufacturer's instructions, based on differential centrifugation. For the NP@D+M group, the prepared nanoparticle suspension was ultracentrifuged at 100 000 × *g* for 30 min at 4°C to collect the precipitate, which was then resuspended in RIPA lysis buffer and disrupted by ultrasonication.

Protein concentrations in all groups were normalized using a BCA protein assay kit (Cat# 23225, Thermo Fisher Scientific, USA). Equal amounts of total protein (20 µg) were mixed with 5× SDS loading buffer (Cat# P0015, Beyotime, China) and boiled at 95°C for 10 min for denaturation. The samples were then loaded onto 10% SDS‐PAGE gels (Cat# PG112, Epizyme, China) for electrophoresis. For total protein profiling, the gels were removed and incubated with Coomassie Brilliant Blue R‐250 staining solution (Cat# P0017, Beyotime, China) on a decolorizing shaker at room temperature for 2 h. The gels were subsequently destained until the background became clear and then imaged.

For Western blot analysis of specific key proteins, proteins in the gel were transferred onto 0.45 µm PVDF membranes (Cat# IPVH00010, Millipore, USA) under constant current. After blocking with 5% BSA at room temperature for 1 h, the membranes were incubated overnight at 4°C with primary antibodies against CD31 (1:1000, Cat# ab28364, Abcam, UK), ZO‐1 (1:1000, Cat# ab216880, Abcam, UK), and VE‐cadherin (1:1000, Cat# ab33168, Abcam, UK). The next day, the membranes were washed three times with TBST and incubated with HRP‐conjugated goat anti‐rabbit/mouse IgG secondary antibody (1:5000, Cat# ab6721, Abcam, UK) at room temperature for 1 h. Finally, chemiluminescent signals were developed using an enhanced ECL substrate (Cat# 32106, Thermo Fisher Scientific, USA). Band intensities were quantified using ImageJ software, with GAPDH or a specific internal reference used for normalization.

### 2.8 Purity Identification of Primary Cortical Neurons

To verify the purity of primary neuronal cultures, immunofluorescence staining was performed on cells cultured for 7 days. After washing with PBS, cells were fixed with 4% paraformaldehyde for 15 min, permeabilized with 0.3% Triton X‐100 for 10 min, and blocked with 5% BSA at room temperature for 1 h. The cells were then incubated overnight at 4°C with a primary antibody mixture containing rabbit anti‐NeuN antibody (ab177487, 1:500, Abcam) and mouse anti‐GFAP antibody (ab10062, 1:1000, Abcam). After three washes with PBS the next day, the cells were incubated with Alexa Fluor 488‐conjugated goat anti‐rabbit IgG (1:500) and Alexa Fluor 594‐conjugated goat anti‐mouse IgG (1:500) at room temperature in the dark for 1 h. Finally, the cells were mounted with an antifade mounting medium containing DAPI. Five random fields were captured using a confocal microscope (Olympus FV3000), and the numbers of NeuN‐positive, GFAP‐positive, and total DAPI‐positive cells were quantified using ImageJ software to calculate the purity percentage [38].

### 2.9 Microtubule‐Associated Protein 2 (MAP2) Immunofluorescence Staining and Neurite Integrity Analysis

To evaluate the protective effect of the nanoplatform against LPS/Hb‐induced neuronal structural injury, MAP2 immunofluorescence staining was performed to assess neurite integrity. After treatment in different groups, including the blank control, ICH + NS, free drug, and NP@D+M groups, primary cortical neurons were gently washed twice with pre‐cooled PBS and fixed with 4% paraformaldehyde at room temperature for 15 min. After fixation, the cells were washed three times with PBS, permeabilized with 0.3% Triton X‐100 for 15 min, and blocked with 5% BSA at room temperature for 1 h to prevent nonspecific binding. After removing the blocking solution, the cells were incubated overnight at 4°C in a humidified chamber with a specific primary antibody against MAP2, rabbit anti‐MAP2 antibody (ab32454, 1:500, Abcam). The next day, after three washes with PBS, the cells were incubated with Alexa Fluor 488‐conjugated goat anti‐rabbit IgG (1:500, Thermo Fisher) at room temperature in the dark for 1 h. Finally, the cells were mounted with an antifade mounting medium containing DAPI. Z‐stack images were acquired from five randomly selected independent fields using a confocal laser scanning microscope (Olympus FV3000). Image analysis was performed using ImageJ software (NIH) combined with the NeuronJ plugin. The total neurite length of MAP2‐positive neurons in each field was manually traced and measured, and the average neurite length in each group was calculated to quantitatively evaluate dendritic integrity. All data were analyzed by investigators blinded to the group allocation [39].

### 2.10 Lactate Dehydrogenase (LDH) Release Assay

To evaluate neuronal membrane damage and necrosis in the in vitro model, LDH release was quantified using an LDH cytotoxicity assay kit (Beyotime Biotechnology, China) according to the manufacturer's instructions. Briefly, after primary neurons in each group were subjected to the indicated drug treatments for 24 h, the 96‐well plate was centrifuged at 400 × *g* for 5 min to pellet suspended cells and debris. Subsequently, 120 µL of cell culture supernatant was carefully collected from each well and transferred to a new clean 96‐well plate. Then, 60 µL of prepared LDH detection working solution was added to each well, followed by incubation for 30 min at room temperature with gentle shaking in the dark. After incubation, the absorbance was measured at 490 nm using a multifunctional microplate reader (Bio‐Tek, USA), with 600 nm set as the reference wavelength for dual‐wavelength correction. LDH release levels were finally normalized and expressed as percentages relative to the normal control group (Control) [40].

### 2.11 Targeted Uptake of the Nanoplatform and Confocal Imaging Analysis

The biomimetic nanoplatform with H_2_O_2_‐dependent drug‐release behavior was fluorescently labeled at room temperature using 1,1'‐dioctadecyl‐3,3,3',3'‐tetramethylindocarbocyanine iodide (DiI, Thermo Fisher). DiI was dissolved in ethanol to a final concentration of 5 µM and thoroughly mixed with the nanoparticle suspension. The mixture was incubated in the dark for 30 min at room temperature to allow the dye to intercalate into the lipid bilayer of the nanoparticles. Following incubation, the labeled nanoparticles were washed three times by ultracentrifugation (15 000 rpm, 30 min, 4°C) to remove any unincorporated DiI. Labeling efficiency was indirectly quantified by measuring the residual fluorescence in the supernatant using a spectrophotometer (Thermo Fisher Evolution 220) and was confirmed to exceed 90%.

For uptake studies, primary cortical neurons were pre‐treated with LPS and Hb for 24 h to induce an inflammatory oxidative stress environment. The culture medium was then replaced with fresh serum‐free medium, and cells were randomly assigned to one of three groups: untreated control, uncoated nanoparticles, or biomimetic membrane‐coated nanoparticles. All groups received DiI‐labeled nanoparticles at an equivalent concentration (100 µg/mL) and were incubated at 37°C for 4 h. After incubation, cells were gently washed three times with PBS to remove uninternalized nanoparticles. Nuclear staining was performed by incubating the cells for 10 min with Hoechst 33342 (5 µg/mL, Thermo Fisher) in serum‐free medium, followed by an additional PBS wash. Fluorescence imaging was conducted using a confocal laser scanning microscope (Olympus FV3000), with excitation/emission wavelengths of 561 nm for DiI and 405 nm for Hoechst. Z‐stack images were acquired from five randomly selected fields per group to qualitatively assess intracellular fluorescence distribution. Quantification of nanoparticle uptake was performed using ImageJ software by measuring the average red fluorescence intensity per cell, thereby evaluating the nanoplatform's targeting efficiency under oxidative stress conditions.

### 2.12 Mitochondrial Functional Assessment

ΔΨm was assessed using 5,5',6,6'‐Tetrachloro‐1,1',3,3'‐tetraethylbenzimidazolylcarbocyanine iodide (JC‐1) (CAS: 3520‐43‐2, Cat. No.: T3168, Thermo Fisher Scientific, Waltham, MA, USA). After treatment, neurons were gently washed twice with PBS and incubated in serum‐free medium containing 2 µM JC‐1 at 37°C for 20 min in the dark. Following incubation, cells were washed three times with PBS, replaced with fresh medium, and immediately imaged under a fluorescence microscope (Olympus IX73). Red fluorescence (Ex/Em = 525/590 nm) corresponds to JC‐1 aggregates indicating intact ΔΨm, while green fluorescence (Ex/Em = 490/530 nm) reflects JC‐1 monomers indicative of depolarization. Five random fields per group were captured, and the red‐to‐green fluorescence ratio was quantified using ImageJ to assess membrane potential changes.

Mitochondrial ROS production was detected using MitoSOX Red (Cat. No.: M36008, Thermo Fisher Scientific, Waltham, MA, USA). First, in situ fluorescence images were acquired using a confocal laser scanning microscope (Olympus FV3000) in the red channel (Ex/Em = 510/580 nm). Quantitative analysis was then performed using ImageJ software (v1.53). Five independent fields were randomly selected from each group, and the integrated density and area of the red fluorescence signal were measured. The mean fluorescence intensity (MFI) was calculated as integrated density/total area, followed by subtraction of the background fluorescence from cell‐free regions to exclude nonspecific signals. In addition, cells were incubated with 5 µM MitoSOX working solution in each well at 37°C for 15 min in the dark and then washed twice with PBS. MitoSOX fluorescence was immediately detected by flow cytometry using a BD FACSCanto II system in the red fluorescence channel (Ex/Em = 510/580 nm). Flow cytometry data were analyzed using FlowJo software (v10.8, BD Biosciences) to quantify fluorescence intensity distributions and compare mitochondrial oxidative stress levels among groups.

Mitochondrial membrane integrity was examined using MitoTracker Green FM (Thermo Fisher). Neurons were prewashed with PBS and incubated with a 200 nM working solution at 37°C for 30 min. After gentle washing, cells were imaged directly. Fluorescence signals (Ex/Em = 490/516 nm) were analyzed to determine mitochondrial morphology and network distribution, serving as indicators of membrane structural integrity.

Intracellular ATP levels were measured using the CellTiter‐Glo Luminescent Cell Viability Assay (Promega). After removing the culture medium, plates were equilibrated to room temperature for 30 min. Equal volumes of CellTiter‐Glo reagent were added to each well, followed by vigorous shaking and incubation for 10 min to ensure complete cell lysis. Luminescence was measured using a multimode microplate reader (BioTek Synergy H1), and ATP concentrations were calculated from a standard curve and expressed as nmol/mg protein. All experiments were conducted in triplicate to ensure reproducibility and accuracy.

### 2.13 Quantitative Scoring of Mitochondrial Ultrastructure (Modified Flameng Scoring System)

Quantitative analysis of transmission electron microscopy images was performed using a modified Flameng scoring system. The assessment was independently conducted by two observers who were blinded to the group allocation. The scoring criteria were as follows: 0 points, normal mitochondrial structure with tightly arranged cristae and homogeneous matrix electron density; 1 point, mild mitochondrial swelling, slight widening of the inter‐cristae space (<20%), and mildly reduced matrix density; 2 points, moderate mitochondrial swelling, partial cristae disruption or dissolution (20%–50%), and obvious matrix vacuolization; 3 points, severe mitochondrial swelling, extensive cristae loss (>50%), severe matrix vacuolization, and impaired outer membrane integrity; and 4 points, extreme mitochondrial swelling and rupture, complete disappearance of cristae, and leakage of mitochondrial contents. At least 50 mitochondria were randomly selected from each group, derived from 3–5 independent fields or animals, for scoring. The mean damage index (MDI) was then calculated to quantify mitochondrial ultrastructural injury [41].

### 2.14 Analysis of Apoptosis Levels

Apoptotic cell populations were initially assessed using an Annexin V‐FITC/PI dual‐staining kit (BD Biosciences). Following treatment, neurons were gently washed twice with PBS and dissociated using 0.25% trypsin without EDTA. The resulting cell suspension was collected by gentle pipetting and centrifuged at 1200 rpm for 5 min at 4°C. After discarding the supernatant, cells were resuspended in 100 µL of 1× binding buffer. Each sample was incubated with 5 µL Annexin V‐FITC and 5 µL propidium iodide (PI) in the dark at room temperature for 15 min. Subsequently, 400 µL of binding buffer was added, gently mixed, and the samples were immediately analyzed using a BD FACSCanto II flow cytometer. Fluorescence signals were collected using the FITC channel (Ex/Em = 488/530 nm) and PI channel (Ex/Em = 488/617 nm). A minimum of 10 000 events were recorded per sample, and cell populations were classified into early apoptosis (Annexin V^+^/PI^−^), late apoptosis (Annexin V^+^/PI^+^), necrosis (Annexin V^−^/PI^+^), and viable cells.

The expression levels of apoptosis‐related proteins were determined via WB. Following treatment, cells were lysed on ice for 30 min using Radio‐Immunoprecipitation Assay (RIPA) buffer (Beyotime) supplemented with protease inhibitors. Lysates were centrifuged at 12 000 rpm for 15 min at 4°C, and protein concentrations in the supernatant were quantified using the BCA assay (Thermo Fisher). Equal amounts of protein (30 µg per lane) were resolved by 10% SDS‐PAGE and transferred to Polyvinylidene Fluoride (PVDF) membranes (Millipore). Membranes were blocked in 5% non‐fat milk for 1 h, then incubated overnight at 4°C with primary antibodies against Bcl‐2 (ab182858, 1:1000, Abcam), Bax (ab32503, 1:1000, Abcam), cleaved caspase‐3 (#9661, 1:1000, Cell Signaling Technology), and β‐actin (ab8227, 1:5000, Abcam). The next day, membranes were washed three times with TBST (10 min each), incubated with Horseradish Peroxidase (HRP)‐conjugated secondary antibodies (1:5000, Cell Signaling Technology) for 1 h at room temperature, and developed using an enhanced chemiluminescence (ECL) system (Thermo Fisher). Bands were visualized using a ChemiDoc imaging system (Bio‐Rad), and densitometric analysis was performed using ImageJ software. Protein expression levels were normalized to β‐actin. All experiments were independently replicated three times to ensure reproducibility.

### 2.15 siRNA Knockdown Assay

Gene silencing of LPAR1 and VDAC1 was performed using target‐specific siRNAs purchased from Thermo Fisher. Primary cortical neurons were seeded into 6‐well plates and cultured until ∼70% confluence. Transfection was carried out using Lipofectamine RNAiMAX (Thermo Fisher) following the manufacturer's instructions. siRNAs were administered at a final concentration of 50 nM per well, with experimental groups including siLPAR1, siVDAC1, and siControl (negative control). After a 6‐h transfection period, the medium was replaced with fresh neuronal culture medium, and cells were incubated for an additional 48 h. Knockdown efficiency was validated at both the transcript and protein levels via qPCR and WB, respectively. For qPCR, total RNA was extracted using the TRIzol method (Thermo Fisher), and RNA purity was assessed with a NanoDrop spectrophotometer. cDNA was synthesized using the PrimeScript RT reagent kit (Takara), and quantitative PCR was performed with SYBR Green Master Mix (Takara). Primers were synthesized by Sangon Biotech. Cycling conditions were as follows: initial denaturation at 95°C for 30 s, followed by 40 cycles of 95°C for 5 s and 60°C for 30 s. Each sample was run in triplicate, and relative expression was calculated using the 2^–ΔΔCt^ method with GAPDH as the internal control.

For WB, protein extraction, SDS‐PAGE, membrane transfer, and detection were performed as previously described. LPAR1 (ab23698, 1:1000, Abcam) and VDAC1 (#4661, 1:1000, Cell Signaling Technology) protein levels were assessed using primary antibodies, with β‐actin (1:5000) serving as the loading control. Following confirmation of gene silencing, cells were subjected to various treatments under identical culture conditions: blank control, free DIDS/Mdivi‐1, and the NP@D+M nanoparticle platform. After 24 h of incubation, cells were harvested for functional assays, including JC‐1, mitochondrial ROS production (MitoSOX), ATP quantification (CellTiter‐Glo), and apoptosis analysis (Annexin V/PI staining). These assays were used to assess the impact of LPAR1 or VDAC1 knockdown on the therapeutic effects of the nanoparticle system and to elucidate the functional role of the LPAR1/VDAC1 axis in regulating mitochondrial homeostasis and neuronal apoptosis. All experiments were independently repeated three times to ensure reproducibility.

### 2.16 Construction and Transfection of the LPAR1 Overexpression Plasmid

The mouse LPAR1 overexpression plasmid (oeLPAR1; Cyagen Biosciences, Guangzhou, China) and the corresponding negative control plasmid (pNC) were constructed by Cyagen Biosciences. Transfection was performed using Lipofectamine 3000 reagent (L3000015, Invitrogen, Carlsbad, CA, USA) strictly according to the manufacturer's instructions. Primary neurons were seeded in 6‐well plates, and the final plasmid concentration during transfection was 2 µg/mL. Subsequent experiments were performed 48 h after transfection.

### 2.17 Co‐Immunoprecipitation (Co‐IP) and Protein Colocalization Analysis

Co‐IP assays were performed using the Thermo Fisher Pierce Classic IP Kit. Primary neurons subjected to different treatments were lysed on ice for 30 min using IP lysis buffer supplemented with protease inhibitors. Lysates were centrifuged at 12 000 rpm for 15 min at 4°C, and the supernatants were collected for protein quantification using the BCA assay (Thermo Fisher). For each reaction, 500 µg of total protein was first pre‐cleared with 20 µL of Protein A/G agarose for 1 h. After removal of the beads, the supernatant was incubated overnight at 4°C with 2 µg of anti‐LPAR1 antibody (Cell Signaling Technology). IgG isotype antibodies were used as negative controls. The following day, 40 µL of pre‐washed Protein A/G agarose beads were added and incubated for an additional 4 h at 4°C to capture the immune complexes. The precipitates were washed four times with wash buffer and resuspended in 2× SDS loading buffer, followed by boiling for 5 min. After centrifugation, the supernatant was subjected to WB analysis to detect VDAC1 (1:1000; Cell Signaling Technology). Co‐precipitated bands were used to confirm the protein–protein interaction. Input samples (5% of the total lysate) were simultaneously probed for LPAR1 and VDAC1 to verify initial protein expression levels.

Protein colocalization was assessed via immunofluorescence staining and confocal laser scanning microscopy (Olympus FV3000). Neurons cultured on coverslips were fixed with 4% paraformaldehyde for 15 min, washed with PBS, permeabilized with 0.3% Triton X‐100 for 10 min, and blocked with 5% bovine serum albumin (BSA) for 1 h. Primary antibodies against LPAR1 (1:200) and VDAC1 (1:200) were applied overnight at 4°C. After washing, cells were incubated with Alexa Fluor 488‐conjugated anti‐rabbit (A‐11008, 1:500, Thermo Fisher) and Alexa Fluor 594‐conjugated anti‐mouse secondary antibodies (A‐11012, 1:500; Thermo Fisher) for 1 h at room temperature in the dark. Nuclei were counterstained with 4',6‐diamidino‐2‐phenylindole (DAPI) (1 µg/mL) for 5 min. Coverslips were mounted using an anti‐fade reagent. Z‐stack images were acquired using a 63× oil‐immersion objective, and colocalization was quantified using the Pearson correlation coefficient calculated via the Coloc2 plugin in ImageJ. This analysis was used to evaluate the spatial interaction between LPAR1 and VDAC1 under ICH‐mimicking conditions.

### 2.18 Quantification of Key Metabolites in Glycolysis and the Tricarboxylic Acid (TCA) Cycle

Targeted metabolomic analysis was conducted using LC‐MS/MS (AB Sciex QTRAP 5500). Neurons from each experimental group were washed twice with PBS, and metabolites were extracted using cold methanol:water (80:20, v/v). Cells were scraped and pre‐cooled at −80°C for 15 min to stabilize metabolite profiles. Lysates were centrifuged at 14 000 rpm for 15 min at 4°C, and supernatants were transferred to fresh tubes and vacuum‐dried. Dried extracts were reconstituted in 100 µL of 50% methanol and sonicated for 5 min. After another centrifugation step, the supernatants were used for LC‐MS/MS analysis.

Separation was achieved using a Waters Acquity UPLC HSS T3 column (2.1 × 100 mm, 1.8 µm) with mobile phases consisting of 0.1% formic acid in water (A) and 0.1% formic acid in acetonitrile (B). The gradient elution profile was as follows: 0–2 min, 2% B; 2–6 min, linear increase to 30% B; 6–12 min, to 90% B; 12–14 min, held at 90% B; 14–14.1 min, returned to 2% B; equilibrated to 18 min. The flow rate was 0.3 mL/min, with a 5 µL injection volume. Data were acquired in both positive and negative ESI modes. Key metabolites—including lactate (m/z 89.0), pyruvate (m/z 87.0), citrate (m/z 191.0), α‐ketoglutarate (m/z 145.0), and succinate (m/z 117.0)—were quantified using multiple reaction monitoring (MRM) mode. Calibration curves were generated using authentic standards for each metabolite, all achieving R^2^ > 0.99.

Each sample was analyzed in triplicate, with blank and pooled QC samples randomly inserted into the batch sequence to ensure accuracy and reproducibility. Metabolite concentrations were normalized to protein content (nmol/mg protein), measured in parallel using the BCA assay (Thermo Fisher). Inter‐group comparisons of relative metabolite levels were conducted to evaluate the regulatory effects of the nanoparticle platform on glycolytic and TCA cycle metabolic pathways.

### 2.19 Single‐Cell Isolation and Single‐Cell RNA Sequencing (scRNA‐seq) Library Preparation

Following euthanasia, mouse brains were rapidly dissected, and the target brain regions were isolated. After a brief rinse with ice‐cold PBS, the tissue was cut into 1–2 mm^3^ fragments and incubated in prewarmed digestion buffer (Papain, 10–20 U/mL, supplemented with DNase I) at 37°C for 15–30 min, with gentle intermittent trituration to facilitate dissociation. The reaction was terminated by adding cold PBS containing BSA, and the resulting suspension was sequentially filtered through 70‐µm and 40‐µm meshes to obtain a single‐cell suspension. The suspension was passed through a 40 µm cell strainer to remove aggregates and centrifuged at 300 g for 5 min at 4°C. After discarding the supernatant, cells were resuspended in 1× PBS supplemented with 0.04% BSA and adjusted to a final concentration of approximately 1000 cells/µL. Cell viability, assessed via trypan blue exclusion, exceeded 90%, ensuring suitability for downstream library construction. Single‐cell capture and library preparation were performed using the 10x Genomics Chromium Next GEM Single Cell 3' v3.1 Reagent Kit, following the manufacturer's protocol. Single cells were encapsulated into oil droplets for barcoding and reverse transcription. The resulting cDNA underwent 12–14 cycles of PCR amplification. Library construction included cDNA fragmentation, end repair, A‐tailing, adaptor ligation, and index amplification.

Library quality was assessed using the Agilent Bioanalyzer 2100, and concentrations were quantified with the Qubit Fluorometer (Thermo Fisher). Qualified libraries were sequenced on the Illumina NovaSeq 6000 platform in paired‐end 150 bp (PE150) mode, targeting a minimum depth of 50 000 reads per cell. Raw sequencing data were processed using Cell Ranger (v6.1.1), including barcode demultiplexing, unique molecular identifier (UMI) correction, and alignment to the mouse reference genome (mm10) to generate gene expression matrices. Cells with fewer than 200 detected genes or mitochondrial gene content exceeding 15% were excluded. Downstream analyses were performed in R (v4.1.2) using the Seurat package (v4.0.3), including normalization, principal component analysis (PCA), t‐distributed stochastic neighbor embedding (t‐SNE), uniform manifold approximation and projection (UMAP), clustering (FindClusters), and identification of differentially expressed genes (DEGs) using thresholds of logFC > 0.25 and *p* < 0.05. These analyses were used to investigate transcriptional heterogeneity among neuronal subpopulations under ICH conditions and their responses to nanoparticle‐based interventions.

### 2.20 Metabolomic Sequencing

Following drug administration, mouse brain tissues were briefly rinsed with PBS and immediately quenched with pre‐chilled 80% methanol containing internal standards to arrest metabolism and precipitate proteins. Samples were subjected to short bursts of low‐temperature ultrasonication, centrifuged, and the supernatants were collected. After concentration and drying, metabolites were reconstituted in an ammonium salt–based water/acetonitrile solution. All steps were performed under cold conditions to preserve labile metabolites such as sugar phosphates and organic acids. Metabolite profiling was conducted using a hydrophilic interaction chromatography (HILIC)‐based UHPLC–high‐resolution mass spectrometry system (UHPLC‐Q Exactive). The mobile phase consisted of acetonitrile and a weakly alkaline ammonium acetate aqueous solution. Data were acquired primarily in negative ion electrospray mode, integrating full‐scan and data‐dependent MS/MS acquisition. Targeted scans were applied to key metabolites—including glucose‐6‐phosphate, fructose‐6‐phosphate, pyruvate, lactate, citrate, α‐ketoglutarate, succinate, and malate—to enhance sensitivity. Quality control (QC) was maintained by inserting one pooled QC sample every ten test samples and including blanks to monitor system stability and background noise. Data processing involved peak extraction, retention time alignment, blank subtraction, internal standard normalization, and batch‐effect correction. Metabolite identification was based on accurate mass, retention time, and MS/MS spectral matching against reference databases. Differential metabolites were defined by fold change (FC) ≥ 2 and false discovery rate (FDR) < 0.05, with variable importance in projection (VIP) > 1 as an auxiliary criterion. Final analyses were performed using MetaboAnalyst (v5.0), applying multivariate statistics and pathway enrichment to highlight metabolic responses, particularly those associated with glycolysis and the TCA cycle.

### 2.21 Establishment of the Collagenase‐Induced ICH Model

Male C57BL/6J mice (22–25 g, obtained from Beijing Vital River Laboratory Animal Technology Co., Ltd., China) were allocated into two in vivo experimental cohorts. The standard treatment cohort comprised six groups (n = 15 per group): Sham, ICH+NS, ICH+DIDS, ICH+Mdivi‐1, ICH+Free DIDS+Mdivi‐1, and ICH+NP@D+M. The LPAR1 knockdown validation cohort comprised two groups: ICH+NP@D+M+siRNA NC and ICH+NP@D+M+siLPAR1. For the endpoint analyses, six mice per group were analyzed. ICH was induced using a stereotaxic injection of collagenase. Under isoflurane anesthesia (2%), mice were secured in a stereotaxic frame (RWD Life Science), and the scalp was shaved and sterilized. A burr hole was drilled at 0.8 mm posterior to the bregma and 2.0 mm lateral to the midline on the right hemisphere. A microsyringe was used to slowly inject 0.0375 U of collagenase VII (Sigma–Aldrich) dissolved in 0.5 µL sterile saline at a depth of 3.5 mm, at a rate of 0.1 µL/min. The needle was retained for an additional 5 min post‐injection to prevent reflux, then slowly withdrawn, and the skull and scalp were sealed.

The Sham group underwent the same surgical procedure without collagenase injection. Standard treatments were administered via tail‐vein injection at 1 and 24 h after ICH induction, including saline (ICH+NS), free‐form drugs (ICH+DIDS, ICH+Mdivi‐1, ICH+Free DIDS+Mdivi‐1), or the nanoplatform (ICH+NP@D+M), each at an equimolar dosage of 2 mg/kg for both DIDS and Mdivi‐1. The LPAR1 knockdown validation cohort followed the siRNA and NP@D+M administration schedule described below. All procedures were performed by personnel blinded to the experimental conditions. Neurological deficits and general physiological status were monitored throughout the study (Figure S3).

### 2.22 In Vivo Administration and Fluorescence Imaging of the Nanoplatform

Systemic delivery of the nanoplatform was achieved via tail vein injection. Prior to administration, nanoparticles were labeled with the near‐infrared fluorescent dye DiR (Thermo Fisher). Specifically, DiR (dissolved in ethanol at a final concentration of 5 µM) was incubated with the nanoparticle suspension in the dark for 30 min. Free dye was subsequently removed by ultracentrifugation (15 000 rpm, 30 min), and the pellet was resuspended in PBS. Labeling efficiency was assessed by fluorescence spectrophotometry to ensure adequate probe encapsulation. Nanoparticles (200 µL per dose, containing 2 mg/kg each of DIDS and Mdivi‐1) were administered at 1 and 24 h post‐model induction. Sham and vehicle (NS) groups received equal volumes of PBS. Following each injection, mice were monitored for body weight and neurological symptoms.

In vivo fluorescence imaging was performed using an IVIS Spectrum system at 2, 6, 12, and 24 h after intravenous injection. The same mice were serially imaged at each time point under isoflurane anesthesia using identical acquisition settings. Brain regions of interest were defined consistently across animals and time points and quantified using Living Image software. Immediately after the 24‐h imaging session, mice were euthanized, and the brain, liver, lungs, kidneys, and spleen were harvested for terminal ex vivo fluorescence imaging. Fluorescence signals from the excised organs were quantified using identically sized organ‐specific regions of interest. Brain tissues collected at 24 h were subsequently used for coronal‐section imaging and CD31‐based confocal analysis.

### 2.23 Quantitative Analysis of Intravascular and Extravascular Fluorescence Distribution in Cerebral Vessels

To accurately evaluate the BBB penetration efficiency of nanoparticles, frozen brain sections were subjected to three‐dimensional confocal laser scanning imaging and quantitative analysis. Briefly, DiR‐labeled nanoparticles were detected in the red channel (excitation/emission: 748/780 nm), whereas brain microvascular endothelium was labeled with a CD31 antibody and detected in the green channel (excitation/emission: 488/520 nm). Z‐stack scanning was performed with a step size of 0.5 µm and a total thickness of 10 µm. The acquired images were further analyzed using ImageJ software (v1.53, NIH).

Region definition: Based on CD31‐positive signals, the scanned area was divided into three regions: (1) intravascular region, defined as the area within the CD31‐positive signal, corresponding to the luminal side of the vessel; (2) vascular wall transition region, defined as the area 0–2 µm outside the CD31‐positive signal; and (3) extravascular parenchymal region, defined as the brain parenchymal area ≥2 µm outside the CD31‐positive signal.

Quantitative analysis: The integrated density of DiR red fluorescence was measured separately in the three regions described above. The parenchymal ratio (PR) was calculated using the following formula: PR (%) = DiR fluorescence intensity in the extravascular parenchymal region / (DiR fluorescence intensity in the intravascular region + DiR fluorescence intensity in the extravascular parenchymal region + DiR fluorescence intensity in the vascular wall region) × 100%. For each group, 15–20 intact vascular cross‐sections were randomly selected from three independent animals, with 5–6 fields analyzed per animal. Quantification was independently performed by two observers blinded to the group allocation. Data are presented as the mean ± standard deviation, and comparisons among groups were performed using one‐way analysis of variance [42].

### 2.24 Assessment of Mitochondrial Function in Brain Tissue

At 48 h after the final injection, mice were anesthetized and euthanized. The brain was rapidly harvested and rinsed in ice‐cold saline to remove surface blood. Ischemic tissue from the ipsilateral basal ganglia was weighed and immediately subjected to mitochondrial isolation using a commercial kit (Thermo Fisher), following the manufacturer's protocol. Briefly, tissue was minced and homogenized on ice in lysis buffer (30 strokes), followed by differential centrifugation (600 g for 10 min to remove nuclei and debris, then 7000 g for 10 min to collect crude mitochondria). The pellet was washed, re‐centrifuged, and resuspended in mitochondrial storage buffer. All procedures were conducted at 4°C to preserve mitochondrial integrity.

ΔΨm was assessed using the JC‐1 assay kit (Beyotime). Equal amounts of mitochondrial suspension (20 µg protein) were incubated with JC‐1 dye at 37°C for 20 min. After washing, red (aggregated JC‐1, Ex/Em = 525/590 nm) and green (monomeric JC‐1, Ex/Em = 490/530 nm) fluorescence signals were measured using a microplate reader (BioTek Synergy H1). The red/green fluorescence ratio was calculated to indicate Δψm. ATP levels were determined using a luminescent ATP detection kit (Beyotime). Briefly, 10 µL of mitochondrial solution was mixed with the luminescent substrate and incubated according to the protocol. Luminescence was recorded on the same reader, and ATP concentrations were calculated based on a standard curve and expressed as nmol/mg protein.

Mitochondrial ROS production was measured using the MitoSOX Red probe. A 10 µg aliquot of mitochondrial suspension was incubated with 5 µM MitoSOX at 37°C for 15 min in the dark. After PBS washes, fluorescence was recorded (Ex/Em = 510/580 nm).

In the mPTP opening assay, cyclosporin A (CsA; CAS: 59865‐13‐3, Cat. No.: SML1018, Sigma–Aldrich) was included as a positive control. Specifically, mice received intracerebroventricular injection of CsA (10 mg/kg) 10 min before tissue collection to evaluate the protective effect of specific mPTP inhibition on mitochondrial morphology [43]. The mPTP opening rate was evaluated using a commercial assay kit (Beyotime). Following Ca^2^^+^ stimulation, changes in absorbance at 540 nm were continuously monitored to determine the rate of pore opening. All measurements were performed in triplicate. Mitochondrial protein concentrations were quantified using the BCA assay, and results were expressed as mean ± standard deviation (SD). Statistical analyses were subsequently conducted to evaluate intergroup differences.

### 2.25 Histopathological and Immunohistochemical Analysis

Brain tissues were harvested 48 h after the final administration. Mice were euthanized under anesthesia and perfused transcardially with saline, followed by fixation in 4% paraformaldehyde for 24 h. The basal ganglia region and surrounding cortex were collected. Part of the tissue samples was used for paraffin embedding, while the remaining samples were used for other related analyses. Coronal sections (5 µm thickness) were prepared. Hematoxylin and eosin (H&E) staining was performed using standard protocols: sections were dewaxed, rehydrated, stained with hematoxylin for 5 min, rinsed, differentiated with 0.5% hydrochloric acid ethanol, blued, and counterstained with 0.5% eosin for 2 min. Slides were dehydrated, cleared, and mounted using neutral resin. Nissl staining was carried out with 0.1% toluidine blue at 60°C for 30 min, followed by differentiation with 95% ethanol, rapid dehydration, and coverslipping. Neuronal morphology, Nissl body integrity, necrotic vacuolization, and structural alterations were observed under a light microscope (Leica DM3000) at 400× magnification.

For immunohistochemistry, sections were dewaxed, rehydrated, and incubated with 3% hydrogen peroxide for 10 min at room temperature to quench endogenous peroxidase activity. Antigen retrieval was conducted in citrate buffer at 95°C for 15 min. After cooling, nonspecific binding was blocked with 5% BSA for 1 h. Sections were then incubated overnight at 4°C with primary antibodies: NeuN (1:200), GFAP (1:500), Iba1 (1:500), and Cleaved Caspase‐3 (1:300; Cell Signaling Technology). The following day, after three PBS washes, sections were incubated with HRP‐conjugated secondary antibodies (1:500; Cell Signaling) for 1 h at room temperature. Visualization was performed using diaminobenzidine (DAB) substrate, and nuclei were counterstained with hematoxylin. Negative controls were included by omitting the primary antibodies. The positive staining area and integrated optical density (IOD) of each marker were quantified in five randomly selected high‐power fields (400×) using ImageJ software (NIH). Expression levels were expressed as the ratio of IOD to area. Results were presented as mean ± SD, and group comparisons were used to assess differences in neuronal survival, gliosis, and apoptosis.

### 2.26 Behavioral and Cognitive Function Assessment

Neurological function was evaluated on days 1, 3, and 7 post‐surgery using the Garcia scoring system, independently assessed by two blinded investigators. The scoring scale comprises six subtests: spontaneous activity, symmetry of limb movement, forelimb extension, climbing ability, response to touch, and response to vibrissae stimulation. Each subtest was scored from 0 to 3, with a maximum total score of 18. The average score from the two raters was recorded as the final score for each mouse.

Cognitive performance was assessed using the Morris water maze on postoperative days 14 to 18. The circular pool (120 cm in diameter) was maintained at 25°C ± 1°C and divided into four quadrants. A hidden platform was placed 2 cm below the water surface in a fixed quadrant. During acquisition training, mice underwent daily training trials (maximum duration: 60 s), and escape latency was recorded. If a mouse failed to locate the platform within 60 s, it was guided to the platform and allowed to remain there for 60 s. Endpoint escape latency was summarized from the terminal acquisition assessment. A subsequent probe trial was conducted by removing the platform and allowing the mouse to swim freely for 60 s; the time spent in the target quadrant and the number of crossings over the previous platform location were recorded. Data were analyzed using an automated video tracking system (SMART 3.0, Panlab).

The Y‐maze test was conducted on postoperative day 20. Each arm of the maze measured 30 cm in length, 6 cm in width, and 15 cm in height, forming a 120° angle between arms. Mice were individually placed in the central zone and allowed to explore freely for 8 min. Spontaneous alternation behavior, an indicator of working memory, was quantified as the ratio of consecutive entries into three different arms to the total number of arm entries minus two. The novel object recognition (NOR) test was performed on postoperative day 22. During the habituation phase, mice were allowed to explore an empty arena for 10 min. In the training phase, two identical objects were introduced, and the mice explored for another 10 min. In the test phase, one familiar object was replaced with a novel one, and mice were observed for 5 min. The discrimination index, representing recognition memory, was calculated as the percentage of time spent exploring the novel object relative to the total exploration time: Recognition Index = (Time with Novel Object / Total Exploration Time) × 100%.

### 2.27 LPAR1 Knockdown Validation and Safety Evaluation

For the LPAR1 knockdown validation experiment, mice received a tail‐vein injection of siLPAR1 or non‐targeting siRNA (siRNA NC) at 50 µg/kg 12 h before ICH induction. At T = 0, the collagenase‐induced ICH model was established. Both groups subsequently received NP@D+M by tail‐vein injection at 1 and 24 h after ICH induction. LPAR1 knockdown was confirmed by qRT‐PCR and Western blotting. Neurological function, ATP content, mitochondrial membrane potential, and mitochondrial ROS were subsequently evaluated. Safety assessments were conducted seven days after the final administration. Mice were euthanized under anesthesia, and blood samples were collected via cardiac puncture. Serum was separated (4°C, 3000 rpm, 15 min) and analyzed using a fully automated biochemical analyzer (Roche Cobas c702) to determine liver and kidney function markers, including alanine aminotransferase (ALT), aspartate aminotransferase (AST), blood urea nitrogen (BUN), and creatinine (Cr). Major organs (heart, liver, spleen, lungs, and kidneys) were fixed in 4% paraformaldehyde for 24 h, embedded in paraffin, sectioned, and stained with hematoxylin and eosin (H&E) to evaluate systemic toxicity. Inflammation was graded using a 0–4 semiquantitative severity scale (0, absent; 1, minimal; 2, mild; 3, moderate; and 4, severe). Each mouse was treated as the experimental unit, and the recorded final score for each organ was used as one animal‐level observation. Scores are presented as median (interquartile range). Control and NP@D+M groups (n = 6 per group) were compared separately for each organ using two‐sided Mann–Whitney U tests, with Holm adjustment across the five organ‐specific comparisons.

All evaluations were conducted by investigators blinded to group allocation. Serum biochemistry data are presented as mean ± SD. Blood and histological indicators were compared between the Control and NP@D+M groups to evaluate the systemic toxicity and biocompatibility of the nanoplatform following repeated dosing.

### 2.28 Statistical Analysis

All statistical analyses were performed using SPSS 26.0 (IBM), GraphPad Prism 9.0 (GraphPad Software), and R version 4.1.2 (R Foundation). Continuous variables were tested for normality using the Shapiro–Wilk test. For data satisfying normal distribution and homogeneity of variance, results were expressed as mean ± SD. One‐way analysis of variance (ANOVA) was applied for multiple‐group comparisons, followed by Tukey's HSD or Bonferroni post hoc tests based on distribution characteristics. For non‐normally distributed or heteroscedastic data, the Kruskal–Wallis test was used with Dunn's test for pairwise comparisons.

For time‐series data (e.g., behavioral scores, body weight), repeated‐measures ANOVA was employed to evaluate both time and group effects. For scRNA‐seq data, differential gene analysis was conducted using either the Wilcoxon rank‐sum test or logistic regression, with multiple testing correction via the Benjamini‐Hochberg method. An FDR of <0.05 was considered statistically significant. Proteomic data were analyzed using the DEP package in R, with differential proteins defined by an FC >1.5 and *p* < 0.05.

All *p*‐values were two‐tailed, and statistical significance was defined as *p* < 0.05. Significant results are indicated in figures and legends as ^*^ *p* < 0.05, ^**^ *p* < 0.01, and ^***^ *p* < 0.001. The full statistical workflow and R scripts have been archived and are available upon request to ensure reproducibility.

### 2.29 Figure Preparation Statement

AI‐assisted tools were used only to support the preparation of explanatory schematic elements in the schematic illustrations and Graphical Abstract. All final illustrations, labels, and scientific content were reviewed and edited by the authors to ensure scientific accuracy, consistency with the manuscript text, and compliance with journal policies. No AI tools were used to generate or modify primary experimental images or quantitative data.

## 3 Results

### 3.1 Physicochemical Characterization of the Nanoparticles

DLS analysis demonstrated that the biomimetic membrane‐coated, PLGA‐PEG nanoparticles with H_2_O_2_‐dependent drug‐release behavior co‐loaded with DIDS and Mdivi‐1 (NP@D+M) exhibited a uniform size distribution, with an average hydrodynamic diameter of 162.3 ± 4.7 nm. This was significantly larger than that of uncoated nanoparticles (134.5 ± 3.2 nm, *p* < 0.001), suggesting successful membrane coating. The PDI remained below 0.15, indicating good colloidal stability (Figure 1A). Zeta potential analysis further showed that the surface charge of uncoated nanoparticles (Bare NP) was −19.6 ± 1.3 mV. The isolated endothelial cell membrane vesicles (Membrane) exhibited a typical highly negative surface charge of −32.5 ± 1.8 mV. After biomimetic membrane coating, the surface charge of NP@D+M nanoparticles significantly decreased to −31.2 ± 2.0 mV (*p* < 0.001, Figure 1B). The pronounced negative shift in Zeta potential after coating, together with its close similarity to that of the purified cell membrane, supports the successful wrapping of endothelial cell membranes on the nanoparticle surface. TEM visualized a well‐defined, dense peripheral membrane layer with a thickness of approximately 8–12 nm, consistent with a uniform biomimetic coating (Figure 1C).

**FIGURE 1:**
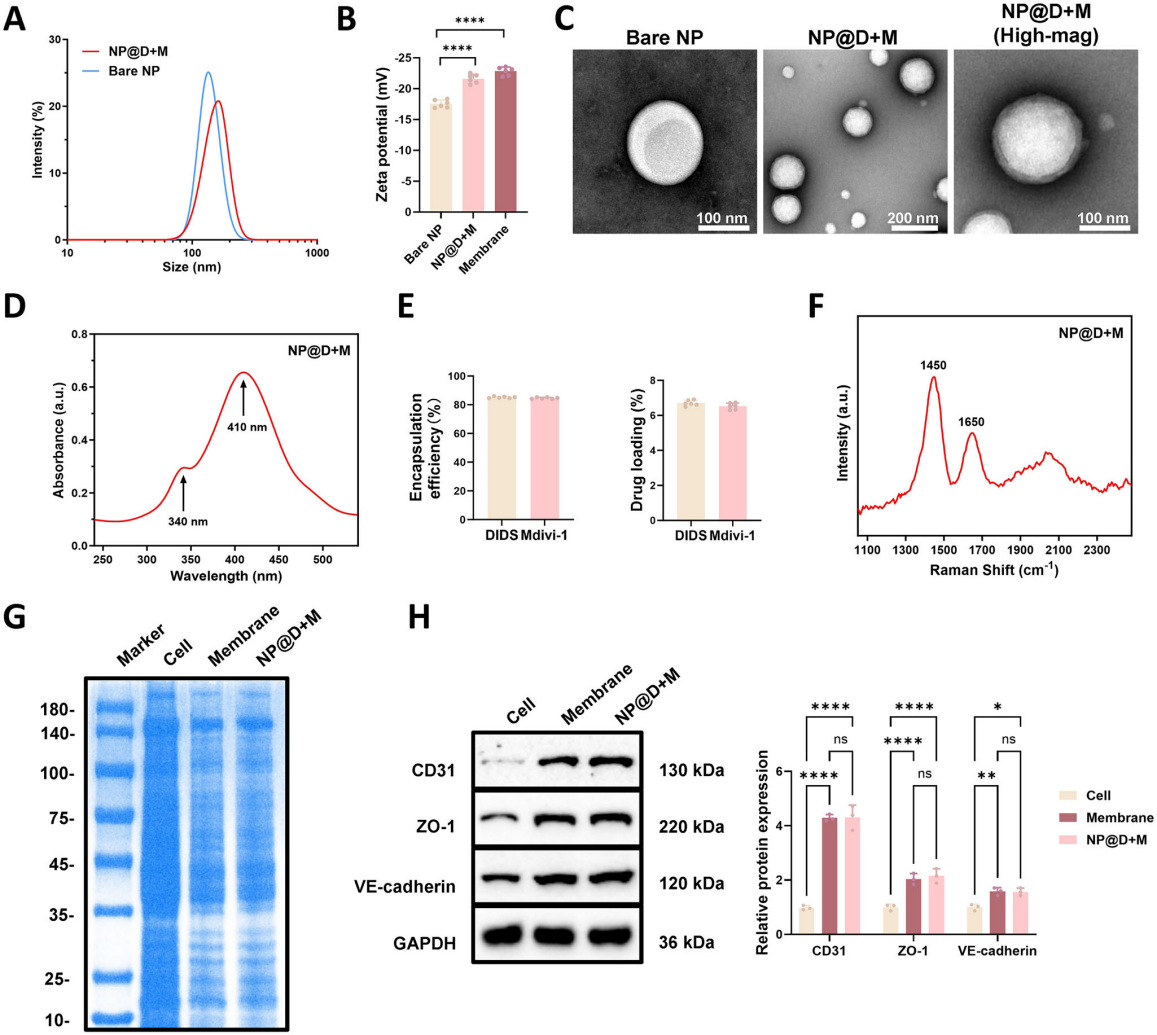
Physicochemical characterization of ROS‐responsive biomimetic nanoparticles. Note: (A) DLS analysis showing increased particle size in the NP@D+M group compared to the uncoated group. (B) Zeta potential measurements indicate a significant decrease in surface charge following biomimetic membrane coating, with values approaching those of the isolated purified endothelial membrane. (C) TEM images reveal the nanoparticle morphology and uniform membrane coating (red arrows). Scale bar: 100 nm. (D) UV–vis spectroscopy confirming the dual absorption peaks characteristic of DIDS and Mdivi‐1. (E) LC–MS/MS‐based batch mass‐balance quantification of the encapsulation efficiency and drug loading of DIDS and Mdivi‐1 in the final purified NP@D+M formulation. Data are presented as mean ± SD from six independently prepared formulation batches (n = 6). No statistical comparison was performed between DIDS and Mdivi‐1. (F) Raman spectroscopy identifies characteristic protein and lipid peaks on the NP@D+M surface (indicated in blue). (G) SDS‐PAGE with Coomassie Brilliant Blue staining was used to analyze total protein profiles of source endothelial cell lysates (Cell), extracted cell membranes (Membrane), and membrane‐coated nanoparticles (NP@D+M). (H) Western blot analysis and quantification of key endothelial marker proteins, including CD31, ZO‐1, and VE‐cadherin. Data are presented as mean ± SD, n = 3; ^*^ *p* < 0.05, ^**^ *p* < 0.01, ^****^ *p* < 0.0001. In this figure, “Bare NP” refers to drug‐loaded nanoparticles without cell membrane coating (PLGA‐PEG@D+M). Both Bare NP and NP@D+M were loaded with DIDS and Mdivi‐1, with the key difference being that NP@D+M was further coated with brain microvascular endothelial cell membranes.

Ultraviolet–vis spectroscopy showed distinct absorption peaks for DIDS and Mdivi‐1 at 340 and 410 nm, respectively, and the NP@D+M suspension displayed a dual‐peak profile, verifying successful drug encapsulation (Figure 1D). LC–MS/MS analysis showed that the encapsulation efficiencies of DIDS and Mdivi‐1 in the final purified NP@D+M formulation were 85.2% and 84.6%, respectively, whereas the corresponding drug‐loading values were 6.7% and 6.5% (Figure 1E). These values represent formulation‐specific characteristics of the final NP@D+M preparation and were not interpreted as evidence that membrane coating itself increased EE or DL. Raman spectroscopy indicated the presence of characteristic protein and lipid peaks at 1450 and 1650 cm^−^^1^, respectively, on the NP@D+M surface—features absent in uncoated nanoparticles—demonstrating the preservation of membrane components post‐coating (Figure 1F).

Considering that the key function of targeted delivery depends on the presence of specific receptors on the cell membrane surface, SDS‐PAGE and Western blot analyses were performed to verify the retention of key membrane proteins in each component. Coomassie Brilliant Blue staining showed that the total protein band pattern of the NP@D+M group was highly consistent with that of the purified cell membrane (Membrane) group, but clearly different from that of the source whole‐cell lysate (Cell) group. This result confirmed that the complex membrane protein network was largely preserved during the nano‐coextrusion process (Figure 1G). Further Western blot analysis showed strong specific expression of core marker proteins involved in blood–brain barrier translocation and homologous targeting, including CD31, ZO‐1, and VE‐cadherin, in both the Membrane and NP@D+M groups (Figure 1H). These findings demonstrate that the biomimetic nanoplatform successfully retained key targeting molecules from the source cells, providing a solid basis for efficient targeted delivery in vivo.

Altogether, these findings validate the structural uniformity, physicochemical stability, and measured drug encapsulation performance of the engineered NP@D+M nanoplatform without attributing the DL/EE values to membrane coating alone.

### 3.2 ROS‐Responsive Drug Release Behavior

To evaluate the platform's responsiveness to oxidative stress, cumulative drug release profiles of NP@D+M were assessed under physiological (0 µM H_2_O_2_) and pathological ROS‐mimicking (500 µM H_2_O_2_) conditions. Under elevated H_2_O_2_ conditions, NP@D+M demonstrated markedly accelerated drug release. Specifically, under 100 µM H_2_O_2_ stimulation for 48 h, the cumulative release rates of DIDS and Mdivi‐1 reached 45.3% ± 3.8% and 42.6% ±3.5%, respectively. These release levels were significantly higher than those observed under simulated physiological conditions without H_2_O_2_ stimulation (0 µM H_2_O_2_; DIDS: 18.4% ± 2.1%; Mdivi‐1: 16.9% ± 1.8%; *p* < 0.01), but remained significantly lower than the peak release levels induced by high H_2_O_2_ stimulation with 500 µM H_2_O_2_ (DIDS: 72.8% ± 4.6%; Mdivi‐1: 69.5% ± 5.3%; *p* < 0.001) (Figure 2A). Kinetic modeling indicated that release under elevated H_2_O_2_ conditions better fit the Higuchi diffusion model (R^2^ = 0.967), consistent with accelerated drug release under elevated H_2_O_2_ conditions (Figure 2B). Temporal analysis revealed the most rapid drug release within the first 12 h, followed by a plateau phase (Figure 2C).

**FIGURE 2:**
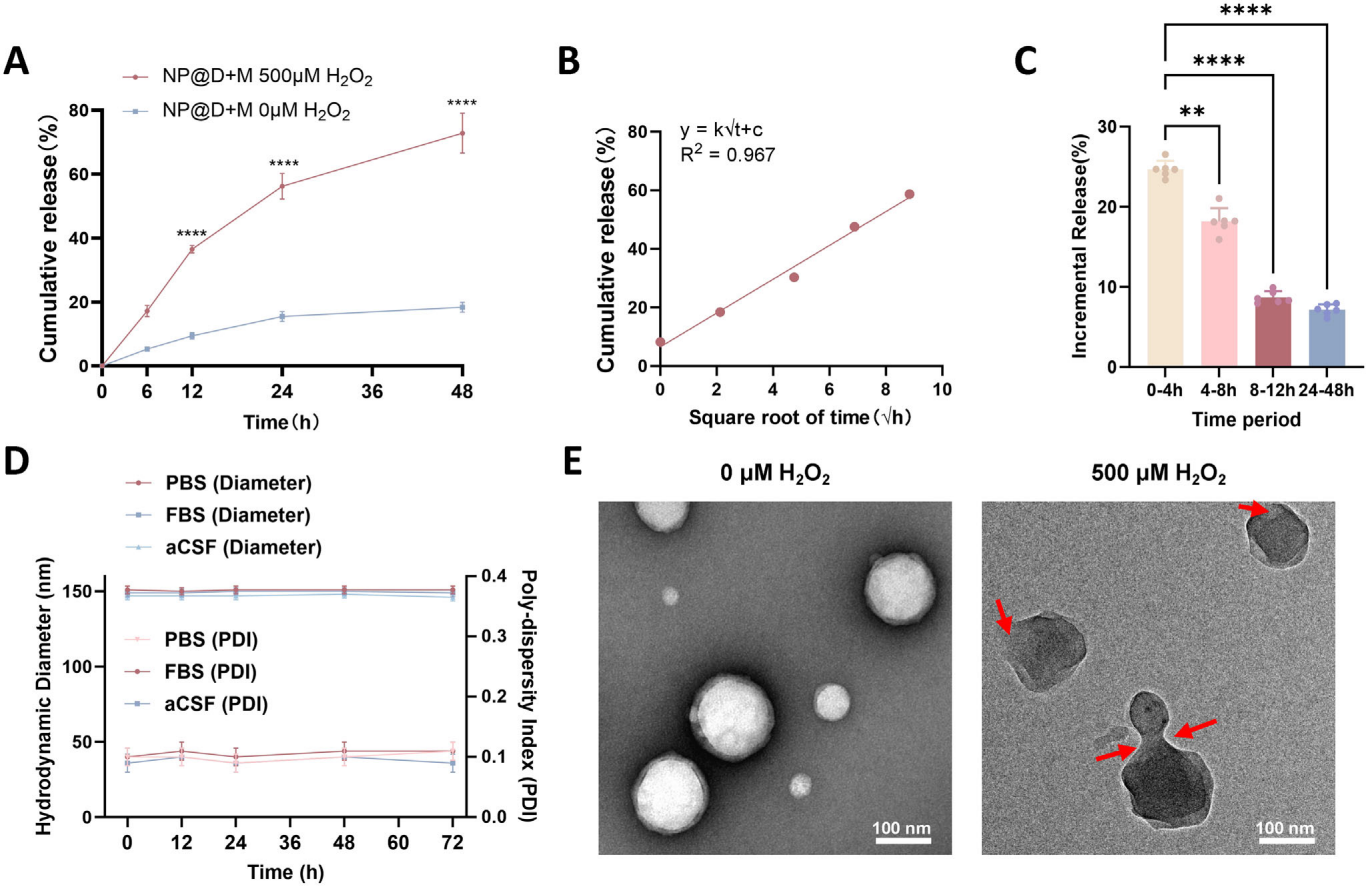
ROS‐responsive controlled release behavior of the nanoplatform under oxidative stress conditions. Note: (A) Cumulative release profiles of DIDS and Mdivi‐1 over 48 h at varying H_2_O_2_ concentrations, demonstrating significantly enhanced drug release under high ROS conditions. (B) Higuchi kinetic fitting curves, showing that NP@D+M exhibits the highest correlation with the model (R^2^ = 0.967) under 500 µM H_2_O_2_. (C) Dynamic cumulative release over 0–48 h, with a markedly accelerated release rate observed during the first 12 h. (D) Dynamic DLS monitoring of the hydrodynamic diameter and polydispersity index (PDI) of NP@D+M after incubation in PBS containing 10% FBS or artificial cerebrospinal fluid (aCSF) for 72 h, showing good colloidal stability. (E) TEM images of nanoparticles exposed to 0 µM or 500 µM H_2_O_2_. Representative TEM morphology before and after treatment is shown, with arrows indicating ROS‐induced membrane rupture and structural collapse. Scale bar: 100 nm. ^**^ *p* < 0.01, ^****^ *p* < 0.0001.

NP@D+M maintained good colloidal stability in the tested biological media over the observation period (Figure 2D). Regarding ROS responsiveness, TEM images directly revealed the structural changes before and after stimulation. Untreated NP@D+M displayed a regular spherical morphology with a clearly visible membrane coating. In contrast, after exposure to 500 µM H_2_O_2_ for 4 h, the originally intact nanostructure was disrupted, accompanied by extensive damage to the biomimetic membrane layer and collapse of the nanoparticle framework (Figure 2E). This morphological evidence was highly consistent with the accelerated drug‐release kinetics described above, further confirming the on‐demand drug‐release capability of this nanoplatform.

These findings demonstrate H_2_O_2_‐dependent drug‐release behavior, with elevated H_2_O_2_ conditions accelerating DIDS and Mdivi‐1 release from the nanoparticle formulation.

### 3.3 Enhancement of Cell Viability in the ICH In Vitro Model by NP@D+M

To ensure the reliability of subsequent experiments, the purity of primary cells cultured in vitro for 7 days was verified in this study (Figure S4). Immunofluorescence staining showed that, under the Neurobasal+B27 serum‐free culture system, most adherent cells highly expressed the neuron‐specific nuclear marker NeuN (green fluorescence), whereas only a few cells expressed the astrocyte marker GFAP (red fluorescence) (Figure S4A). Quantitative analysis further showed that NeuN‐positive neurons accounted for 94.2% ± 1.5% of total DAPI‐positive cells, while GFAP‐positive glial cells accounted for only 3.5% ± 0.8% (Figure S4B). These results confirmed that a high‐purity primary cortical neuron culture system was successfully established, minimizing potential interference from glial cells in the subsequent evaluation of NP@D+M intervention.

To systematically evaluate the neuroprotective efficacy of NP@D+M under oxidative stress conditions, an in vitro ICH model was established using combined LPS and Hb stimulation. Cell viability assessed by the CCK‐8 assay revealed a significant reduction in neuronal viability to 69.3% ± 4.8% following 24 h of LPS/Hb exposure (vs. control, *p* < 0.001), indicating successful induction of cytotoxic injury (Figure 3A). Subsequent treatment with either free drugs (DIDS + Mdivi‐1) or NP@D+M significantly improved neuronal survival. Notably, NP@D+M restored viability to 91.4% ± 3.7%, substantially outperforming the free drug group (80.2% ± 4.5%, *p* < 0.01) and closely approaching control levels (*p* > 0.05). These findings were corroborated by live/dead fluorescence staining, which showed a marked increase in green fluorescence (viable cells) and a reduction in red fluorescence (non‐viable cells) in the NP@D+M group (Figure 3B). Quantitative analysis confirmed a significantly higher proportion of live cells in the NP@D+M group (90.2% ± 3.9%) compared to free drugs (*p* < 0.01, Figure 3C). After confirming the protective effect of NP@D+M on neuronal viability, we further assessed its ability to restore LPS/Hb‐induced neurite structural injury using MAP2 immunofluorescence staining combined with confocal imaging (Figure 3D,E). Neurons in the normal control group displayed a slender, intact, and highly branched MAP2‐positive dendritic network. In contrast, neurons in the LPS/Hb‐treated ICH+NS group exhibited severe dendritic fragmentation, shrinkage, and degeneration, with the average neurite length significantly reduced to 35.2% ± 5.1% of that in the control group (*p* <0.001), confirming marked structural damage induced by iron accumulation and ROS. Intervention with free drugs (DIDS+Mdivi‐1) only partially alleviated dendritic retraction, restoring the average neurite length to 58.4% ± 6.3% of the control level. Notably, neurons in the NP@D+M‐treated group showed substantial preservation of the dendritic network, with the average neurite length significantly increased to 86.7% ± 4.8% of the control level, which was significantly higher than that in the free drug group (*p* <0.01). These morphological findings further demonstrate that NP@D+M effectively counteracts oxidative stress and maintains neuronal structural integrity.

**FIGURE 3:**
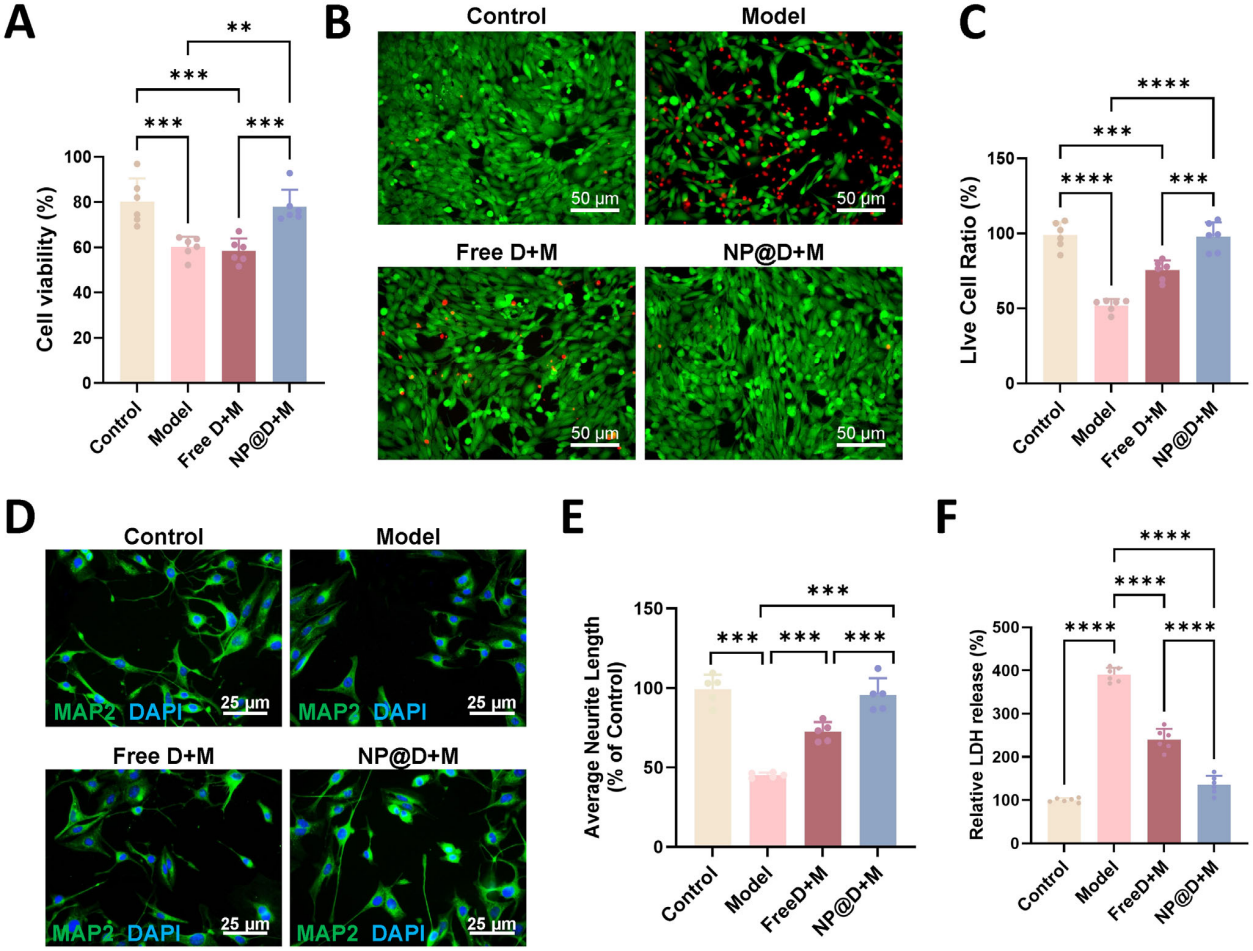
NP@D+M significantly enhances neuronal viability in an LPS/Hb‐induced in vitro ICH model. Note: (A) CCK‐8 assay results show a significant reduction in neuronal viability in the ICH+NS group, while treatment with NP@D+M markedly restored viability to near‐control levels (n = 6 per group). (B) Fluorescent images of primary neurons stained for live/dead assessment. Live cells are labeled with Calcein‐AM (green), and dead cells with PI (red); the NP@D+M group exhibited a notably reduced cell death rate (scale bar: 50 µm). (C) Quantitative analysis of live/dead cell ratios revealed a significantly higher proportion of live cells in the NP@D+M group compared to the free drug group. (D) Representative confocal immunofluorescence images of MAP2 staining in primary cortical neurons. Green: MAP2‐labeled neuronal dendrites; blue: DAPI‐labeled nuclei. Scale bar: 50 µm. (E) Quantitative analysis of the average neurite length of MAP2‐positive neurons based on representative confocal immunofluorescence images. n = 5 independent fields. (F) Quantitative analysis of LDH release in the culture medium of primary neurons after different treatments, indicating the degree of cell membrane rupture and necrosis. Data are presented as mean ± SD; ^**^ *p* < 0.01, ^***^ *p* < 0.001, ^****^ *p* < 0.0001.

To further biochemically confirm the protective effect of NP@D+M on neuronal membrane integrity, LDH release in the cell culture supernatant was quantitatively measured in each group (Figure 3F). The results showed that LPS/Hb stimulation caused a sharp increase in LDH release in the ICH+NS group, reaching 3.8‐fold that of the control group (*p* <0.001), indicating severe disruption of the cell membrane structure. Although free drug treatment (DIDS+Mdivi‐1) partially reduced LDH leakage, NP@D+M exerted a more pronounced membrane‐protective effect, as evidenced by a marked decrease in LDH release, with a statistically significant difference compared with the free drug group.

Collectively, these results demonstrate that the biomimetic nanoplatform confers robust protection of neuronal viability under ICH‐related oxidative stress, surpassing the efficacy of unencapsulated agents.

### 3.4 Targeted Uptake Efficiency of NP@D+M

To assess the targeted delivery and intracellular uptake of NP@D+M by primary cortical neurons, the nanoparticles were labeled with DiI fluorescent dye and analyzed using confocal laser scanning microscopy (Figure 4). In the control group, only minimal background red fluorescence was observed in the cytoplasm. In contrast, bare (non‐coated) nanoparticles exhibited moderate red fluorescence, suggesting non‐specific endocytosis. Strikingly, neurons treated with NP@D+M displayed intense, evenly distributed red fluorescence throughout the cytoplasm and perinuclear region, indicating enhanced cellular uptake facilitated by the biomimetic membrane. Quantitative analysis showed that the mean fluorescence intensity in the NP@D+M group reached 178 ± 12, an ∼85% increase over the bare NP group (96 ± 10, *p* < 0.001) and far exceeding the free DiI control (28 ± 6, *p* < 0.001). Furthermore, 3D reconstruction confirmed widespread intracellular distribution of the nanoplatform. These findings highlight the superior targeting capability of NP@D+M and its potential for precise neuronal drug delivery under pathological conditions (Figure 4A–C).

**FIGURE 4:**
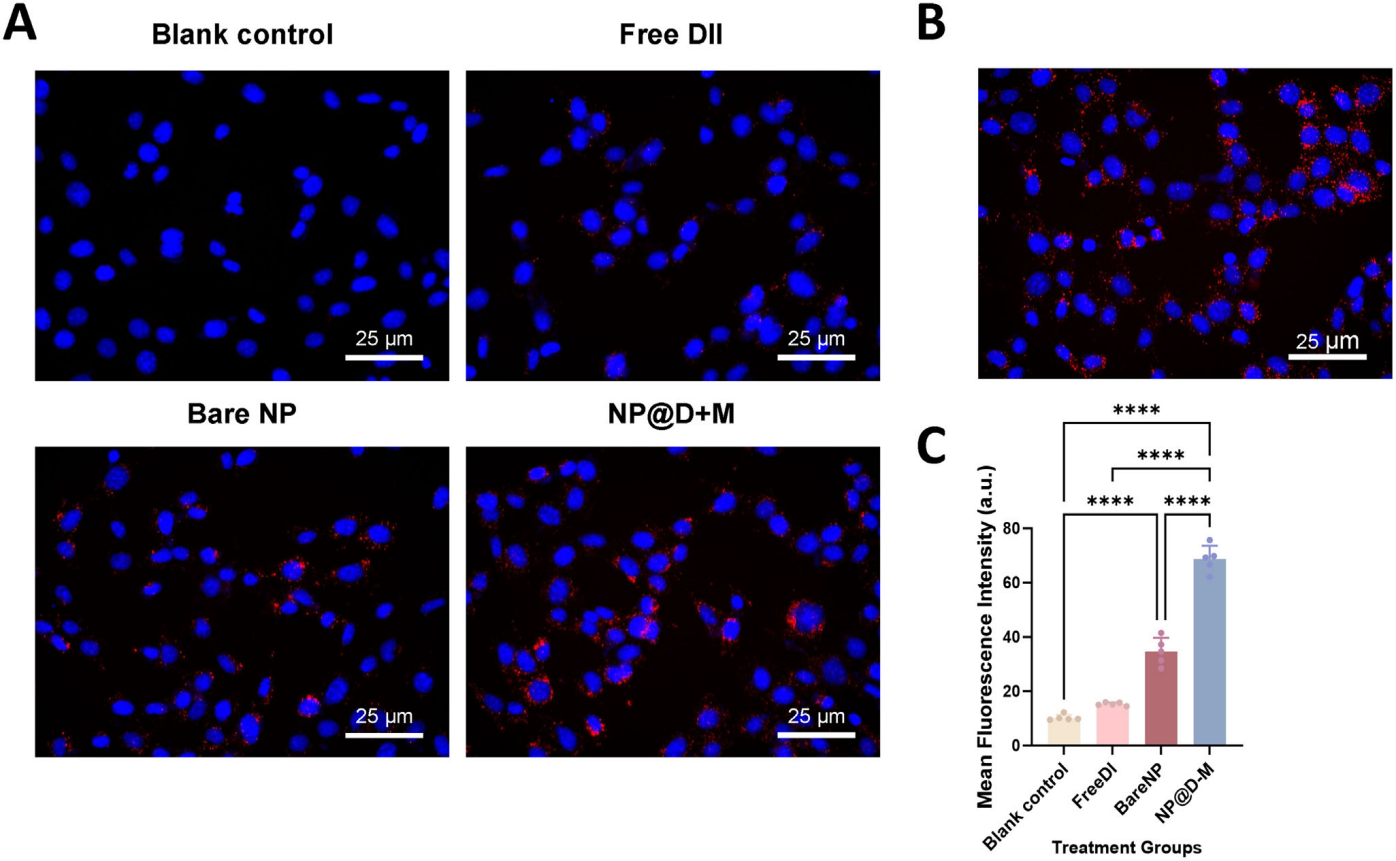
Targeted uptake and intracellular fluorescence distribution of NP@D+M in primary neurons. Note: (A) Confocal fluorescence microscopy images showing intracellular DiI distribution in neurons across different treatment groups. Red: DiI fluorescence; blue: nuclei stained with Hoechst. The NP@D+M group demonstrated markedly enhanced cytoplasmic fluorescence, indicating superior cellular uptake. (B) Three‐dimensional Z‐stack reconstruction images display red fluorescence localized in the cytoplasm and around the nucleus, suggesting efficient nanoparticle internalization. (C) Quantitative analysis of mean fluorescence intensity shows significantly higher signal in the NP@D+M group compared to the bare NP and free probe groups (n = 5, ^****^ *p* < 0.0001).

### 3.5 Restoration of ΔΨm

To evaluate the effect of NP@D+M on mitochondrial function, JC‐1 staining was used to monitor changes in ΔΨm across different treatment groups (Figure 5). In control neurons, JC‐1 fluorescence was predominantly red (aggregated form), yielding a stable red/green ratio of 1.00 ± 0.06, indicative of intact membrane potential. In contrast, neurons in the Model group showed a sharp decline in red fluorescence and a concomitant increase in green fluorescence, with the red/green ratio dropping to 0.42 ± 0.05 (*p* < 0.001), reflecting severe mitochondrial depolarization. Treatment with free DIDS and Mdivi‐1 partially restored membrane potential (ratio: 0.66 ± 0.05, *p* < 0.01 vs. ICH+NS). Notably, the NP@D+M group achieved a more pronounced recovery, with a red/green ratio of 0.83 ± 0.04 (*p* < 0.01 vs. free drug), approaching control values (*p* > 0.05). These results underscore the capacity of NP@D+M to effectively restore ΔΨm and mitigate mitochondrial dysfunction in an ICH‐mimicking environment.

**FIGURE 5:**
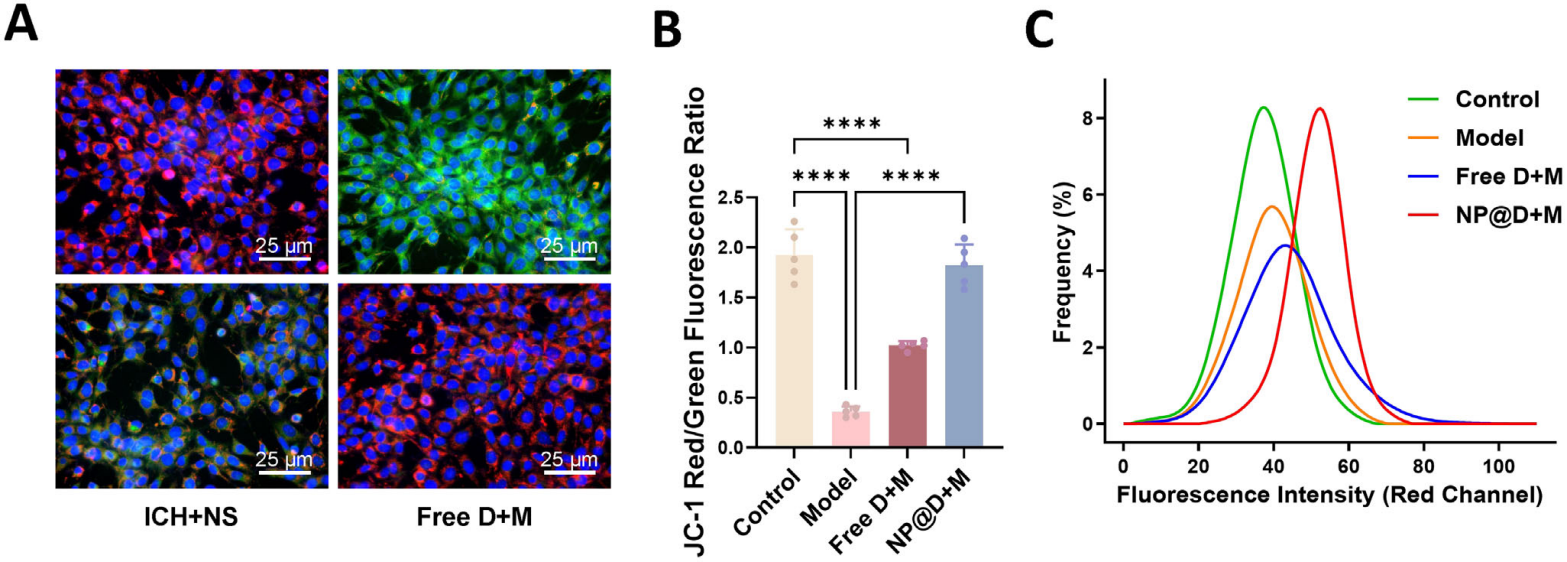
NP@D+M significantly restores ΔΨm in neurons of the ICH model. Note: (A) JC‐1 fluorescence microscopy images showing ΔΨm changes in neurons from different groups. Red fluorescence indicates JC‐1 aggregates (high ΔΨm), while green fluorescence represents JC‐1 monomers (low ΔΨm). Scale bar: 25 µm. (B) Quantification of the red/green fluorescence ratio across four groups. The Model group showed a significant decrease in ΔΨm, while NP@D+M treatment significantly restored ΔΨm to near‐control levels (n=5, ^****^ *p* < 0.0001). (C) Representative fluorescence intensity histograms demonstrate a markedly higher red fluorescence peak in the NP@D+M group compared to the free drug and Model groups.

### 3.6 Suppression of Mitochondrial ROS Generation

To further assess the ability of the nanoparticle platform to mitigate mitochondrial oxidative stress, MitoSOX Red staining was employed to quantify superoxide (O_2_•–) levels across experimental groups (Figure 6). Confocal microscopy revealed only faint red fluorescence in neurons from the Control group, indicating minimal basal ROS levels. In contrast, the Model group exhibited intense red fluorescence diffusely localized throughout the cytoplasm and neurites, consistent with excessive mitochondrial ROS accumulation (Figure 6A). Treatment with free DIDS and Mdivi‐1 reduced fluorescence moderately, whereas NP@D+M treatment led to a more pronounced attenuation, nearing baseline levels observed in the Sham group.

**FIGURE 6:**
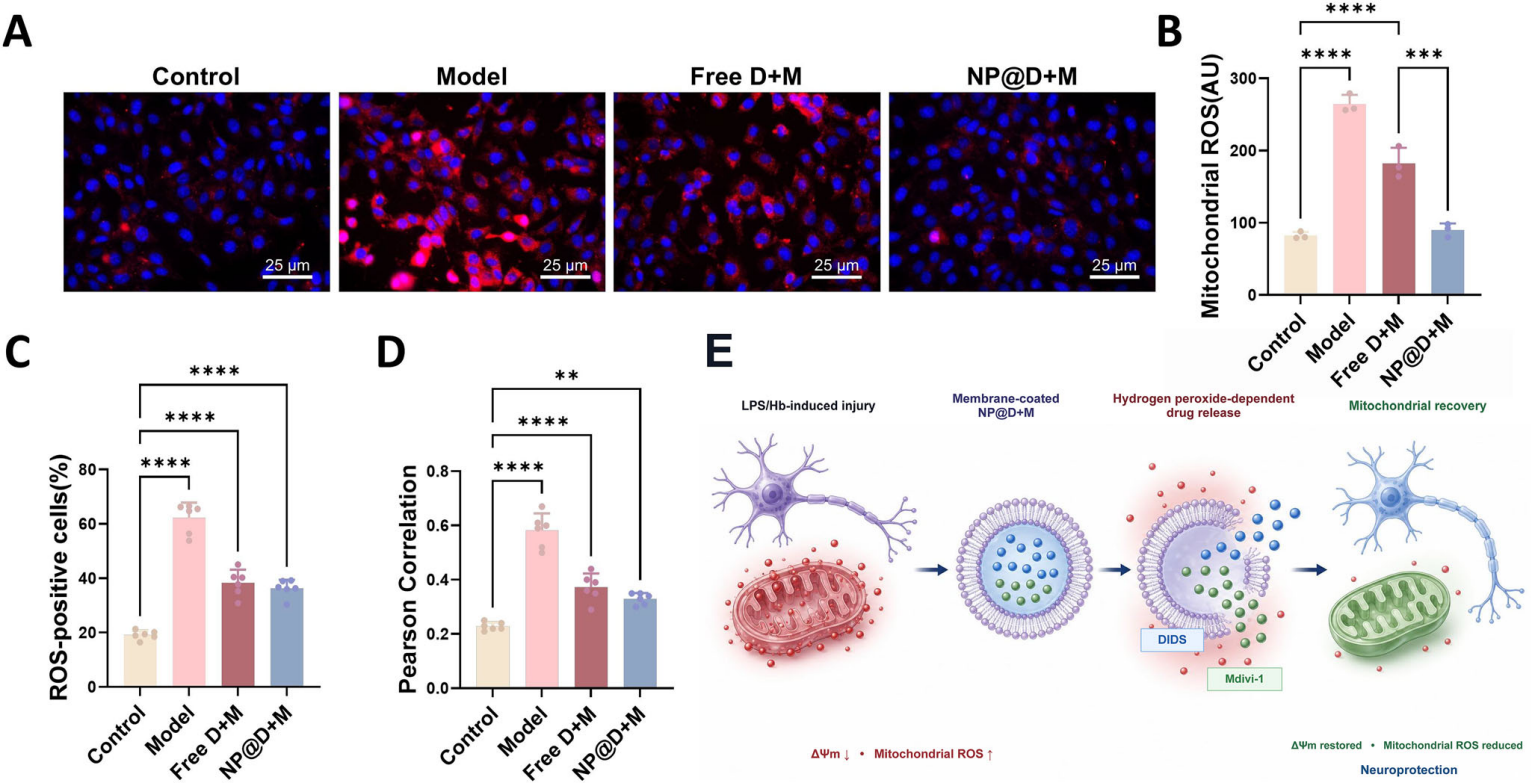
NP@D+M significantly suppresses mitochondrial ROS production in neurons under ICH conditions. Note: (A) Confocal fluorescence images of MitoSOX staining reveal mitochondrial ROS distribution in neurons from each group. Red fluorescence was weak in the Control group, markedly increased in the Model group, and substantially reduced in the NP@D+M group, approaching control levels. Scale bar: 25 µm. (B) Quantitative analysis of mitochondrial ROS fluorescence intensity shows a significant reduction in the NP@D+M group compared to the Model and free drug groups. (C) Flow cytometric analysis of the percentage of ROS‐positive neurons indicates a significant decrease in the NP@D+M group. (D) Pearson correlation coefficient analysis of JC‐1/MitoSOX co‐staining demonstrates a marked reduction in the co‐localization of ROS accumulation with ΔΨm loss in the NP@D+M group. (E) Schematic illustration of the NP@D+M nanoplatform mechanism, showing H_2_O_2_‐dependent release‐mediated co‐delivery of DIDS and Mdivi‐1, leading to restoration of ΔΨm and suppression of ROS generation, thereby exerting neuroprotective effects. ^**^ *p* < 0.01, ^***^ *p* < 0.001, ^****^ *p* < 0.0001.

Quantitative fluorescence analysis confirmed these observations: mitochondrial ROS levels in the Model group rose to 230 ± 18 AU (vs. Control: 100 ± 11, *p* < 0.001), were partially reduced by free drugs to 165 ± 14 AU (*p* < 0.01), and further suppressed to 125 ± 12 AU following NP@D+M intervention (*p* < 0.001) (Figure 6B). Flow cytometry corroborated these findings, showing a significantly reduced proportion of ROS‐positive cells in the NP@D+M group (Figure 6C).

Dual‐labeling analysis using JC‐1 and MitoSOX revealed a high degree of co‐localization between ROS accumulation and depolarized mitochondrial regions in the Model group (Pearson's coefficient: 0.78 ± 0.05). This overlap was markedly diminished following NP@D+M treatment (0.42 ± 0.04, *p* < 0.001), suggesting that the nanoparticle platform not only reduces ROS production but also contributes to mitochondrial homeostasis (Figure 6D).

Collectively, these results demonstrate that the biomimetic NP@D+M nanoparticle system effectively alleviates mitochondrial oxidative stress in the context of ICH, outperforming free drug administration (Figure 6E).

### 3.7 Enhancement of ATP Production

To evaluate the effect of NP@D+M on mitochondrial bioenergetics, intracellular ATP levels were quantified using a luminescence‐based ATP assay (Figure 7). Neurons in the Control group maintained ATP concentrations at 1.20 ± 0.08 nmol/mg protein, indicative of normal energy metabolism. Following LPS/Hb exposure, ATP levels in the Model group declined sharply to 0.48 ± 0.06 nmol/mg (*p* < 0.001), consistent with mitochondrial dysfunction and energy depletion.

**FIGURE 7:**
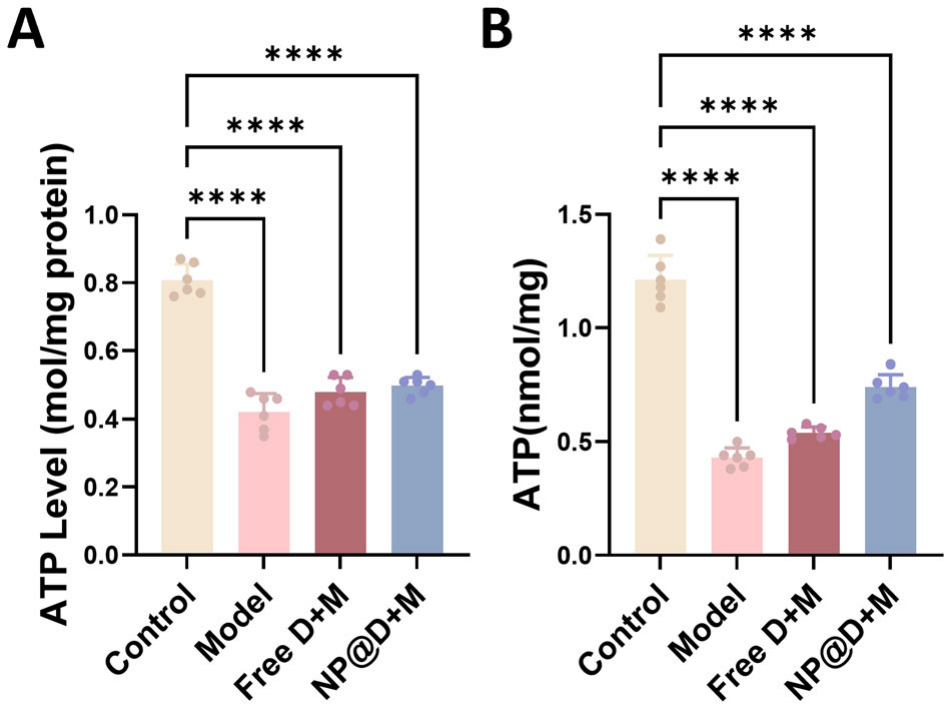
NP@D+M markedly restores intracellular ATP levels in neurons under ICH conditions. Note: (A) Bar graph showing ATP levels: the Control group maintained high ATP levels, while the Model group exhibited a significant decrease. Partial recovery was observed in the free drug group, whereas the NP@D+M group nearly restored ATP levels to baseline. (B) Representative histogram of fluorescence intensity distribution: the NP@D+M group displayed a significantly higher peak than both the Model and free drug groups. n = 6, ^****^ *p* < 0.0001.

Administration of free DIDS and Mdivi‐1 led to a partial recovery of ATP to 0.72 ± 0.05 nmol/mg (*p* < 0.01 vs. Model). Notably, NP@D+M treatment restored ATP levels more robustly, reaching 0.96 ± 0.07 nmol/mg (*p* < 0.001 vs. ICH+NS; *p* < 0.01 vs. free drugs), approximating the values observed in the Control group (*p* > 0.05). When integrated with the JC‐1 and MitoSOX co‐localization data (Figure 7), the restoration of ATP closely paralleled improvements in ΔΨm and reductions in ROS production, suggesting a synergistic effect of NP@D+M on mitochondrial energy maintenance. In summary, the biomimetic NP@D+M nanoparticle platform markedly mitigates mitochondrial dysfunction and restores ATP synthesis, thereby improving cellular energy balance in the ICH model.

### 3.8 Reduction of Neuronal Apoptosis

To comprehensively assess the neuroprotective effect of NP@D+M against apoptosis under ICH conditions, Annexin V‐FITC/PI dual staining combined with flow cytometry was employed to quantify neuronal apoptosis (Figure 8). Compared to the control group, the Model group exhibited a marked increase in apoptotic cells, with total early and late apoptosis reaching 44.3% ± 3.5%, indicating substantial apoptosis induced by the ICH microenvironment. Treatment with free DIDS and Mdivi‐1 moderately reduced the apoptotic rate to 27.2% ± 3.0% (vs. Model, *p* < 0.01), suggesting partial anti‐apoptotic efficacy. Notably, NP@D+M treatment further decreased the apoptosis rate to 18.5% ± 2.8% (*p* < 0.001), which was statistically indistinguishable from the baseline level of the control group (*p* > 0.05), highlighting the superior protective effect of the nanoplatform through targeted delivery and controlled release.

**FIGURE 8:**
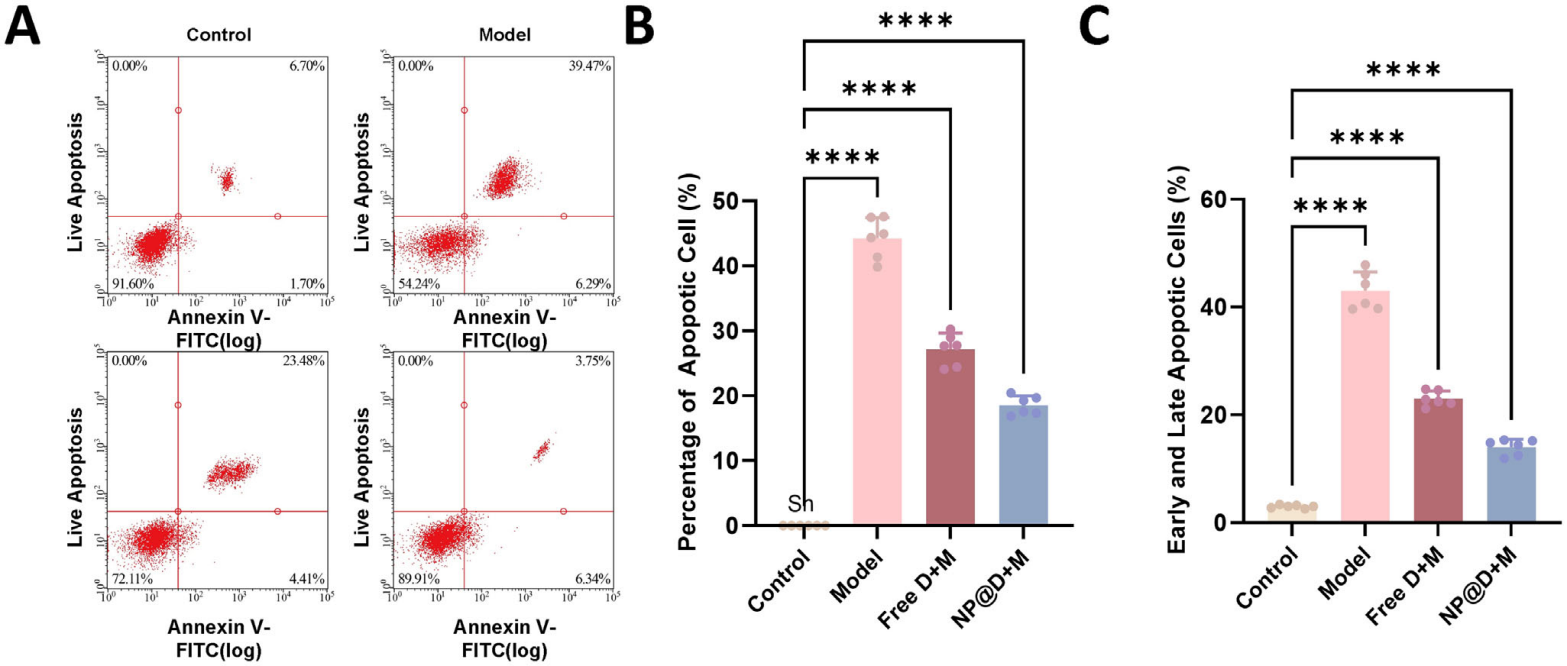
NP@D+M significantly reduces neuronal apoptosis in the ICH model. Note: (A) Annexin V‐FITC/PI flow cytometry scatter plots showing apoptotic cell distribution in each group. The upper right quadrant indicates late apoptosis, and the lower right quadrant indicates early apoptosis. The NP@D+M group exhibited a significantly lower proportion of apoptotic cells. (B) Quantitative analysis of total apoptosis across groups. (C) Bar graph showing early and late apoptosis proportions: the NP@D+M group significantly outperformed the free drug group in reducing both stages of apoptosis. n = 6, ^****^ *p* < 0.0001.

Moreover, scatter plots from Annexin V/PI staining revealed a significant increase in both early (Annexin V^+^/PI^–^) and late (Annexin V^+^/PI^+^) apoptotic populations in the Model group, which were substantially reduced following NP@D+M treatment (Figure 8A). Quantitative analyses further confirmed the enhanced anti‐apoptotic efficacy of NP@D+M compared to other groups (Figure 8B,C).

Collectively, these findings demonstrate that the biomimetic NP@D+M nanoparticle system not only mitigates oxidative stress but also markedly attenuates neuronal apoptosis, underscoring its promising potential as a neuroprotective strategy in ICH.

### 3.9 Inhibition of mPTP Opening

Based on the successful disruption of aberrant LPAR1–VDAC1 coupling, we further evaluated the effect of this upstream event on the opening dynamics of the mitochondrial permeability transition pore, its direct downstream effector. To investigate the protective effect of NP@D+M on mitochondrial dysfunction, we further assessed mPTP opening rates across experimental groups (Figure 9). The degree of mPTP opening was dynamically monitored using a colorimetric assay, which measured the decline in absorbance at 540 nm (A540) as an indicator of mitochondrial membrane permeability. In the Control group, mPTP remained minimally open, with an A540 decrease rate of 0.06 ± 0.01 A540/min, reflecting intact mitochondrial membrane integrity. In contrast, neurons in the Model group exhibited a significantly elevated mPTP opening rate of 0.28 ± 0.03 A540/min (*p* < 0.001 vs. Control), indicating marked disruption of mitochondrial membrane stability. Treatment with free DIDS and Mdivi‐1 partially mitigated mPTP opening, reducing the decline rate to 0.19 ± 0.02 A540/min (*p* < 0.01 vs. Model). Notably, NP@D+M treatment further attenuated mPTP opening, lowering the rate to 0.12 ± 0.02 A540/min (*p* < 0.001 vs. Model), approaching baseline levels.

**FIGURE 9:**
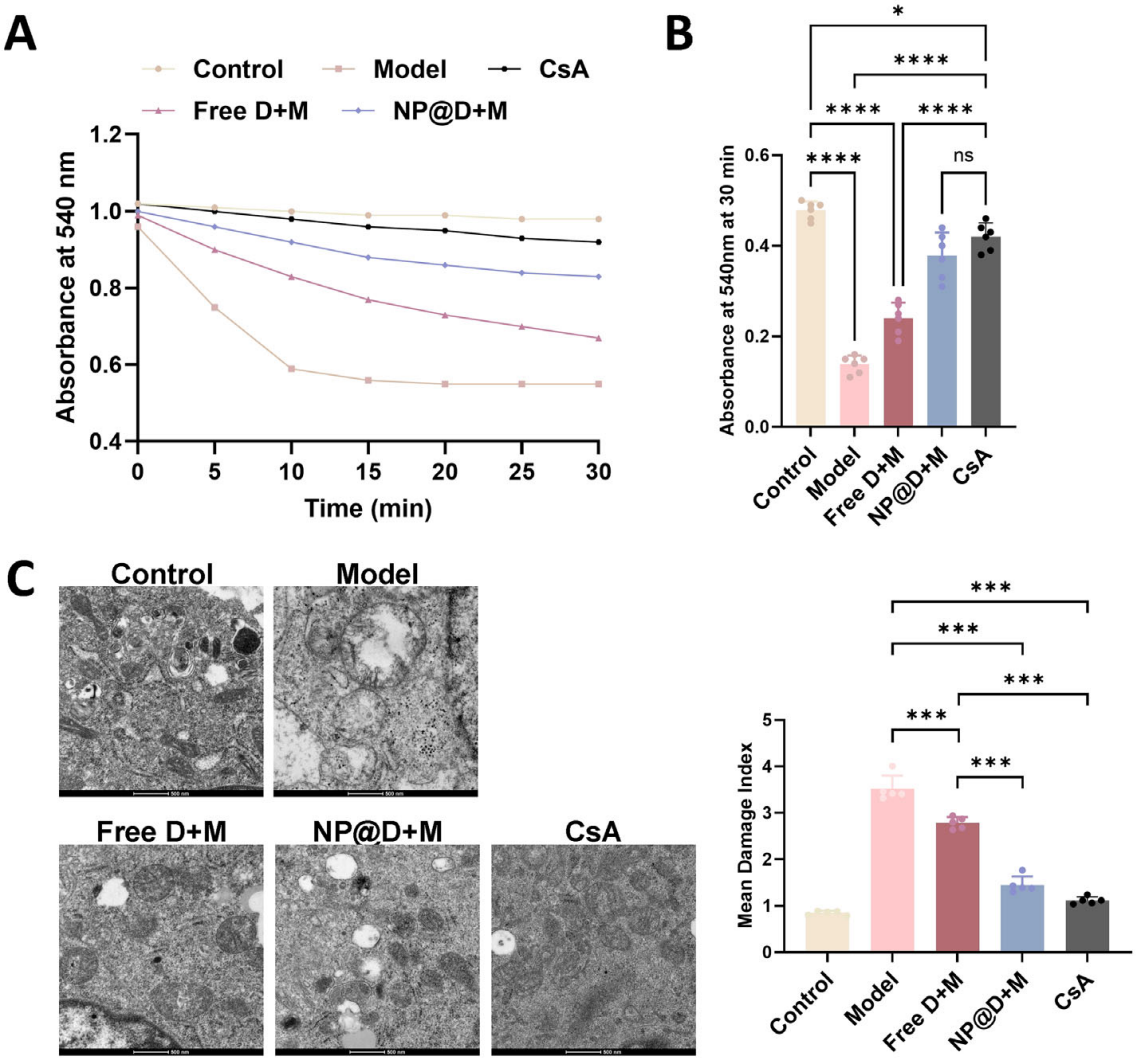
NP@D+M significantly inhibits mPTP opening in ICH model neurons. Note: (A) mPTP opening kinetics curves showing changes in absorbance at A540 over time in mitochondrial suspensions. The Model group demonstrated the fastest decline, while the NP@D+M group exhibited a markedly slower rate. (B) Bar graph of endpoint A540 absorbance: the NP@D+M group showed values approaching those of the Control group. (C) Representative TEM images of mitochondrial ultrastructure: the ICH+NS group displayed evident swelling and membrane rupture, whereas the NP@D+M group preserved intact morphology with well‐defined cristae (scale bar: 500 nm); quantitative results of the modified Flameng scoring system, showing that the mitochondrial damage index was significantly lower in both the NP@D+M and CsA groups than in the Model group. n = 50 mitochondria from 3 independent experiments; ns indicates no statistical significance, ^*^ *p* < 0.05, ^***^ *p* < 0.001, ^****^ *p* < 0.0001.

To visualize mitochondrial recovery more intuitively, we plotted mPTP kinetic curves and terminal A540 values. Kinetic curve analysis showed that mitochondrial absorbance remained stable in the Control group, whereas it rapidly decreased over time in the Model group, indicating extensive mPTP opening. The positive control CsA group markedly inhibited the decline in absorbance, confirming the specificity of this assay. The free drug group showed only limited inhibition of mPTP opening, whereas NP@D+M treatment significantly slowed the rate of absorbance decline and produced a flatter curve. The endpoint absorbance value in the NP@D+M group was significantly higher than that in the Model group (*p* < 0.001) and was comparable to that in the CsA group (Figure 9A,B). TEM further confirmed these findings at the morphological level (Figure 9C). In addition, mitochondrial ultrastructural injury was quantitatively evaluated using the modified Flameng scoring system. The results showed that the mean damage index (MDI) was markedly increased in the Model group, reaching 3.2 ± 0.3, whereas NP@D+M treatment reduced the MDI to 1.1 ± 0.2 (*p* < 0.001 vs. Model). The MDI in the CsA group was 0.9 ± 0.2, which was comparable to that in the NP@D+M group (*p* >0.05) and significantly lower than that in the free drug group (1.8 ± 0.3, *p* < 0.01). These quantitative results were highly consistent with the representative TEM images, objectively confirming the protective effect of NP@D+M on mitochondrial ultrastructure.

Collectively, these results demonstrate that the biomimetic, NP@D+M nanoparticle platform effectively suppresses pathological mPTP opening, thereby preserving mitochondrial homeostasis in neurons. This mechanistic insight highlights its potential in mitigating secondary injury following ICH. This effective inhibition of mitochondrial permeability transition pore opening and preservation of mitochondrial ultrastructural integrity not only prevented the apoptotic cascade but also provided an organelle‐level structural basis for the subsequent comprehensive recovery of mitochondrial energy metabolism.

### 3.10 siRNA‐Mediated Silencing or oeRNA Overexpression Validation and Functional Blockade

To elucidate the role of LPAR1 in the mitochondrial protective effects of NP@D+M, siRNA interference or oeRNA overexpression experiments were conducted. Quantitative PCR analysis revealed that transfection with siLPAR1 significantly reduced LPAR1 mRNA expression in neurons to 19.2% ± 2.3% of control levels (*p* < 0.001), while the expression level was significantly increased in the oeLPAR1 group (*p* < 0.001) (Figure 10A). Consistently, WB analysis demonstrated an over 80% decrease in LPAR1 protein expression in the siLPAR1+NP@D+M group relative to the untreated group (*p* < 0.001), confirming successful knockdown efficiency. In the oeLPAR1+NP@D+M group, neuronal LPAR1 protein expression was significantly higher than that in the control group, confirming successful target overexpression (Figure 10B).

**FIGURE 10:**
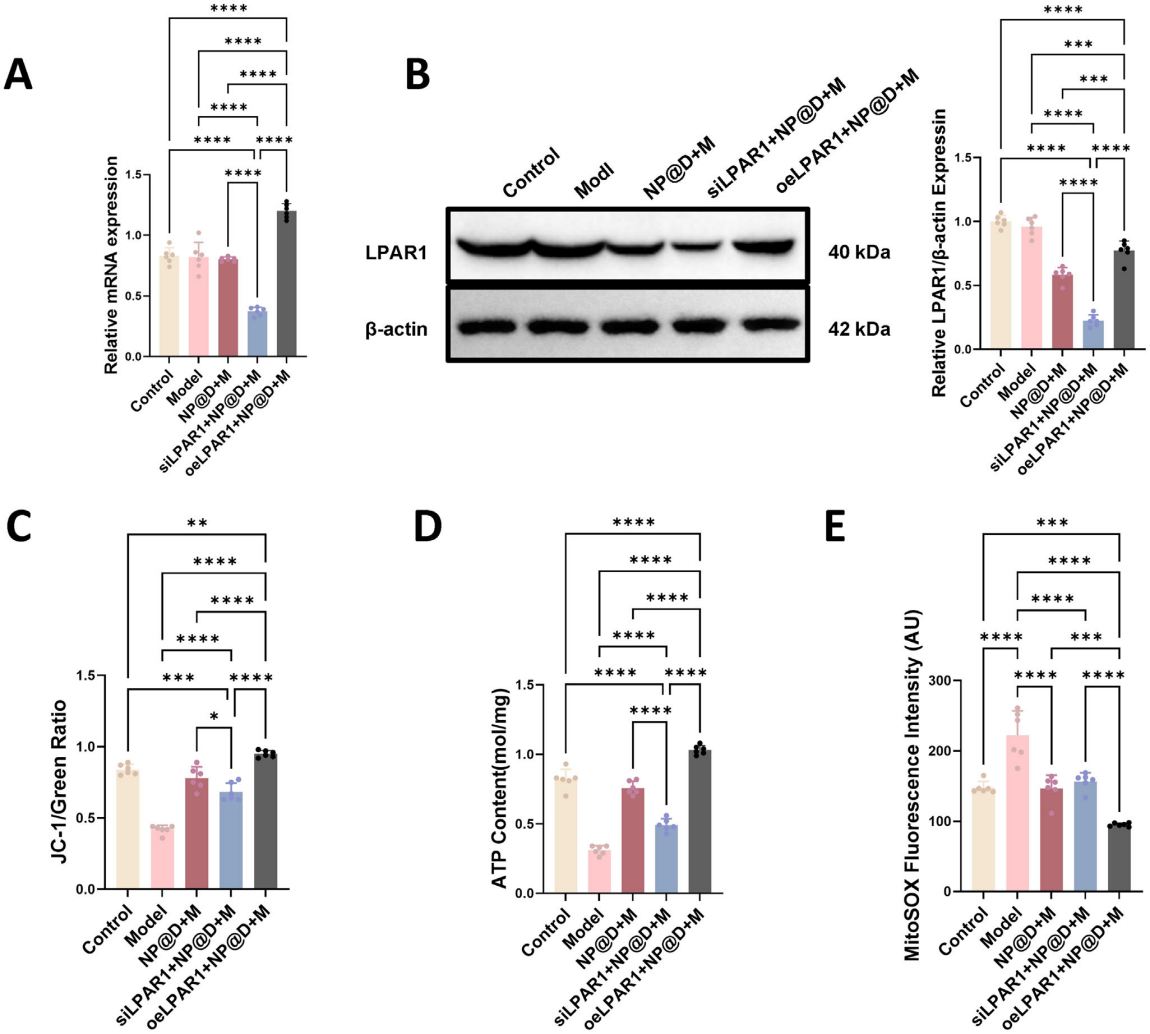
siRNA‐mediated silencing or overexpression of LPAR1 significantly regulates the mitochondrial protective effects of NP@D+M. Note: (A) qPCR analysis of LPAR1 mRNA expression in neurons shows approximately 80% reduction in the siLPAR1 group, and elevation in the oeLPAR1 group; (B) WB confirms changes in LPAR1 protein levels, with β‐actin as the loading control; (C) JC‐1 red/green fluorescence ratio; (D) ATP assay reveals significantly decreased ATP levels in the siLPAR1 group, and increased ATP levels in the oeLPAR1 group; (E) Quantification of MitoSOX fluorescence shows markedly increased ROS levels in the siLPAR1 group, and reduced ROS levels in the oeLPAR1 group. n=6, ^*^ *p* < 0.05, ^**^ *p* < 0.01, ^***^ *p* < 0.001, ^****^ *p* < 0.0001.

Functionally, LPAR1 silencing markedly attenuated the ability of NP@D+M to restore ΔΨm. JC‐1 staining showed that the red/green fluorescence ratio dropped to 0.55 ± 0.06 in the siLPAR1 group, significantly lower than the 0.83 ± 0.04 observed in the unsilenced NP@D+M group (*p* < 0.01), indicating reduced mitochondrial polarization. After oeLPAR1 intervention, the red/green fluorescence ratio increased and was significantly higher than that in the NP@D+M group without overexpression (*p* < 0.01). (Figure 10C). Moreover, intracellular ATP levels decreased by approximately 30% following LPAR1 knockdown, falling to 0.68 ± 0.05 nmol/mg compared to 0.96 ± 0.07 nmol/mg in the NP@D+M group (*p* < 0.01). ATP levels were significantly increased in the oeLPAR1 group by approximately 25% compared with the non‐silenced NP@D+M group (*p* < 0.01) (Figure 10D).

Mitochondrial oxidative stress also exhibited a rebound following siLPAR1 intervention, as evidenced by an increase in MitoSOX fluorescence intensity to 176 ± 14 AU, significantly higher than that of the NP@D+M group (125 ± 12 AU, *p* < 0.05). After oeLPAR1 intervention, mitochondrial ROS levels decreased, as reflected by reduced MitoSOX fluorescence intensity (*p* < 0.05) (Figure 10E). Collectively, these findings indicate that LPAR1 plays a pivotal regulatory role in NP@D+M‐mediated mitochondrial protection and energy metabolism, and its silencing significantly compromises the platform's therapeutic efficacy.

### 3.11 Co‐IP Confirms LPAR1–VDAC1 Interaction

To further investigate the molecular basis of NP@D+M‐mediated mitochondrial regulation, Co‐IP assays were performed to assess the interaction between VDAC1 and LPAR1 in neuronal samples (Figure 11). WB analysis showed low baseline expression of the LPAR1–VDAC1 complex in the Control group (normalized intensity: 1.00 ± 0.07). Upon LPS/Hb‐induced ICH modeling, the co‐precipitation signal significantly increased to 1.85 ± 0.12 (*p* < 0.001), suggesting enhanced formation of the LPAR1–VDAC1 complex, potentially contributing to mPTP activation.

**FIGURE 11:**
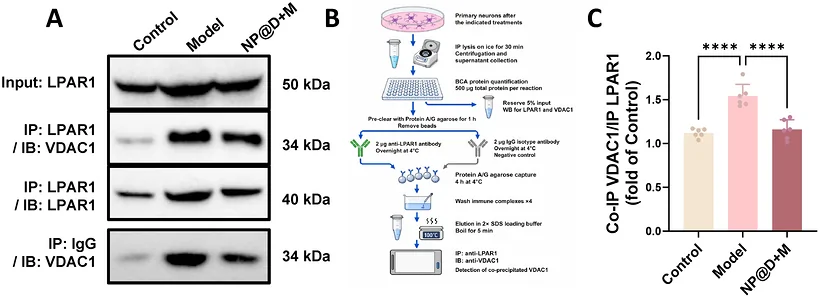
NP@D+M treatment significantly disrupts the formation of the LPAR1–VDAC1 protein complex in Neurons under ICH conditions. Note: (A) Co‐IP and WB analysis show relative abundance of the LPAR1–VDAC1 complex (top) and input control (bottom); the Control group exhibits weak complex formation, which is markedly increased in the Model group but significantly reduced following NP@D+M treatment; (B) Schematic illustration of the Co‐IP procedure: VDAC1 is co‐precipitated using an anti‐LPAR1 antibody and subsequently detected by WB; (C) Quantitative grayscale analysis indicates significantly lower complex formation in the NP@D+M group compared to the Model group. n=6, ^****^ *p* < 0.0001.

Treatment with NP@D+M markedly reduced the interaction between LPAR1 and VDAC1, lowering the co‐IP signal to 1.10 ± 0.08 (*p* < 0.001 vs. Model), approaching baseline levels observed in the Control group. This indicates that the biomimetic nanoparticle platform effectively disrupts aberrant LPAR1–VDAC1 binding. WB analysis of input lysates confirmed comparable total protein expression across groups (*p* > 0.05), ruling out potential loading bias (Figure 11A,B).

Densitometric quantification further supported these findings, demonstrating that NP@D+M significantly inhibits LPAR1–VDAC1 complex formation (*p* < 0.001), suggesting a mechanistic link to the stabilization of ΔΨm (Figure 11C).

### 3.12 Reduced Protein Colocalization

To elucidate the regulatory effect of NP@D+M on the spatial distribution and interaction between LPAR1 and VDAC1, a combination of immunofluorescence colocalization imaging, Pearson correlation analysis, 3D reconstruction, and line‐scan fluorescence profiling was employed (Figure 12). In the Control group, LPAR1 was predominantly localized to the plasma membrane and perinuclear cytoplasm, while VDAC1 exhibited a uniform distribution along the mitochondrial network. Minimal overlap was observed between the red and green fluorescence signals, with a low Pearson correlation coefficient of 0.35 ± 0.02, indicating limited basal colocalization under physiological conditions.

**FIGURE 12:**
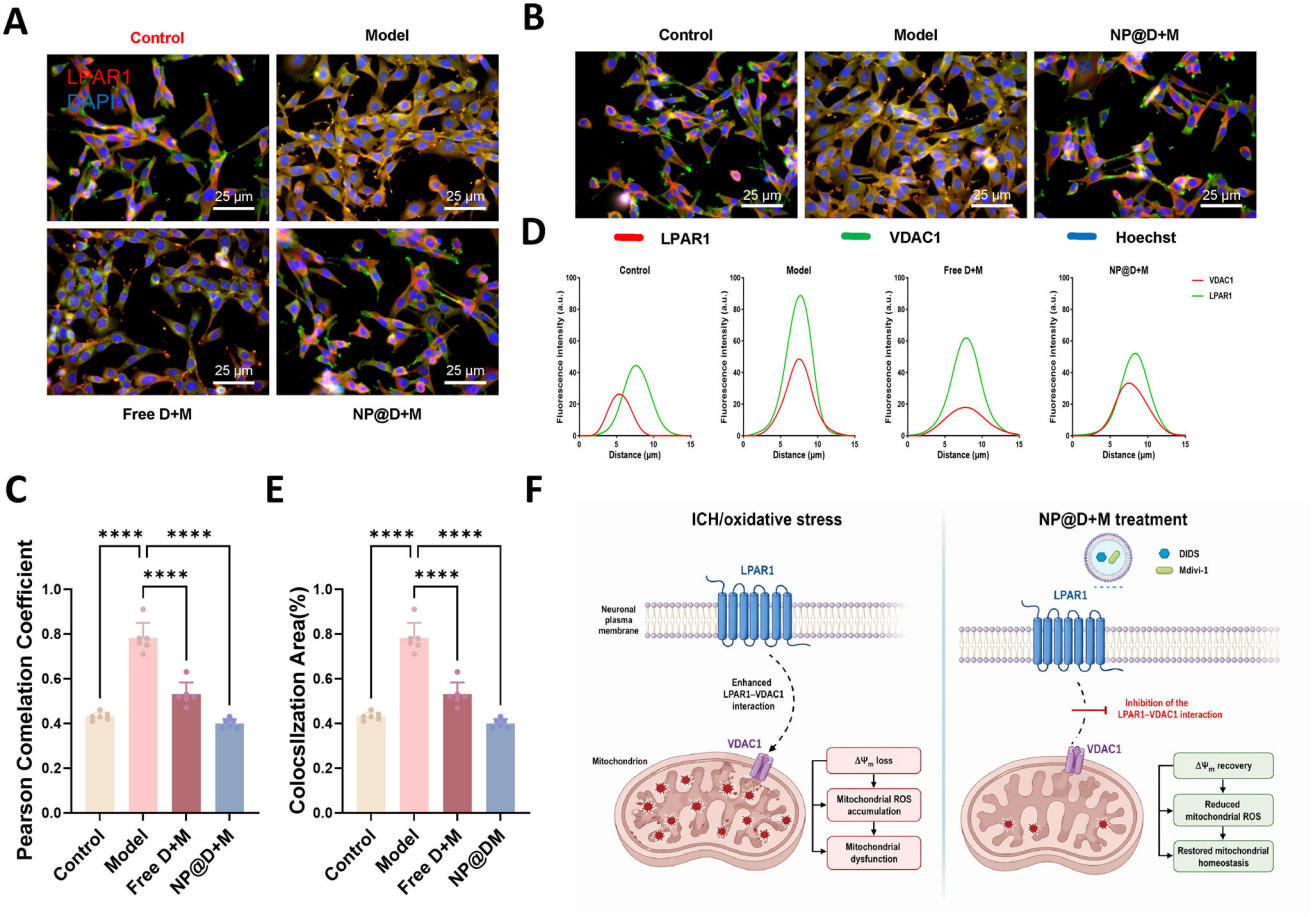
NP@D+M Significantly reduces the spatial colocalization of LPAR1 and VDAC1 in neurons following ICH. Note: (A) Representative immunofluorescence images showing the distribution and colocalization of LPAR1 (red) and VDAC1 (green) in neurons from different groups. Nuclei were counterstained with Hoechst (blue); scale bar, 25 µm. (B) 3D reconstruction images reveal dense, clustered colocalization in the Model group, which is markedly dispersed in the NP@D+M group. (C) Quantification of colocalization using Pearson's correlation coefficient demonstrates a significant reduction in the NP@D+M group. (D) Line‐scan analysis of fluorescence intensity indicates spatial separation of signal peaks. (E) Quantitative analysis of the colocalized area percentage further confirms the intervention effect. (F) Schematic illustration depicting how NP@D+M restores mitochondrial homeostasis and reduces oxidative stress by inhibiting the LPAR1–VDAC1 interaction. ^****^ *p* < 0.0001.

In contrast, the Model group demonstrated a marked increase in LPAR1–VDAC1 colocalization. Extensive signal overlap was detected in both the cytoplasm and neurites, with the Pearson coefficient rising to 0.82 ± 0.03 (*p* < 0.001), suggesting robust complex formation following ICH induction. This was further supported by synchronous fluorescence peaks in line‐scan analysis.

NP@D+M treatment markedly attenuated this abnormal colocalization. The overlapping regions between LPAR1 and VDAC1 were substantially reduced, with the Pearson coefficient decreasing to 0.42 ± 0.03 (*p* < 0.001 vs. Model), and the fluorescence peaks became clearly separated. Three‐dimensional reconstruction further revealed that the clustered colocalization patterns seen in the ICH+NS group were replaced by a more dispersed distribution after NP@D+M administration. Quantitative analysis showed significant reductions in both colocalization area and mean fluorescence overlap intensity (*p* < 0.001).

These results confirm that the nanoplatform can significantly disrupt the aberrant interaction between LPAR1 and VDAC1 under oxidative stress. As the starting point of the neuroprotective mechanistic pathway, the decoupling of this key membrane protein complex provides a direct molecular basis for subsequent investigation of downstream mitochondrial structural and functional protection.

### 3.13 NP@D+M Restores Glycolytic and TCA Cycle Metabolites and Improves Mitochondrial Energy Dysfunction

Given that VDAC1 activation and mPTP opening can directly impair mitochondrial oxidative phosphorylation and induce metabolic reprogramming, we further performed targeted metabolomic analysis to evaluate the metabolic status of glycolysis‐ and TCA cycle‐related pathways in neurons from each group (Figure 13). Targeted metabolomic analysis identified 13 differential metabolites (Figure 13A). Compared with the LPS/Hb‐induced ICH model group (ICH+NS), NP@D+M treatment significantly reduced the lactate/pyruvate ratio, suggesting alleviation of compensatory glycolysis and lactate accumulation (Figure 13B). In addition, the level of citrate, a key metabolite in the TCA cycle, was markedly restored after NP@D+M treatment (Figure 13C), indicating partial improvement of mitochondrial oxidative metabolism‐related abnormalities. Further PCA analysis showed that the overall metabolic profile of the Treatment group shifted toward that of the Control group (Figure 13D). OPLS‐DA and S‐plot analyses further confirmed that NP@D+M significantly corrected ICH‐induced metabolic disturbances (Figure 13E,F).

**FIGURE 13:**
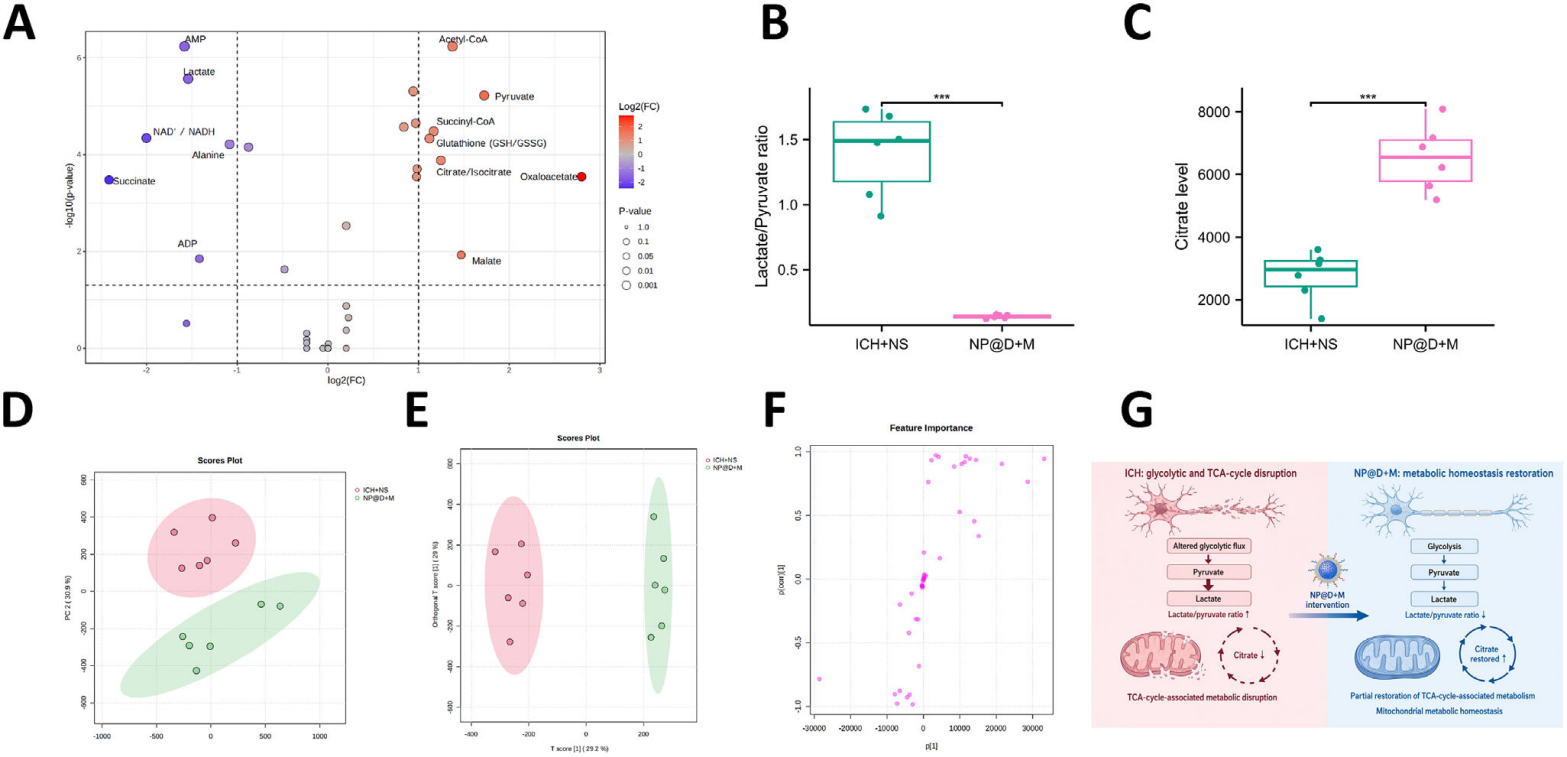
NP@D+M significantly restores Glycolytic and TCA cycle metabolic homeostasis in neurons following ICH. Note: (A) Volcano plot of differential metabolites from targeted metabolomic analysis; (B) Lactate/pyruvate ratio; (C) Citrate levels; (D) PCA plot of metabolomic data; (E) OPLS‐DA score plot; (F) S‐plot of metabolomic data; (G) Schematic illustration. Each group, n = 6.

Together with the previously observed ATP restoration, increased mitochondrial membrane potential, and reduced ROS levels, these data support that NP@D+M alleviates metabolic abnormalities associated with mitochondrial dysfunction (Figure 13G).

### 3.14 Single‐Cell Transcriptomics Reveals NP@D+M‐Mediated Reversal of ICH‐Associated Mitochondrial Injury and Inflammatory Cell States

To systematically dissect the regulatory effects of NP@D+M intervention on metabolic homeostasis in oligodendrocyte‐lineage cells, we performed scRNA‐seq (Figure 14). Based on marker gene annotation, we identified multiple cell populations, including smooth muscle cells (Acta2), endothelial cells (Cldn5, Kdr), monocytes (Ly6c2), oligodendrocyte precursor cells (Enpp2), microglia (Hexb), astrocytes (Aldoc), dendritic cells (Ccl17), fibroblasts (Lum, Col1a1), pericytes (Kcnj8), megakaryocytes (Pf4), neutrophils (S100a8), NK cells (NKG7), neural progenitors (Sox2), mature oligodendrocytes (Plp1), and a small fraction of erythroid cells (Hba‐a1) (Figure 14A,B). We further extracted DEGs (logFC > 1.2, FDR < 0.05) from oligodendrocytes (Figure 14C,D) and oligodendrocyte precursor cells (Figure 14E,F). Gene Ontology (GO) and KEGG pathway enrichment analyses revealed significant associations with processes such as glial cell apoptosis, mitochondrial alterations during apoptosis, neuronal cell death, and leukocyte migration.

**FIGURE 14:**
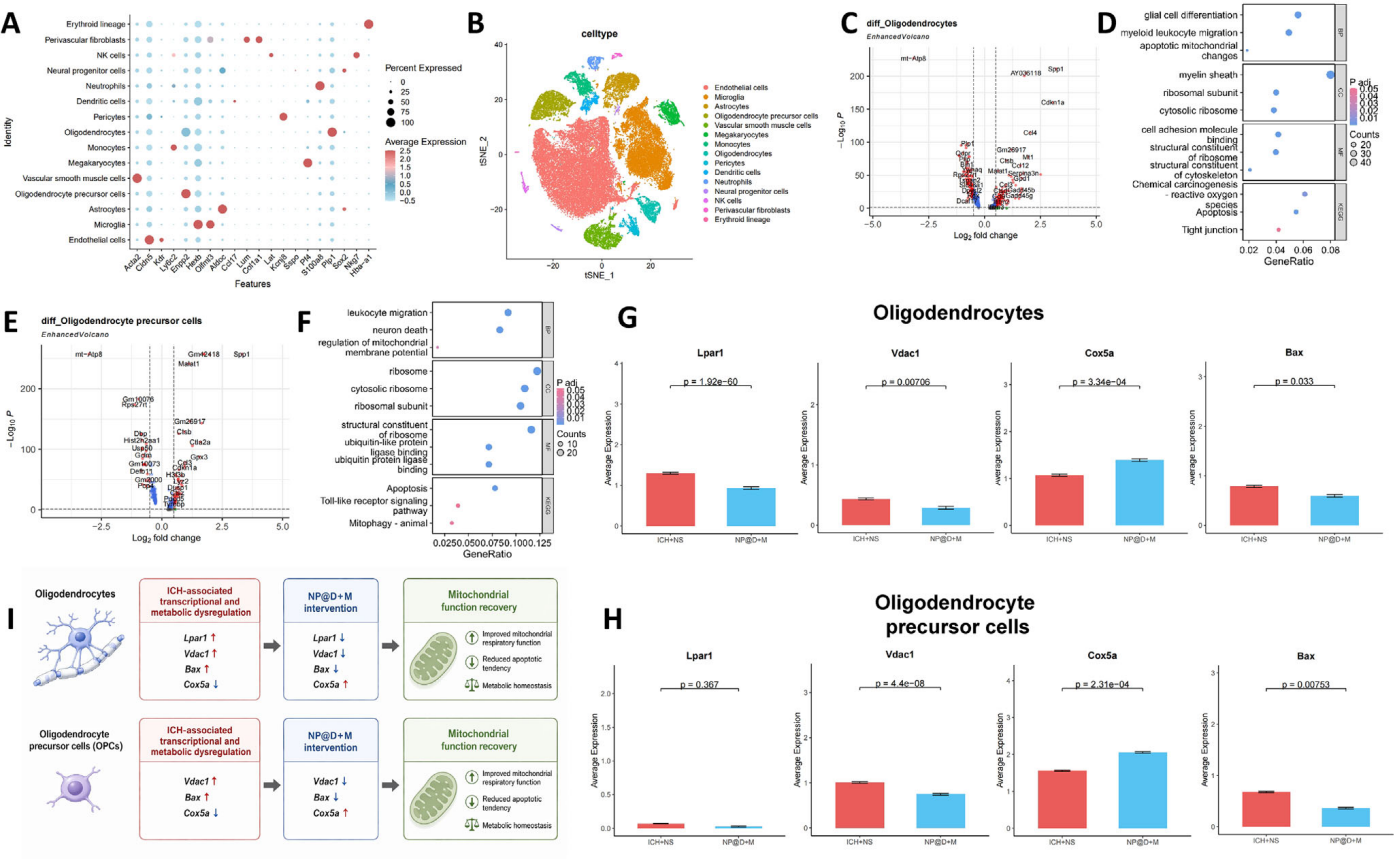
Single‐cell RNA‐seq reveals that NP@D+M markedly restores metabolic homeostasis in Oligodendrocytes and Oligodendrocyte precursor cells after ICH. Note: (A) Bubble plot showing marker gene expression across distinct cellular subpopulations. (B) Visualization of cell‐type annotation based on t‐SNE clustering of scRNA‐seq data. (C) Volcano plot of DEGs in oligodendrocytes. (D) Bubble plot of GO and KEGG enrichment analysis. (E) Volcano plot of DEGs in oligodendrocyte precursor cells. (F) Bubble plot of GO and KEGG enrichment analysis. (G, H) Changes in the expression of Lpar1, Vdac1, the pro‐apoptotic gene Bax, and the mitochondrial respiratory chain‐related gene Cox5a in oligodendrocytes and oligodendrocyte precursor cells. (I) Schematic illustration depicting how NP@D+M promotes the restoration of metabolic homeostasis and mitochondrial function through transcriptional regulation.

Further analysis of key mechanism‐related genes showed that, compared with the ICH + NS group, NP@D+M treatment significantly reduced the expression of Lpar1, Vdac1, and the pro‐apoptotic gene Bax in oligodendrocytes, while markedly increasing the expression of the mitochondrial respiratory chain‐related gene Cox5a (Figure 14G). Consistent changes were also observed in oligodendrocyte precursor cells (Figure 14H), suggesting that NP@D+M effectively alleviates ICH‐induced mitochondrial injury and apoptotic tendency in oligodendrocyte lineage cells and promotes the reconstruction of energy metabolic homeostasis.

Integrating the single‐cell transcriptomic evidence, these results clearly support the mechanism by which NP@D+M simultaneously regulates mitochondrial homeostasis and energy metabolism at the transcriptional level (Figure 14I).

### 3.15 Behavioral Improvements

To comprehensively evaluate the therapeutic impact of NP@D+M on neurological recovery and cognitive performance, a multi‐dimensional behavioral assessment was conducted using the Garcia neurological scoring system and the Morris water maze (Figure 15). The Garcia score encompasses six parameters, including spontaneous activity, limb symmetry, forelimb outstretching, and reflexes. In the ICH+NS group, the average total score dropped to 8.00 ± 0.30 compared with 15.98 ± 1.34 in the Sham group (*p* < 0.0001), indicating neurological impairment. Treatment with NP@D+M increased the total score to 9.00 ± 0.95 (*p* = 0.0484 vs. ICH+NS), suggesting a partial improvement in neurological function (Figure 15A). Representative behavioral clips are provided in Movie S1.

**FIGURE 15:**
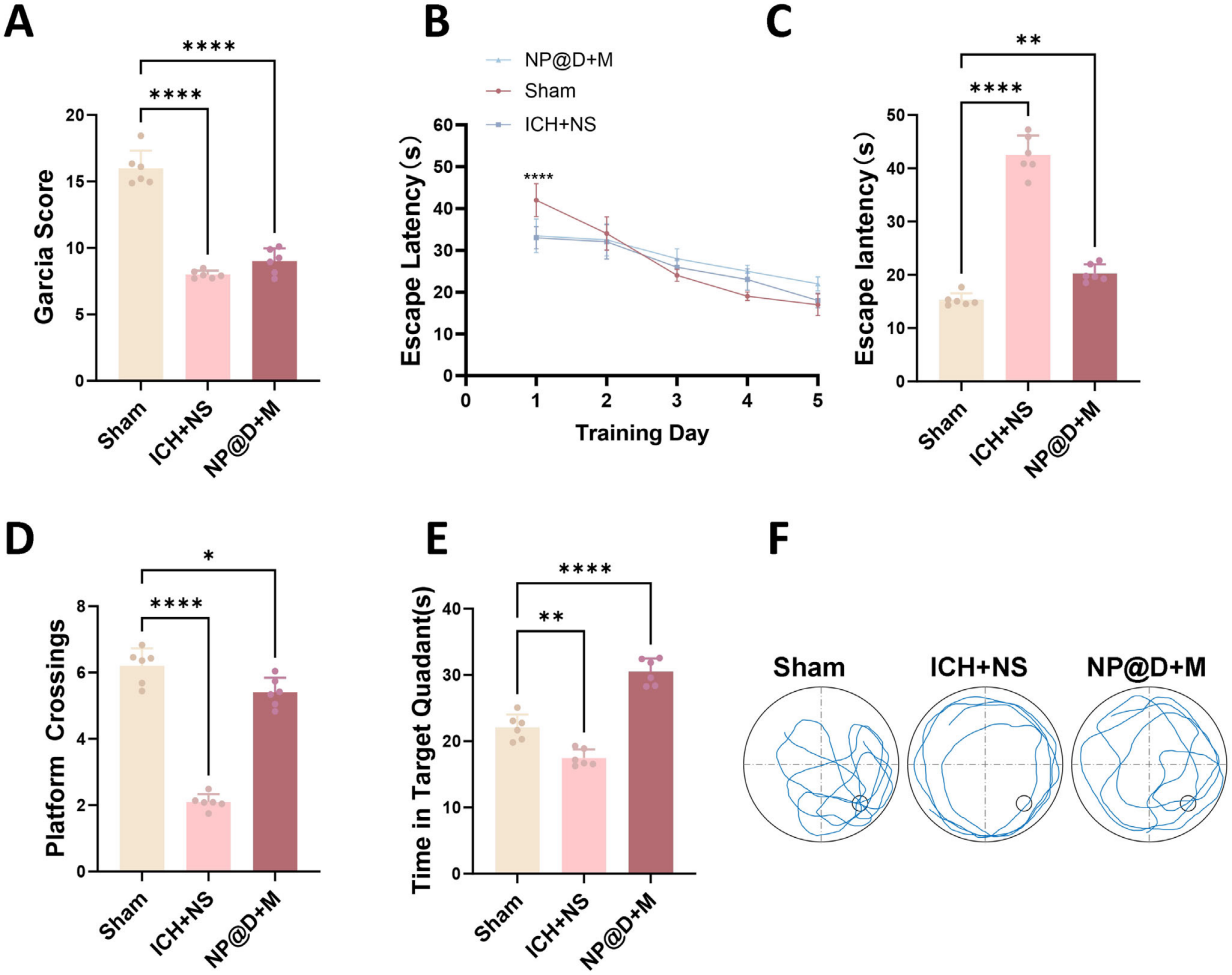
NP@D+M significantly improves neurological function and spatial learning ability in ICH mouse model. Note: (A) Bar graph of Garcia scores in the Sham, ICH+NS, and NP@D+M groups. (B) Morris water maze acquisition training curve showing escape latency over time in the three groups. (C) Endpoint escape‐latency summary showing a shorter latency in the NP@D+M group than in the ICH+NS group. (D) Platform crossing frequency during the probe test. (E) Time spent in the target quadrant during the probe test. (F) Representative spatial navigation trajectories for the three groups. ^*^ *p* < 0.05, ^**^ *p* < 0.01, ^****^ *p* < 0.0001.

Cognitive function was further assessed using the Morris water maze. During acquisition training, escape latency changed over time across the three groups (Figure 15B). In the endpoint escape‐latency summary, the ICH+NS group showed a prolonged latency of 42.50 ± 3.67 s compared with 15.29 ± 1.23 s in the Sham group (*p* < 0.0001; Figure 15C). NP@D+M treatment reduced the latency to 20.32 ± 1.65 s (*p* < 0.0001 vs. ICH+NS), indicating improved spatial learning performance. In the subsequent probe‐trial analyses, NP@D+M increased the number of platform crossings (5.40 ± 0.45 vs. 2.10 ± 0.21, *p* < 0.0001; Figure 15D) and the time spent in the target quadrant (30.51 ± 1.94 s vs. 17.48 ± 1.30 s, *p* < 0.0001; Figure 15E) compared with ICH+NS. Representative swim path heatmaps further illustrated group differences in spatial navigation strategies (Figure 15F). Representative water‐maze videos are provided in Movie S2.

Collectively, these findings demonstrate that the NP@D+M nanoplatform not only mitigates acute neurological deficits but also facilitates the recovery of spatial learning and memory, underscoring its multifaceted neuroprotective potential in the context of ICH.

### 3.16 Histological and Immunohistochemical Validation

To further substantiate the neuroprotective effects of NP@D+M against ICH‐induced neuronal damage, histological evaluation was performed using Nissl staining and immunohistochemical detection of cleaved caspase‐3 (Figure 16). Nissl staining revealed that neurons in the Sham group exhibited orderly arrangement and well‐defined nuclear morphology. In contrast, the ICH+NS group showed a marked reduction in viable neurons (30.2 ± 3.0 cells/field), along with nuclear pyknosis and dissolution, indicative of severe neuronal injury. Following NP@D+M treatment, neuronal density significantly increased to 62.8 ± 4.5 cells/field (*p* < 0.001), with preserved morphology comparable to controls, suggesting a robust neuroprotective effect (Figure 16A).

**FIGURE 16:**
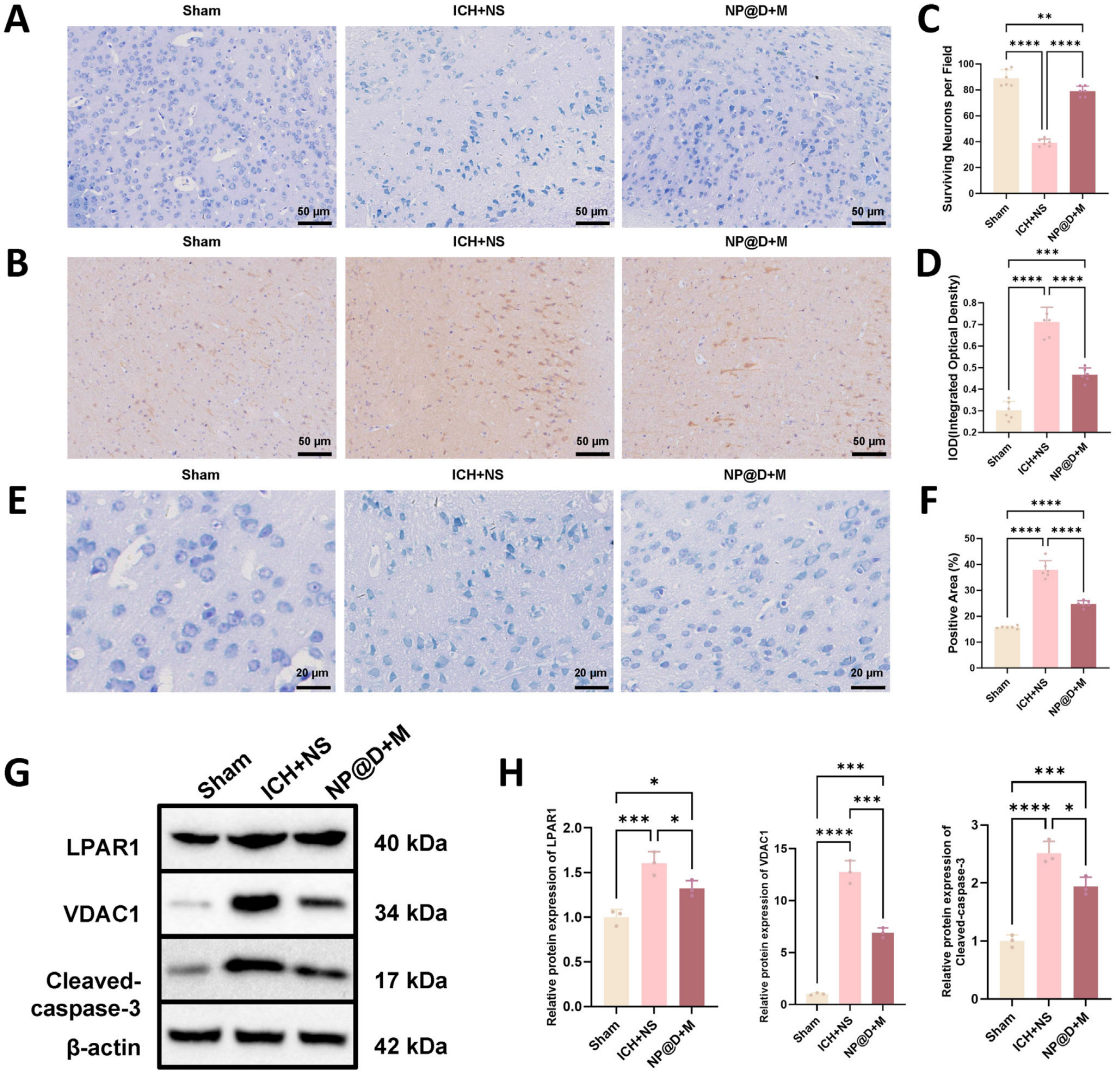
NP@D+M markedly improves neuronal survival and reduces apoptotic marker expression in ICH mouse model. Note: (A) Representative Nissl‐stained sections. The Sham group shows orderly arranged neurons, whereas the ICH+NS group exhibits significantly reduced neuronal count and signs of pyknosis. The NP@D+M group demonstrates notable restoration of surviving neurons. Scale bar = 50 µm. (B) Representative immunohistochemical staining of cleaved caspase‐3. Positive expression is markedly increased in the ICH+NS group and substantially reduced in the NP@D+M group. Scale bar = 50 µm. (C) Quantification of surviving neurons. The NP@D+M group shows a significantly higher neuron count compared to the ICH+NS group. (D) Bar graph of IOD of cleaved caspase‐3 staining. The NP@D+M group exhibits a significant reduction relative to the ICH+NS group. (E) High‐magnification image of Nissl‐positive cells illustrating morphological differences in neuronal nuclei. Scale bar = 50 µm. (F) Quantitative analysis of the cleaved caspase‐3‐positive staining area, indicating a substantial reduction in the NP@D+M group. (G) Western blot analysis of LPAR1, VDAC1, and cleaved caspase‐3 protein expression in perihematomal brain tissues of mice, with β‐actin used as the internal control. (H) Quantitative densitometric analysis of relative protein expression based on Western blot bands. Data are presented as mean ± SD. Compared with the ICH+NS group, NP@D+M treatment significantly reduced the expression of core mechanistic target proteins and the apoptosis marker. n = 3; ^*^ *p* < 0.05, ^**^ *p* < 0.01, ^***^ *p* < 0.001, ^****^ *p* < 0.0001.

To assess apoptosis, cleaved caspase‐3 expression was examined (Figure 16B–F). The ICH+NS group exhibited a substantial increase in both immunopositive area and IOD, reflecting enhanced apoptotic activation (*p* < 0.001). NP@D+M administration significantly reduced cleaved caspase‐3 expression, with IOD levels approximately 60% lower than in the ICH+NS group (*p* < 0.001), further supporting its anti‐apoptotic efficacy.

To further confirm the core molecular mechanism underlying the neuroprotective effect of NP@D+M at the in vivo tissue level, we extracted total protein from perihematomal brain tissues of mice in the Sham, ICH+NS, and NP@D+M groups while evaluating histological improvement. Western blot analysis was performed to detect the expression levels of LPAR1, VDAC1, and the apoptosis execution protein cleaved caspase‐3 (Figure 16G,H). The results showed that, compared with the Sham group, LPAR1 and VDAC1 protein expression was significantly upregulated in the brain tissues of the ICH+NS group, accompanied by marked activation of cleaved caspase‐3. These findings indicate that aberrant activation of the LPAR1/VDAC1 axis is closely associated with severe tissue cell apoptosis in the pathological environment of ICH in vivo. After NP@D+M intervention, the abnormal overexpression of LPAR1 and VDAC1 was significantly suppressed, and cleaved caspase‐3 levels were markedly reduced. These tissue‐level quantitative protein results were highly consistent with the phenotypic rescue observed in the preceding Nissl staining and immunohistochemical analyses. More importantly, they provide direct in vivo molecular evidence demonstrating that NP@D+M effectively inhibits neuronal programmed cell death and protects mitochondrial function by precisely regulating the LPAR1/VDAC1 axis in animals.

Together, these findings strongly confirm the tissue‐level neuroprotective effects of NP@D+M in the context of ICH.

### 3.17 In Vivo Restoration of Mitochondrial Function

To systematically evaluate the restorative impact of NP@D+M on mitochondrial function in brain tissue, JC‐1 staining was employed to measure ΔΨm (Figure 17). Fluorescence ratio analysis indicated that Δψm remained high in the Sham group (red/green ratio: 1.00 ± 0.06), whereas the ICH+NS group showed a significant decline to 0.38 ± 0.04 (*p* < 0.001), consistent with mitochondrial depolarization. NP@D+M treatment markedly restored this ratio to 0.80 ± 0.05 (*p* < 0.001) (Figure 17A,B). Biochemical quantification of ATP levels further demonstrated that ATP content dropped to 0.42 ± 0.06 nmol/mg protein in the ICH+NS group, less than half of the Sham group (1.05 ± 0.08 nmol/mg, *p* < 0.001). Notably, NP@D+M treatment restored ATP levels to 0.92 ± 0.07 nmol/mg (*p* < 0.001) (Figure 17C). A similar recovery trend was observed in the activities of mitochondrial respiratory chain complexes I and IV (Figure 17D,E). Additionally, MitoSOX Red staining revealed a significant reduction in mitochondrial superoxide accumulation following NP@D+M administration, indicating alleviated oxidative stress (Figure 17F,G). Collectively, these results demonstrate that NP@D+M effectively restores ΔΨm, enhances ATP production, and reestablishes redox homeostasis in the ICH model, highlighting its potential in mitigating mitochondrial dysfunction (Figure 17H).

**FIGURE 17:**
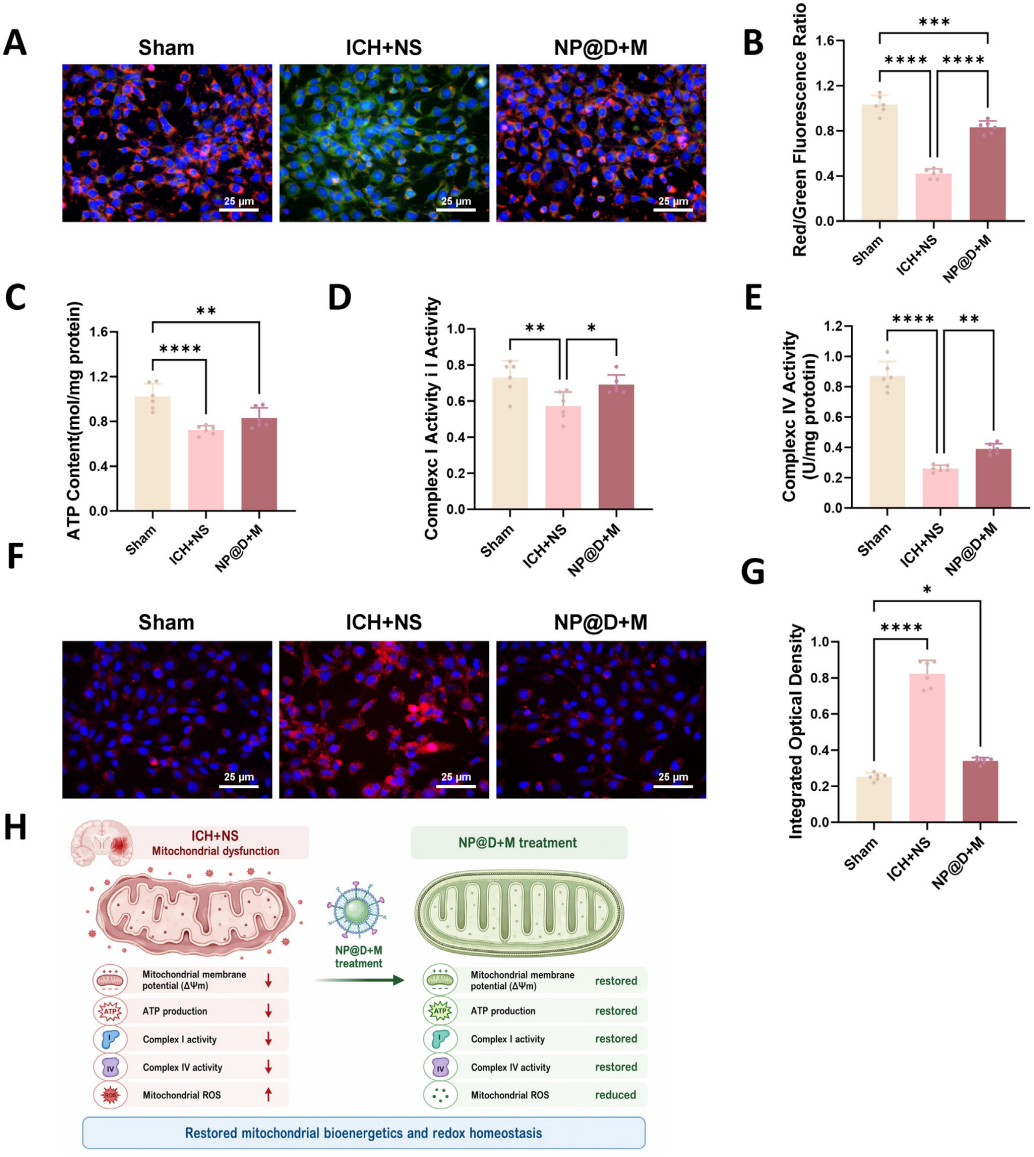
NP@D+M significantly restores ΔΨm and bioenergetic function in ICH mouse model. Note: (A) Representative JC‐1 fluorescence microscopy images. The Sham group exhibits a high red/green fluorescence ratio, indicating intact ΔΨm; the ICH+NS group shows significant depolarization (increased green fluorescence), which is notably reversed in the NP@D+M group. (B) Quantification of JC‐1 red/green fluorescence ratio. A significant decrease is observed in the ICH+NS group, while the NP@D+M group shows a marked increase. (C) Bar graph of ATP content. ATP levels are significantly reduced in the ICH+NS group but are restored to near‐Sham levels in the NP@D+M group. (D) Bar graph showing mitochondrial respiratory chain Complex I activity. The NP@D+M group demonstrates restored activity. (E) Bar graph showing mitochondrial respiratory chain Complex IV activity. A significant increase is observed in the NP@D+M group. (F) Representative MitoSOX fluorescence images indicating mitochondrial oxidative stress. Red fluorescence intensity is highest in the ICH+NS group and significantly reduced in the NP@D+M group. (G) Quantitative analysis of MitoSOX fluorescence integrated density, showing significantly decreased oxidative stress in the NP@D+M group. (H) Schematic illustration demonstrating that NP@D+M mitigates oxidative stress and improves energy metabolism by restoring ΔΨm and ATP production. ^*^ *p* < 0.05, ^**^ *p* < 0.01, ^***^ *p* < 0.001, ^****^ *p* < 0.0001.

### 3.18 In Vivo Biodistribution of Nanoparticles

DiR‐labeled nanoparticles were serially monitored in the same mice at 2, 6, 12, and 24 h after intravenous injection. Brain fluorescence in the NP@D+M group progressively increased over the observation period and reached its highest measured value at 24 h, with an average radiant efficiency of 5.2 ± 0.4, compared with 3.1 ± 0.3 in the uncoated nanoparticle group (Figure 18A–C and Table 1). Immediately after the 24‐h imaging session, mice were euthanized for terminal ex vivo organ imaging. NP@D+M showed greater fluorescence accumulation in the brain than the uncoated nanoparticles, whereas the liver exhibited the strongest major‐organ clearance‐associated signal (Figure 18D). The brain‐to‐liver fluorescence ratio was higher in the NP@D+M group than in the uncoated nanoparticle group (Figure 18E). Brain coronal‐section and confocal analyses performed at 24 h further demonstrated increased parenchymal distribution of NP@D+M (Figure 18F,G), with the overall imaging workflow shown in Figure 18H.

**FIGURE 18:**
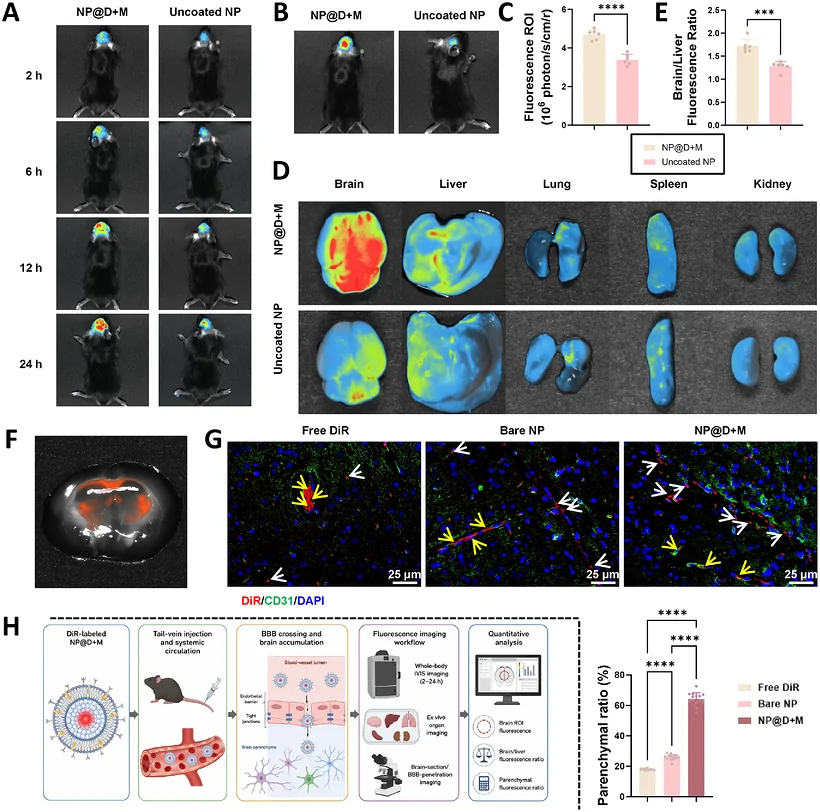
In vivo fluorescence distribution and brain‐targeting efficiency of NP@D+M. Note: (A) Serial IVIS fluorescence images obtained from the same mice at 2, 6, 12, and 24 h after intravenous injection. (B) Representative whole‐body fluorescence images at 24 h. (C) Quantitative analysis of brain ROI fluorescence over 2–24 h. (D) Terminal ex vivo fluorescence imaging of the brain, liver, lungs, kidneys, and spleen collected immediately after the 24‐h imaging session. (E) Brain‐to‐liver fluorescence ratio at 24 h. (F) Near‐infrared fluorescence imaging of brain coronal sections collected at 24 h; scale bar, 1 mm. (G) Confocal analysis of intravascular and extravascular nanoparticle distribution in brain tissue collected at 24 h. Red indicates DiR‐labeled nanoparticles, green indicates CD31‐positive cerebral microvessels, and blue indicates DAPI‐stained nuclei. Yellow arrows indicate intravascular nanoparticles, whereas white arrows indicate nanoparticles located in the extravascular brain parenchyma ≥2 µm outside the CD31‐positive vascular structures. The parenchymal ratio was quantified from 15–20 vessels obtained from three mice; scale bar, 25 µm. (H) Schematic illustration of the serial in vivo imaging, terminal ex vivo organ imaging, and brain fluorescence quantification workflow.

**TABLE 1:** Quantitative analysis of in vivo brain fluorescence and terminal ex vivo organ fluorescence following administration of NP@D+M or uncoated nanoparticles.

A. Serial in vivo brain ROI fluorescence (average radiant efficiency [(photons/s/cm^2^/sr)/(µW/cm^2^)])				
**Group**	**2 h**	**6 h**	**12 h**	**24 h**
NP@D+M	1.9 ± 0.2	3.2 ± 0.3	4.5 ± 0.3	5.2 ± 0.4
Uncoated NP	1.0 ± 0.1	2.0 ± 0.2	2.7 ± 0.3	3.1 ± 0.3
**B. Terminal ex vivo organ fluorescence at 24 h (average radiant efficiency [(photons/s/cm^2^/sr)/(µW/cm^2^)])**				
**Tissue**	**NP@D+M at 24 h**		**Uncoated NP at 24 h**	
Brain	5.2 ± 0.4		3.1 ± 0.3	
Liver	4.0 ± 0.3		4.5 ± 0.4	
Lung	1.9 ± 0.2		2.0 ± 0.2	
Kidney	1.1 ± 0.1		1.2 ± 0.2	
Spleen	0.7 ± 0.1		0.8 ± 0.1	

Collectively, these results demonstrate that the biomimetic nanoplatform with H_2_O_2_‐dependent drug‐release behavior possesses excellent brain‐targeting efficiency and BBB penetration capability, thereby establishing a robust foundation for subsequent mitochondrial restoration therapies.

### 3.19 Mechanistic Validation via siLPAR1 Rescue Experiment

To validate the contribution of LPAR1 to the therapeutic effects of NP@D+M, mice received either non‐targeting siRNA (siRNA NC) or siLPAR1 12 h before ICH induction, followed by NP@D+M administration at 1 and 24 h after ICH induction (Figure 19A and Table 2). qRT‐PCR analysis confirmed that LPAR1 mRNA expression was markedly reduced in the ICH+NP@D+M+siLPAR1 group compared with the ICH+NP@D+M+siRNA NC group (31.24 ± 3.05 vs. 102.63 ± 15.85, *p* < 0.0001; Figure 19B). Western blotting further confirmed LPAR1 protein knockdown (Figure 19C).

**FIGURE 19:**
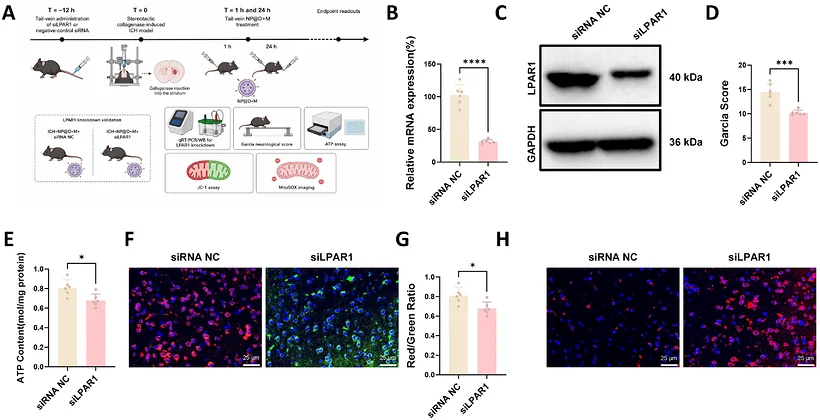
LPAR1 knockdown attenuates the neuroprotective and mitochondrial effects of NP@D+M. Note: (A) Experimental workflow. siLPAR1 or non‐targeting siRNA (siRNA NC) was administered by tail‐vein injection 12 h before ICH induction, followed by NP@D+M administration at 1 and 24 h after ICH induction. (B) qRT‐PCR analysis of LPAR1 mRNA expression. (C) Western blot analysis confirming LPAR1 knockdown, with GAPDH used as the loading control. (D) Garcia neurological scores. (E) ATP content in brain tissue. (F) Representative JC‐1 fluorescence images; scale bar, 25 µm. (G) Quantification of the JC‐1 red/green fluorescence ratio. (H) Representative MitoSOX fluorescence images; scale bar, 25 µm. Data are presented as mean ± SD; n = 6 per group. ^*^ *p* < 0.05, ^**^ *p* < 0.01, ^****^ *p* < 0.0001.

**TABLE 2:** Effects of LPAR1 knockdown on neurological and mitochondrial function in NP@D+M‐treated ICH mice.

Indicator	ICH+NP@D+M+siRNA NC	ICH+NP@D+M+siLPAR1	*p* value
Garcia score	14.52 ± 1.82	10.21 ± 0.54	0.0016
ATP content (nmol/mg protein)	0.81 ± 0.08	0.68 ± 0.07	0.0118
JC‐1 red/green fluorescence ratio	0.60 ± 0.05	0.38 ± 0.04	<0.0001

Note: Data are presented as mean ± standard deviation (n = 6). The Garcia score reflects overall neurological function with a maximum of 18 points. The JC‐1 red/green fluorescence ratio serves as an indicator of mitochondrial membrane potential, where lower values denote increased depolarization. Knockdown of LPAR1 attenuated the therapeutic effects of NP@D+M.

Compared with the ICH+NP@D+M+siRNA NC group, the ICH+NP@D+M+siLPAR1 group showed lower Garcia neurological scores (10.21 ± 0.54 vs. 14.52 ± 1.82, *p* = 0.0016; Figure 19D), decreased ATP content (0.68 ± 0.07 vs. 0.81 ± 0.08 nmol/mg protein, *p* = 0.0118; Figure 19E), and a reduced JC‐1 red/green fluorescence ratio (0.38 ± 0.04 vs. 0.60 ± 0.05, *p* < 0.0001; Figure 19F,G). Representative MitoSOX fluorescence images showed increased mitochondrial ROS after LPAR1 knockdown (Figure 19H). These findings indicate that LPAR1 knockdown attenuated the neurological and mitochondrial protective effects of NP@D+M.

Thus, by targeted silencing of LPAR1, we comprehensively validated the mechanistic cascade linking regulation of the LPAR1–VDAC1 axis to mPTP closure, restoration of mitochondrial function, and ultimately improvement of neurological recovery. These findings indicate that LPAR1 is not only a key target of the nanoplatform but also a central hub connecting local mitochondrial homeostasis with overall neuronal survival.

### 3.20 Favorable Safety Profile

To comprehensively evaluate the in vivo safety of repeated NP@D+M administration, both serum biochemistry and histopathological assessments of major organs were conducted (Figure 20A–F). Serum biochemical analyses revealed no significant differences between Control and NP@D+M groups in ALT (31.2 ± 4.3 vs. 32.4 ± 4.7 U/L), AST (65.9 ± 5.1 vs. 67.8 ± 5.5 U/L), BUN (7.3 ± 0.7 vs. 7.5 ± 0.8 mmol/L), or creatinine (37.6 ± 3.9 vs. 38.2 ± 4.2 µmol/L) levels (all *p* > 0.05; Figure 20A–D; Table 3), indicating preserved hepatic and renal function.

**FIGURE 20:**
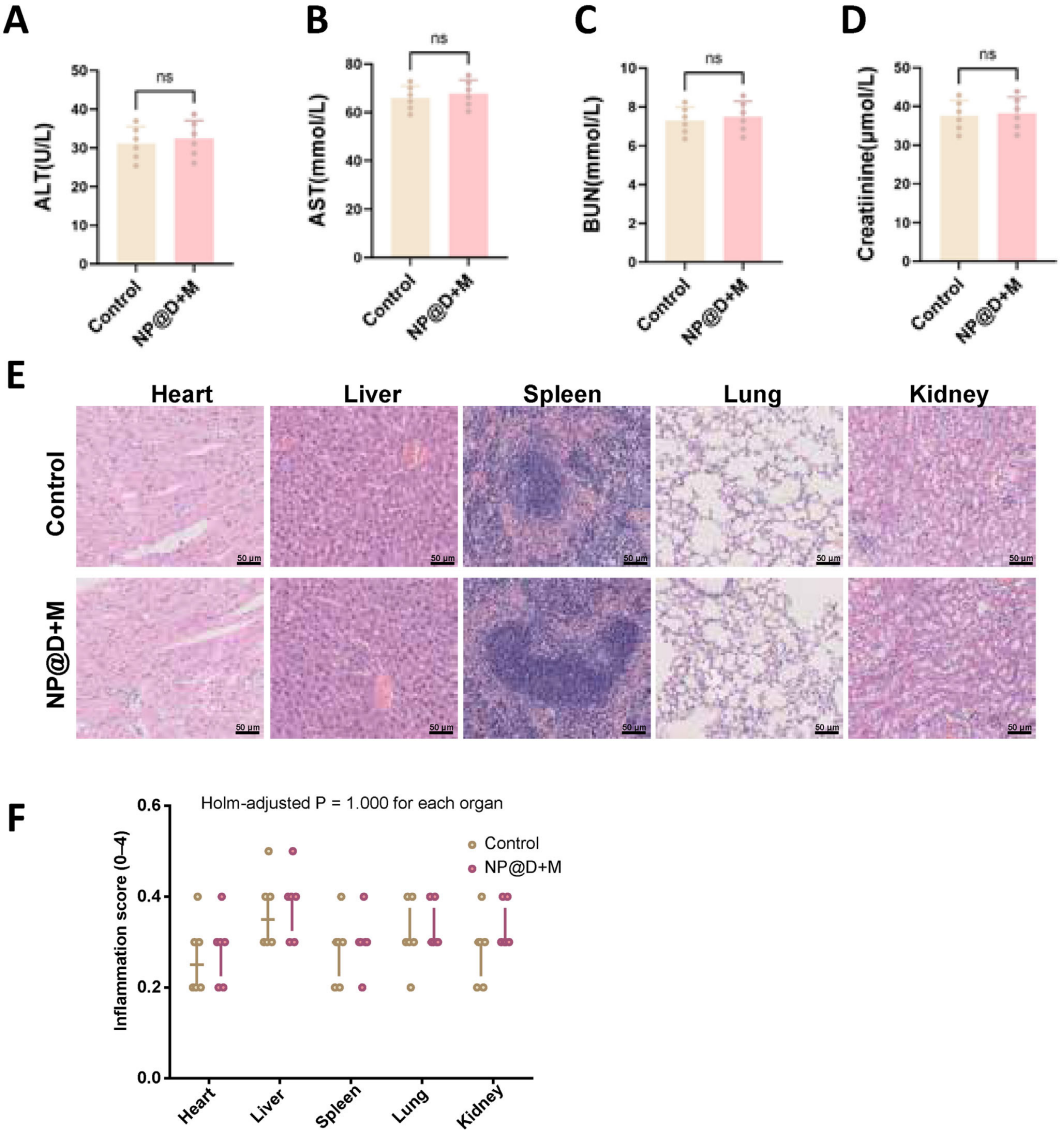
In Vivo safety evaluation of NP@D+M following repeated administration. Note: (A–D) Serum biochemical analyses of ALT, AST, BUN, and creatinine in Control and NP@D+M‐treated mice, showing no significant differences between groups. (E) Representative H&E‐stained sections of major organs (heart, liver, spleen, lung, and kidney) from Control and NP@D+M‐treated mice. Scale bar: 50 µm. (F) Animal‐level inflammation scores for the major organs. Each point represents one mouse. For (A–D), data are presented as mean ± SD. For (F), data are shown as individual animal values with median and interquartile range; n = 6 mice per group. Two‐sided Mann–Whitney U tests with Holm adjustment across five organs; adjusted *p* = 1.000 for each organ.

**TABLE 3:** Serum biochemical parameters following repeated administration of NP@D+M.

Parameter	Control group (n = 6)	NP@D+M group (n = 6)	*p* value
ALT (U/L)	31.2 ± 4.3	32.4 ± 4.7	>0.05
AST (U/L)	65.9 ± 5.1	67.8 ± 5.5	>0.05
BUN (mmol/L)	7.3 ± 0.7	7.5 ± 0.8	>0.05
Creatinine (µmol/L)	37.6 ± 3.9	38.2 ± 4.2	>0.05

Histological examination using H&E staining showed that cardiac, hepatic, splenic, pulmonary, and renal tissues maintained normal architecture, without evidence of inflammatory infiltration, necrosis, or structural disruption (Figure 20E). Specifically, no lobular disorganization or hepatocellular ballooning was observed in liver sections; glomeruli and renal tubules exhibited intact morphology; and myocardial fibers were orderly arranged. Animal‐level inflammation scores were below 1.0 on the 0–4 scale in both groups for all five organs, with no organ‐s*p*ecific difference detected after Holm adjustment (all adjusted *p* = 1.000) (Figure 20F). Collectively, these findings demonstrate that NP@D+M exhibits a favorable systemic safety profile following multiple intravenous administrations.

## 4 Discussion

This study centers on mitochondrial dysfunction as a pivotal pathological mechanism underlying SBI following ICH. For the first time, we propose a multi‐targeted therapeutic strategy leveraging a biomimetic, NP@D+M nanoparticle platform to concurrently mitigate excessive oxidative stress and aberrant mitochondrial dynamics. This study delineates a coherent neuroprotective mechanistic pathway. In the oxidative microenvironment of intracerebral hemorrhage, biomimetic nanoparticles show accelerated drug release under elevated H_2_O_2_ conditions and target the aberrant coupling between LPAR1 and VDAC1. This upstream molecular event further effectively suppresses excessive opening of the mitochondrial permeability transition pore, thereby restoring mitochondrial membrane potential and energy metabolic homeostasis. Ultimately, this precise organelle‐level intervention is translated into global metabolic recovery, significantly promoting neuronal survival and improving cognitive and neurological dysfunction in animals. In contrast to prior approaches that primarily focused on single‐target or single‐agent interventions [44, 45], our composite drug delivery system demonstrates marked advantages in targeting precision, multidimensional therapeutic coverage, and restoration of neurological function. These findings offer a novel direction for mitochondria‐targeted therapies in the context of ICH.

The first step toward achieving precise multi‐target intervention is to construct an efficient targeted delivery system. The physicochemical characteristics and delivery efficiency of the nanoparticle platform serve as the foundation for achieving multi‐target modulation [46, 47]. Using DLS, TEM, and zeta potential analysis, we confirmed the structural uniformity and colloidal stability of the PLGA‐PEG nanoparticles with H_2_O_2_‐dependent drug‐release behavior coated with a biomimetic membrane. Notably, membrane encapsulation significantly improved surface charge and dispersibility, enhancing nanoparticle–neuron interactions. Unlike conventional PLGA‐based or liposomal systems [48], our platform exhibited accelerated drug release under elevated H_2_O_2_ conditions that mimic oxidative features of the ICH microenvironment. This allowed for localized drug accumulation at the lesion site while minimizing systemic toxicity. Fluorescence imaging and in vivo biodistribution further revealed that the biomimetic coating not only facilitated BBB penetration but also promoted preferential accumulation in injured brain regions—an observation not previously reported in comparable studies. Free drugs usually enter cells through concentration gradient‐dependent passive diffusion, a process with relatively low efficiency and high susceptibility to clearance by membrane efflux pumps. In contrast, nanoparticles primarily enter cells through efficient endocytosis, thereby bypassing efflux pumps and delivering large amounts of drugs into cells in particulate form, resulting in higher intracellular drug accumulation [49]. The cell membrane coating on the surface of drug‐loaded nanoparticles endows them with excellent biocompatibility and homologous targeting recognition capability, greatly enhancing their affinity for target cells and promoting specific cellular uptake [50]. Although nanoparticles release drugs slowly under normal physiological buffer conditions, elevated H_2_O_2_ conditions can accelerate drug release from the formulation [51]. Therefore, drug release can be enhanced in oxidative pathological settings without attributing the response to a specific chemical‐bond cleavage mechanism. Taken together, enhanced specific endocytic uptake, biomimetic membrane‐mediated internalization, and H_2_O_2_‐associated accelerated release collectively confer much higher in vitro therapeutic efficiency to the drug‐loaded nanoparticles than to free drugs. This superior ability to penetrate biological barriers and accumulate at lesion sites provides a solid material basis for their subsequent mitochondrial protective effects.

On the basis of precise delivery, the central focus of this study shifted toward the systematic restoration of mitochondrial homeostasis. ΔΨm is a key indicator of mitochondrial functional integrity [52, 53]. Through JC‐1 staining assays, we observed a significant loss of Δψm in neurons subjected to ICH insult, which was markedly reversed by NP@D+M treatment. The recovery of the red/green fluorescence ratio surpassed that achieved with free drug administration. While earlier studies have shown that either the Drp1 inhibitor Mdivi‐1 or the VDAC1 inhibitor DIDS can partially restore Δψm, evidence supporting their combined synergistic effect has been limited [26, 54]. Our results provide robust validation for this combination strategy, underscoring the superior efficacy of dual‐target intervention in preserving mitochondrial homeostasis.

Restoration of ATP synthesis is a key indicator of mitochondrial functional recovery [8, 55]. Experimental data demonstrated that NP@D+M treatment significantly increased intracellular ATP levels, nearly reaching those of the control group and surpassing the efficacy of single‐drug interventions. Notably, this ATP restoration was closely associated with improved ΔΨm and may also reflect concurrent repair of the electron transport chain. While previous studies have seldom explored energy metabolism dysfunction after ICH from the perspective of respiratory chain integrity, our assessment of complexes I and IV activity revealed that multi‐targeted intervention effectively enhances enzymatic function, providing new mechanistic insights into mitochondrial metabolic dysregulation.

Excessive generation of ROS is a pivotal contributor to secondary injury in ICH, driving membrane potential collapse and disrupting cellular energy homeostasis. Both MitoSOX fluorescence microscopy and flow cytometry analyses confirmed that NP@D+M markedly reduced mitochondrial ROS levels, which were inversely correlated with membrane potential recovery. Co‐localization analysis further suggested a mutually reinforcing mechanism between ROS suppression and membrane potential restoration, indicating that this interplay may interrupt the vicious cycle linking oxidative stress and mitochondrial instability. To our knowledge, such a coordinated regulatory mechanism has not been systematically reported [56, 57], underscoring the mechanistic plausibility of the multi‐targeted approach proposed in this study.

The robust association between mitochondrial bioenergetic metabolism and functional recovery highlights the central role of energy homeostasis in neural repair after intracerebral hemorrhage. In this study, restoration of mitochondrial membrane potential and ATP synthesis (Figure 17) provided the essential energetic basis for neuronal survival and synaptic plasticity. Morphologically, this was reflected by the preservation of MAP2‐positive dendrites and the increased number of surviving neurons in the perihematomal region (Figures 3 and 16). Importantly, the rescue experiments after LPAR1 silencing provided decisive evidence for this link: when the mitochondrial protective effect of NP@D+M was abolished, its behavioral benefits were also lost (Figure 19). This strongly demonstrates that the improvement in motor and cognitive function directly results from the reversal of mitochondrial energy failure and the maintenance of neural circuit structural integrity. The consistent improvement across these multidimensional indicators systematically confirms the excellent efficacy of this platform in reconstructing energy metabolic balance at the subcellular level.

Further investigation revealed that the deeper molecular mechanism underlying this protective effect arises from precise regulation of the core signaling axis. Of particular importance is the central role of the LPAR1/VDAC1 axis. Through siRNA‐mediated gene silencing and Co‐IP assays, we demonstrated that upregulation of LPAR1 enhances VDAC1 activity, triggering mPTP opening, exacerbating membrane potential dissipation, and accelerating ATP depletion. NP@D+M treatment not only downregulated the expression of both LPAR1 and VDAC1 but also significantly reduced their spatial co‐localization. In contrast to prior studies that focused primarily on VDAC1 or Drp1 individually [15, 58], our findings are the first to highlight the LPAR1/VDAC1 axis as a mechanistic core of multi‐targeted intervention. This offers a novel molecular framework for understanding ICH pathology and developing more effective targeted therapies. Decoupling of this key pathway provides a molecular‐level explanation for the specific mechanism underlying mitochondrial functional recovery.

Ultimately, this precise subcellular‐level intervention was successfully translated into macroscopic neural injury repair and functional rescue. Neurological and cognitive recovery represent key clinical endpoints in evaluating therapeutic efficacy for ICH [59, 60]. Behavioral assessments across multiple domains demonstrated that the NP@D+M group significantly outperformed the free‐drug group in Garcia neurological scores, Morris water maze, Y‐maze, and NOR tests. Notably, the observed improvement in cognitive function following nanoparticle intervention marks a significant advancement, as prior studies have predominantly focused on acute neuroprotection, with limited attention to long‐term cognitive outcomes [61]. These findings provide experimental support for extending therapeutic strategies into the post‐acute rehabilitation phase.

In the in vivo setting, restoration of mitochondrial functional integrity (Figure 17) served as a functional bridge linking molecular‐level intervention to clinical functional recovery. Specifically, effective inhibition of the upstream LPAR1/VDAC1 axis (Figure 16G,H) directly promoted the repair of mitochondrial bioenergetic metabolism. This reversal of intracellular metabolic status was not merely a cytological phenomenon, but rather a core mechanistic driver of downstream neuroprotective effects, as reflected by markedly reduced neuronal apoptosis (Figure 16) and significant long‐term improvements in behavioral and cognitive function (Figure 15). More importantly, the siLPAR1‐mediated in vivo blockade experiment (Figure 19) further confirmed that when the mitochondrial protective effect was abolished, the previously observed neurological functional rescue was also lost. This causally demonstrates that the behavioral benefits directly arise from the reconstruction of mitochondrial homeostasis. By integrating these findings, we established a complete “signaling–mitochondria–behavior” logical axis, clearly delineating the therapeutic trajectory of NP@D+M. The marked reversal of behavioral deficits confirms the complete logical closure from energy metabolic reconstruction to neural circuit protection.

The integrated application of scRNA‐seq and targeted metabolomic analysis not only provided comprehensive validation of the multi‐target regulatory effects exerted by NP@D+M at the molecular level but also revealed the coupling between mitochondrial transcriptional programs in oligodendrocyte‐lineage cells and dynamic fluctuations of key metabolites. By combining single‐cell transcriptional profiling with quantitative metabolomic analysis, we systematically delineated the reconfiguration of energy metabolism following ICH. The results demonstrate that NP@D+M simultaneously modulates multiple metabolic pathways, including glycolysis and ROS clearance, thereby exhibiting a networked and cross‐layered mode of intervention. More importantly, the integrated transcriptomic and metabolomic analyses established a multidimensional network linking transcriptional regulation with metabolite accumulation and consumption. This finding provides evidence for the mechanisms underlying energy crises after hemorrhagic injury and lays a theoretical foundation for therapeutic strategies targeting energy metabolism.

Through repeated dosing and extended follow‐up in animal models, the biosafety of NP@D+M was rigorously evaluated. No significant hepatotoxicity, nephrotoxicity, or histopathological abnormalities were observed in major organs. While systemic toxicity remains a major concern in nanoparticle‐based drug delivery, the biomimetic membrane‐coated nanoplatform did not induce detectable peripheral toxicity under the tested conditions, thereby supporting the platform's translational potential.

Nevertheless, this study still has several limitations. First, the confocal imaging analysis in this study relied on manual ROI selection using ImageJ. Although morphological screening and NeuN‐based quality control were applied to ensure data reliability, the risk of operator‐dependent subjective bias cannot be completely excluded. Future studies will employ AI‐assisted image analysis platforms, such as CellProfiler, to automatically exclude non‐neuronal cells through integrated multichannel analysis and achieve objective single‐cell‐level quantification, thereby further improving data robustness. In addition, although this study demonstrated the targeted delivery advantage and neuroprotective effects of the biomimetic membrane‐coated nanoplatform in an intracerebral hemorrhage model using multiple experimental approaches, receptor‐blocking studies targeting specific membrane proteins and detailed inhibition experiments of endocytic pathways were not performed. Therefore, the evidence for precisely dissecting the ligand‐receptor‐specific recognition process at the molecular level remains insufficient. Future studies will introduce specific antagonists or more pathologically predictive advanced in vitro microphysiological systems, such as human‐derived blood‐brain barrier‐on‐a‐chip models, to further validate and clarify the deeper biological mechanisms underlying homologous targeted transport. Although encouraging short‐term safety results were observed after repeated administration in this study, with no significant abnormalities detected in liver and kidney function indicators or histopathological examination of major organs, the safety evaluation was mainly focused on the acute and subacute periods after administration. Systematic monitoring of long‐term toxicity remains lacking. Moreover, although the biomimetic membrane‐coating strategy is theoretically expected to reduce immunogenicity through homologous recognition, this study did not thoroughly investigate systemic immune responses, inflammatory cytokine storms, or the generation of anti‐drug antibodies during long‐term circulation. Furthermore, although in vivo imaging preliminarily confirmed targeted enrichment in the brain, more detailed pharmacokinetic analysis and quantitative biodistribution studies were not conducted. Therefore, future studies will focus on optimizing the standardized manufacturing process of the nanoformulation and conducting long‐term evaluations of safety, immunogenicity, and pharmacokinetics in large animal models or more human‐relevant microphysiological systems, thereby providing stronger data support for its future clinical translation.

Overall, this study addresses several critical limitations of previous research. First, it introduces a biomimetic nanoparticle system with H_2_O_2_‐dependent drug‐release behavior with high delivery efficiency and validated safety in targeting secondary injury after ICH. Second, by leveraging a multi‐targeted therapeutic strategy, it achieved comprehensive improvements in DeltaPsim, ATP production, respiratory chain activity, and ROS suppression. Third, through integrated scRNA‐seq and metabolomic analyses, it delineated the pivotal regulatory role of the LPAR1/VDAC1 axis in mitochondrial dysfunction following ICH, thereby deepening mechanistic understanding in this field. Considering the evolving regulatory landscape, such as the FDA Modernization Act 2.0, which encourages the reduction and replacement of animal testing using advanced human‐relevant systems [62], the translational trajectory of the biomimetic nanoplatform in this study will align with recent shifts in regulatory paradigms. Future evaluations should no longer focus primarily on large animal models. Instead, we plan to prioritize new approach methodologies, including human stem cell‐derived brain organoids and BBB‐on‐a‐chip models [63]. These advanced in vitro microphysiological systems can more accurately recapitulate the human neural microenvironment and the pathological features of ICH, thereby providing a more predictive, ethically acceptable, and regulatory‐aligned pathway for clinical translation.

In conclusion, our findings demonstrate that a biomimetic nanoparticle platform with H_2_O_2_‐dependent drug‐release behavior co‐delivering a VDAC1 inhibitor and Mdivi‐1 can effectively ameliorate mitochondrial dysfunction, attenuate oxidative stress, restore energy metabolism, and improve both neurological and cognitive outcomes in ICH models. This strategy exhibits substantial scientific innovation and translational promise. Nonetheless, certain limitations remain: the absence of validation in large animal models and the lack of long‐term toxicological and pharmacokinetic data. Additionally, further optimization is required for scale‐up manufacturing and formulation stability. Future work will focus on standardizing production processes, evaluating long‐term safety, and conducting multi‐center preclinical studies to advance clinical translation and explore its applicability in other neurodegenerative conditions.

## Author Contributions

**Ling Gao**: conceptualization, investigation, methodology, formal analysis, writing – original draft. **Li Peng**: investigation, validation, data curation, writing – original draft. **Xiaobin Zheng**: investigation, formal analysis, visualization. **Cong Tan**: investigation, resources. **Chuang Wang**: software, investigation. **Qijiang Xiong**: formal analysis (single‐cell transcriptomics), investigation. **Weiming Chen**: conceptualization, project administration, supervision, funding acquisition, writing – review and editing. All authors have read and approved the final manuscript. **Zhao Yang**: conceptualization, supervision, funding acquisition, writing – review and editing.

## Funding

This study was supported by the National Natural Science Foundation of China (No. 82460262) and the Joint Program on Health Science & Technology Innovation of Hainan Province (NO. WSJK2026MS221).

## Ethics Statement

All animal procedures were conducted in strict accordance with the protocol approved by the Affiliated Haikou Hospital, Xiangya School of Medicine, Central South University Institutional Animal Ethics Committee (Approval No. 2024067) and adhered to the internationally recognized 3Rs principles (Replacement, Reduction, and Refinement), with efforts made to minimize both the number of animals used and their suffering.

## Consent

The authors have nothing to report.

## Conflicts of Interest

The authors declare no conflicts of interest.

## Supporting information




**Supporting File**: advs78075‐sup‐0001‐Figures.docx.

**Supporting File**: advs78075‐sup‐0002‐VideoLegends.docx.

**Supporting File**: advs78075‐sup‐0003‐Movie1A.mp4.

**Supporting File**: advs78075‐sup‐0004‐Movie1B.mp4.

**Supporting File**: advs78075‐sup‐0005‐Movie1C.mp4.

**Supporting File**: advs78075‐sup‐0006‐Movie2A.mp4.

**Supporting File**: advs78075‐sup‐0007‐Movie2B.mp4.

**Supporting File**: advs78075‐sup‐0008‐Movie2C.mp4.

## Data Availability

All data generated or analyzed during this study are included in this article and/or its supplementary material files. Further enquiries can be directed to the corresponding author.
