# Supplementary material for: Mitochondrial Protection and Neural Repair via ROS‐Responsive Biomimetic Nanoparticles in Intracerebral Hemorrhage
Source: Adv Sci (Weinh). 2026 Oct 11:e78075. Online ahead of print. doi: 10.1002/advs.78075 (PMC13655243; doi:10.1002/advs.78075)
Supplement: Supplementary file 1 [file ADVS-9999-e78075-s003.docx]

**Supplementary Video Legends**

All videos are original representative behavioral videos prepared for supplementary material. Submission copies were trimmed and transcoded to MP4/H.264 (yuv420p, no audio) for broad compatibility and reduced file size.

Supplementary Movie 1. Representative Garcia neurological score test videos on postoperative day 7. Each group video contains short clips from posture symmetry, forelimb extension during walking, screen test, spontaneous activity, and body tactile reflex assessments.

Supplementary Movie 2. Representative Morris water maze escape latency videos on postoperative days 14-18. Videos show spatial learning and memory performance for the Control, Model, and NP@D+M groups.

Prepared files:

Supplementary Movie 1A - Garcia neurological score P7 - Control.mp4 (0.49 MB)

Supplementary Movie 1B - Garcia neurological score P7 - Model.mp4 (0.34 MB)

Supplementary Movie 1C - Garcia neurological score P7 - NP-D-M.mp4 (0.43 MB)

Supplementary Movie 2A - Morris water maze P14-P18 - Control.mp4 (0.07 MB)

Supplementary Movie 2B - Morris water maze P14-P18 - Model.mp4 (0.08 MB)

Supplementary Movie 2C - Morris water maze P14-P18 - NP-D-M.mp4 (0.09 MB)
