# Supplementary material for: Mitochondrial Protection and Neural Repair via ROS‐Responsive Biomimetic Nanoparticles in Intracerebral Hemorrhage
Source: Adv Sci (Weinh). 2026 Oct 11:e78075. Online ahead of print. doi: 10.1002/advs.78075 (PMC13655243; doi:10.1002/advs.78075)
Supplement: Supplementary file 2 [file ADVS-9999-e78075-s004.docx]

Fig.S1

Figure S1. Schematic illustration of the preparation of DIDS/Mdivi-1-loaded polymeric nanoparticle cores. The polymer and drugs were dissolved in the organic phase, followed by drug/polymer mixing, dropwise emulsification into 1% PVA under ultrasonication, solvent evaporation, centrifugation, washing, final purification, and lyophilization. The H₂O₂-dependent drug-release behavior of the resulting formulation was evaluated separately in vitro, as shown in Figure 2.

Fig.S2

Figure S2. Schematic Diagram of the Preparation Process for Biomimetic Membrane-Coated Nanoparticles.

Fig.S3

Figure S3. Experimental Design and Group Allocation for the Collagenase-Induced ICH Model in Mice.

Fig.S4

Figure S4. Purity Identification of Primary Cortical Neurons Cultured In Vitro.

Note: (A) Representative immunofluorescence images of primary cells cultured for 7 days. Blue: DAPI-labeled nuclei; green: NeuN-labeled neurons, mainly localized in the nucleus and perinuclear region; red: GFAP-labeled astrocytes. Scale bar: 100 μm. (B) Quantitative bar graph of cell purity. n = 5 independent fields. Data are presented as mean ± SD; ****p* < 0.001.
